# Global and hepatocyte-specific ablation of Bmal1 induces hyperlipidaemia and enhances atherosclerosis

**DOI:** 10.1038/ncomms13011

**Published:** 2016-10-10

**Authors:** Xiaoyue Pan, Christopher A. Bradfield, M. Mahmood Hussain

**Affiliations:** 1 Departments of Cell Biology and Pediatrics, SUNY Downstate Medical Center, 450 Clarkson Avenue, Brooklyn, New York 11203, USA; 2 Winthrop University Hospital, Mineola, New York, USA; 3 McArdle Laboratory for Cancer Research, University of Wisconsin–Madison, Madison, USA; 4 VA New York Harbor Healthcare System, Brooklyn, New York 11209, USA

## Abstract

Circadian rhythms controlled by clock genes affect plasma lipids. Here we show that global ablation of *Bmal1* in *Apoe*^*−/−*^ and *Ldlr*^*−/−*^ mice and its liver-specific ablation in *Apoe*^*−/−*^ (L*-Bmal1*^*−/−*^*Apoe*^*−/−*^) mice increases, whereas overexpression of BMAL1 in *L-Bmal1*^*−/−*^*Apoe*^*−/−*^ and *Apoe*^*−/−*^mice decreases hyperlipidaemia and atherosclerosis. Bmal1 deficiency augments hepatic lipoprotein secretion and diminishes cholesterol excretion to the bile. Further, Bmal1 deficiency reduces expression of Shp and Gata4. Reductions in Shp increase Mtp expression and lipoprotein production, whereas reductions in Gata4 diminish Abcg5/Abcg8 expression and biliary cholesterol excretion. Forced SHP expression normalizes lipoprotein secretion with no effect on biliary cholesterol excretion, while forced GATA4 expression increases cholesterol excretion to the bile and reduces plasma lipids in *L-Bmal1*^*−/−*^*Apoe*^*−/−*^ and *Apoe*^*−/−*^ mice. Thus, our data indicate that Bmal1 modulates lipoprotein production and biliary cholesterol excretion by regulating the expression of Mtp and Abcg5/Abcg8 via Shp and Gata4.

## Abstract (web summary)

Bmal1 is a key transcription factor that controls rhythmicity of diverse biological functions. Here, Pan *et al*. show that Bmal1 deficiency in mice increases lipoprotein secretion and reduces cholesterol excretion to bile, and decipher the molecular mechanisms underlying hyperlipidaemia and atherosclerosis promoted by the lack of Bmal1.

## Graphical abstract

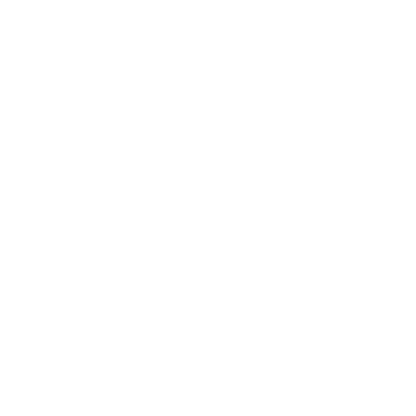

Excessive plasma triglyceride and cholesterol levels contribute to the development of several prevalent cardiovascular risk factors, such as hypertriglyceridemia, hypercholesterolemia, obesity and diabetes. Plasma triglyceride concentrations are maintained within a narrow range and exhibit circadian rhythmicity in humans and rodents^1^,^2^. Lipoprotein production is highly regulated to maintain plasma lipids. Lipoprotein production is dependent on a structural protein, apolipoprotein B (apoB), and a chaperone, microsomal triglyceride transfer protein (MTP)^3^. We have shown that plasma lipids and MTP expression exhibit in sync circadian changes and have suggested that changes in MTP expression contribute to daily variations in plasma lipids^4^. Cholesterol transported via lipoproteins is either delivered to peripheral tissues or to the liver. In the liver, cholesterol is secreted into circulation as lipoproteins or into the bile. Secretion to blood is dependent on MTP, whereas a heterodimeric complex of ATP binding cassette family G protein 5 and protein 8 (Abcg5 and Abcg8) transporters assist in the secretion of cholesterol to the bile^5^,^6^. Expression of Abcg5/Abcg8 is regulated by Lxr, Hnf4α and Gata4 (ref. 6). It is unknown whether Abcg5/Abcg8 expression changes within a day.

Daily variations in various biological, behavioral and physiological processes are controlled by several transcription factors, known as ‘clock genes', expressed in the suprachiasmatic nuclei of the brain^7^,^8^,^9^,^10^. These central clock genes form a hierarchical system to control most of the physiologic systems. Besides the suprachiasmatic nuclei, all peripheral tissues also express these clock genes^11^. This raises the question whether peripheral clock genes have autonomous function or they are subservient to the central regulatory system. Understanding the roles of peripheral clock genes in the diurnal regulation of peripheral tissues is an area of active research. The Clock and Bmal1 are two key transcription factors that increase the expression of other transcription factors to control rhythmicity of different biological functions^1^,^7^,^8^,^12^,^13^,^14^. We have examined the role of Clock by studying mice that express a dominant negative Clock mutant (Clock^Δ19/Δ19^) protein^15^. We showed that plasma triglyceride in *Clock*^*Δ19/Δ19*^ mice do not exhibit circadian rhythms, instead plasma triglyceride levels are high at all times^16^. Molecular studies showed that the Clock^Δ19/Δ19^ protein disrupts plasma triglyceride homoeostasis by de-regulating diurnal transcriptional regulation of Shp and Mtp^16^. Furthermore, we have shown that the presence of Clock^Δ19/Δ19^ protein in mice enhances atherosclerosis by increasing hepatic lipoprotein production and reducing cholesterol efflux from macrophages^17^. Clock^Δ19/Δ19^ affected the expression of MTP and ABCA1 by modulating the expression of Shp and Usf2 in hepatocytes and macrophages, respectively^17^.

Besides the Clock protein, Bmal1 is another key transcription factor acts in concert with Clock to regulate circadian mechanisms. The role of Bmal1 in circadian regulations has been gleaned from studies in global and tissue-specific Bmal1-deficient mice. Global Bmal1 deficiency results in arrhythmias, hyperglycemia and hypoinsulinemia^18^. Arrhythmias are more likely due to its critical role in the central regulation of circadian rhythms^19^. Bmal1 affects plasma glucose and insulin levels by regulating the secretion of insulin from pancreatic cells^20^. Tissue-specific deletion studies have shown that endothelial^21^, smooth muscle^22^ and bronchiolar^23^ Bmal1 affects endothelial function, blood pressure and pulmonary inflammation. Bmal1 deficiency in adipose tissues results in obesity^24^. Bmal1-deficient mice exhibit dyslipidemia^25^, but it is unknown how Bmal1 regulates plasma lipids. Transplantation of Bmal1-deficient aortic grafts into wild-type mice results in robust lesion development^26^, but it is unknown whether Bmal1 deficiency affects atherosclerosis. On the basis of these studies, we hypothesized that Bmal1 may play an important regulatory role in plasma lipid metabolism and atherosclerosis.

Here we show that Bmal1 deficiency increases hepatic lipoprotein production, cholesterol excretion to bile and atherosclerosis. Mechanistic studies show that Bmal1 regulates Shp and MTP to regulate hepatic lipoprotein production. Further, it regulates the expression of Abcg5/Abcg8 and biliary cholesterol excretion by modulating the expression of Gata4. Thus, Bmal1 is an anti-atherogenic transcription factor that controls hepatic lipoprotein production and biliary cholesterol excretion.

## Results

### Global Bmal1 deficiency increases atherosclerosis in mice

**Figure 1:**
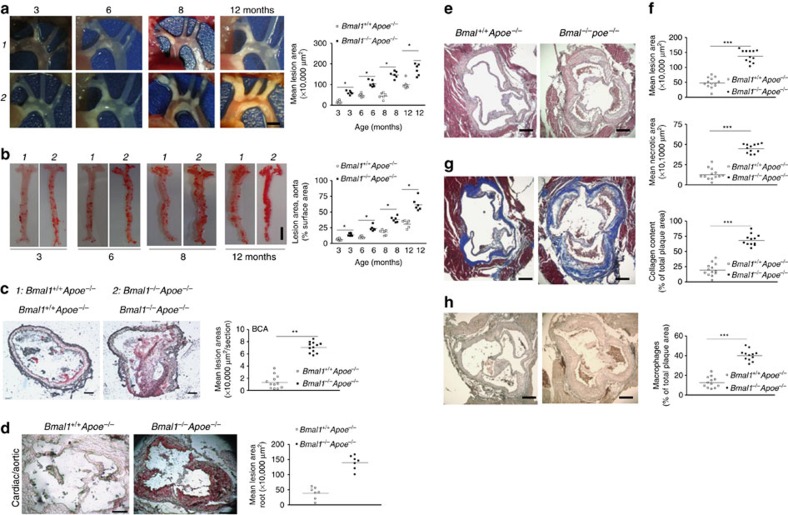
Bmal1 deficiency enhances atherosclerosis in *Apoe*^*−/−*^ mice. *Bmal1*^*−/−*^*Apoe*^*−/−*^ (black square) and *Bmal1*^*+/+*^*Apoe*^*−/−*^ (white square) male mice were fed a chow diet (*n*=6 for each age group) for 3, 6, 8 or 12 months. (**a**) Aortic arches were dissected at indicated ages and photographed. A representative picture of atherosclerotic lesion for each age group is shown (left). Scale bar, 2 mm. The plaque lesion areas were quantified in all animals and plotted (right). (**b**) Representative cardiac-aortic section stained with Oil Red O for each age is shown (left). Scale bar, 5 mm. Lipid stained areas from all animals were quantified (right). (**c**) *Bmal1*^*−/−*^*Apoe*^*−/−*^ and *Bmal1*^*+/+*^*Apoe*^*−/−*^ male mice were fed a chow diet. Section from BCA (Scale bar, 40 μm) of 8-month-old male mice were stained with Oil Red O, and quantified. (**d**–**h**) *Bmal1*^*−/−*^*Apoe*^*−/−*^ and *Bmal1*^*+/+*^*Apoe*^*−/−*^ male mice were fed a chow diet. Section from cardiac/aortic junctions (Scale bar, 400 μm) of 8-month-old male mice were stained with Oil Red O, and quantified. Sections from cardiac–aortic junctions were stained with H & E and Oil Red O to quantify lipid lesions and necrotic areas (**e**,**f**), with Masson trichrome to measure collagen content (**g**) or with anti-macrophages (AIA31240) antibodies to detect macrophages (**h**). Data in **a** and **b** are mean±s.d., *n*=6 per group, *P<0.05, one-way ANOVA; data in **c**–**h** are mean±s.d., *n*=10–12, **P<0.01, ***P<0.001, one-way ANOVA. Error bars represent s.d.

We investigated the effects of Bmal1 deficiency on atherosclerosis and observed that 3–12 months old, male *Bmal1*^*−/−*^*Apoe*^*−/−*^ mice had more aortic lesions compared with *Bmal1*^*+/+*^*Apoe*^*−/−*^ mice (Fig. 1a). Further, lipid deposition in the abdominal aorta was higher in *Bmal1*^*−/−*^*Apoe*^*−/−*^ mice of all ages (Fig. 1b). Brachiocephalic arteries (BCA) and cardiac/aortic junctions had more lipids in *Bmal1*^*−/−*^*Apoe*^*−/−*^ mice compared with *Bmal1*^*+/+*^*Apoe*^*−/−*^ mice (Fig. 1c,d). Further, atherosclerotic plaques at the cardiac/aortic junctions were enriched in necrotic core, collagen and macrophage content (Fig. 1e–h). The development of atherosclerosis was more pronounced when these mice were fed a Western diet (Supplementary Fig. 1A). Further, higher aortic lesions were also seen in *Bmal1*^*−/−*^*Ldlr*^*−/−*^ mice fed a chow and western diets (Supplementary Fig. 1B) compared with *Bmal1*^*+/+*^*Ldlr*^*−/−*^ mice. Thus, Bmal1 deficiency increases atherosclerosis in different mouse models.

### Increased hyperlipidaemia in *Bmal1* ^−/−^ *Apoe* ^−/−^ mice

To understand why Bmal1 deficiency augments atherosclerosis, we examined multiple metabolic parameters in *Bmal1*^*+/+*^*Apoe*^*−/−*^ and *Bmal1*^*−/−*^*Apoe*^*−/−*^ mice fed a chow diet. The Bmal1 deficiency significantly reduced hepatic expression of different clock genes with no effect on RORα expression in the liver (Supplementary Fig. 2A). Livers from *Bmal1*^*−/−*^*Apoe*^*−/−*^ mice had higher triglyceride and cholesterol but normal phospholipid levels compared with *Bmal1*^*+/+*^*Apoe*^*−/−*^ mice (Supplementary Fig. 2B). Plasma cholesterol ester, non-esterified fatty acids, phospholipids, glucose and insulin were higher in *Bmal1*^*−/−*^*Apoe*^*−/−*^ mice compared with *Bmal1*^*+/+*^*Apoe*^*−/−*^ mice (Supplementary Fig. 2C), but plasma leptin, AST and ALT were not different in these two groups (Supplementary Fig. 2D). *Bmal1*^*−/−*^*Apoe*^*−/−*^ mice had similar liver, intestine and kidney weights, but had significantly lower body weight and adipose tissue compared with controls (Supplementary Fig. 2E). Total plasma triglyceride, cholesterol were higher in *Bmal1*^*−/−*^*Apoe*^*−/−*^ mice of different ages compared with *Bmal1*^*+/+*^*Apoe*^*−/−*^ mice mainly due to increases in non-HDL lipoproteins (Supplementary Fig. 2F). Thus, Bmal1 deficiency causes hyperlipidaemia and hepatosteatosis. However, hepatosteatosis was not associated with increases in plasma transaminases.

**Figure 2:**
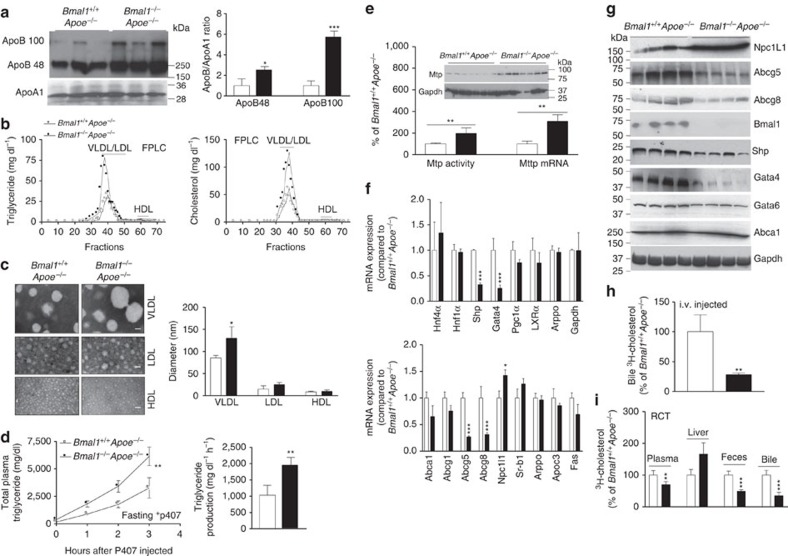
Effects of Bmal1 deficiency in *Apoe*^*−/−*^ mice. (**a**) Plasma (1 μl) was separated on SDS–PAGE and subjected to western blotting using anti-apoB, anti-ApoA1 antibodies (left). Bands corresponding to apoB100 and apoB48, and apoA1 were quantified and ApoB/ApoA1 ratios were plotted (right). (**b**) Combined (*n*=9 per group) plasma was subjected to FPLC and triglyceride and cholesterol were measured in different fractions. (**c**) VLDL, LDL and HDL prepared by ultracentrifugation were subjected to negative staining and electron microscopy (left). Diameters were quantified and plotted (right). Scale bars, 50 nm. (**d**) Overnight fasted animals were injected with P407 and plasma lipids were determined at indicated times. There were more time-dependent increases in plasma triglycerides of *Bmal1*^*−/−*^*Apoe*^*−/−*^ than *Bmal1*^*+/+*^*Apoe*^*−/−*^mice (left). The triglyceride production rates were high in *Bmal1*^*−/−*^*Apoe*^*−/−*^ than *Bmal1*^*+/+*^*Apoe*^*−/−*^mice (right). (**e**) MTP activity, protein and mRNA levels were measured in the livers. (**f**) Livers from *Bmal1*^*−/−*^*Apoe*^*−/−*^ and *Bmal1*^*+/+*^*Apoe*^*−/−*^mice were used to quantify mRNA levels of different transcription factors (top) and lipid transporters (bottom). (**g**) Western blot analysis of different transcription factors and transporters. (**h**) *Bmal1*^*−/−*^*Apoe*^*−/−*^ and *Bmal1*^*+/+*^*Apoe*^*−/−*^mice were intravenously injected with ^3^H-cholesterol. Amounts of radiolabelled cholesterol recovered in the bile collected from gall bladder after 3 h. (**i**) Amount of cholesterol found after 48 h in the plasma, liver, feces and bile after intraperitoneal placement of cholesterol loaded J774 cells in *Bmal1*^*+/+*^*Apoe*^*−/−*^and *Bmal1*^*−/−*^*Apoe*^*−/−*^mice. Data in **a**,**c**,**e**,**f**,**h** and **i** are mean±s.d., *n*=9; unpaired Student's *t*-test. *P<0.05, **P<0.01 and ***P<0.001. Data in **d** are mean±s.d., *n*=9; two-way ANOVA. **P<0.01. Error bars represent s.d.

*Bmal1*^*−/−*^*Apoe*^*−/−*^ mice had 1.5- and 4-fold more plasma apoB48 and apoB100 levels compared with *Bmal1*^*+/+*^*Apoe*^*−/−*^ mice, respectively; however, Bmal1 ablation had no effect on plasma apoAI levels resulting in higher apoB/apoAI ratio (Fig. 2a). *Bmal1*^*−/−*^*Apoe*^*−/−*^ mice had higher triglyceride and cholesterol in VLDL/LDL with no change in HDL lipids (Fig. 2b). Density gradient ultracentrifugation also showed that VLDL triglyceride, cholesterol, free cholesterol and cholesterol esters were increased in *Bmal1*^*−/−*^*Apoe*^*−/−*^ mice with no significant change in their HDL concentrations (Supplementary Fig. 3). Negative staining and electron microscopy demonstrated that *Bmal1*^*−/−*^*Apoe*^*−/−*^ mice had larger VLDL particles with diameters ranging between 80 and 140 nm, while there were no significant differences in the size of LDL and HDL particles compared with *Bmal1*^*+/+*^*Apoe*^*−/−*^ mice (Fig. 2c). These studies show that Bmal1 deficiency in *Apoe*^*−/−*^ mice increases plasma concentrations of larger lipid-rich apoB-containing lipoproteins.

### Lipoprotein production in *Bmal1* ^*−/−*^ *Apoe* ^*−/−*^ mice

To explain reasons for higher VLDL plasma lipids, we studied hepatic lipoprotein production after inhibiting lipases by the injection of poloxamer 407 (P407). Plasma triglyceride levels increased faster and remained higher at all times in lipoprotein lipase inhibited *Bmal1*^*−/−*^*Apoe*^*−/−*^ mice indicating higher triglyceride production rates (Fig. 2d). Due to the uncertainty about the effects of P407, we did not measure hepatic lipids in these mice. The effect of Bmal1 deficiency was confirmed further by studying lipoprotein production in isolated primary hepatocyte (Supplementary Fig. 4). *Bmal1*^*−/−*^*Apoe*^*−/−*^ hepatocytes secreted significantly higher amounts of triglyceride and phospholipid and had decreased cellular triglyceride with no significant differences in cellular phospholipid and protein (Supplementary Fig. 4); cholesterol mass was too low to measure. These studies indicated that *Bmal1*^*−/−*^*Apoe*^*−/−*^ hepatocytes secrete higher amounts of triglyceride-rich larger lipoproteins.

Similar studies in *Bmal1*^*−/−*^*Ldlr*^*−/−*^ mice revealed that these mice also have higher plasma triglyceride and cholesterol when fed chow or western diets compared with *Bmal1*^*+/+*^*Ldlr*^*−/−*^ mice (Supplementary Fig. 5A). Further, hepatic triglyceride production was higher in P407 injected *Bmal1*^*−/−*^*Ldlr*^*−/−*^ mice compared with *Bmal1*^*+/+*^*Ldlr*^*−/−*^ mice (Supplementary Fig. 5B). Thus, Bmal1 deficiency augments hepatic lipoprotein production and plasma lipids in both *Apoe*^*−/−*^and *Ldlr*^*−/−*^ mice.

Hepatic triglyceride-rich lipoprotein production is dependent on MTP. Livers of *Bmal1*^*−/−*^*Apoe*^*−/−*^ mice had higher MTP activity, protein and mRNA (Fig. 2e). Expression analysis of transcription factors that regulate MTP^27^ revealed that increases in MTP might be secondary to reduced expression of Shp, a repressor (Fig. 2f,g). Thus, Bmal1 deficiency might augment MTP activity, enhance VLDL production and increase plasma lipids.

### Reduced cholesterol excretion to bile in Bmal1-deficient mice

Besides VLDL production, hepatocytes efflux cholesterol by two additional mechanisms: one to HDL towards the basolateral side via Abca1 and Abcg1, and second towards the apical side to bile acids via Abcg5 and Abcg8. We found that hepatic mRNA and protein levels of Sr-b1, Abca1 and Abcg1 were unaffected; Npc1L1 were increased; and Abcg5 and Abcg8 were significantly reduced in Bmal1-deficient mice (Fig. 2f,g). Since Abcg5/Abcg8 is involved in cholesterol secretion to bile, we determined whether Bmal1 deficiency affects cholesterol efflux to bile. For this purpose, we intravenously injected [^3^H]cholesterol as lipid emulsions and studied its appearance in the bile^28^,^29^. *Bmal1*^*−/−*^*Apoe*^*−/−*^ mice had lower amounts of cholesterol in their bile compared with *Bmal1*^*+/+*^*Apoe*^*−/−*^ mice (Fig. 2h). Further, *Bmal1*^*−/−*^*Apoe*^*−/−*^ primary hepatocytes effluxed significantly lower amounts of cholesterol to bile acid acceptors (Supplementary Fig. 6). In addition, *Bmal1*^*−/−*^*Apoe*^*−/−*^ mice also had lower amounts of cholesterol in their bile and feces compared with *Bmal1*^*+/+*^*Apoe*^*−/−*^ mice (Supplementary Fig. 7). These studies showed that Bmal1 deficiency reduces the expression of Abcg5/Abcg8 and cholesterol excretion to the bile.

Delivery of cholesterol to bile is an important step in reverse cholesterol transport (RCT)^29^,^30^. Therefore, we also performed RCT by intraperitoneal injections of equal amounts of ^3^H-cholesterol loaded J774 macrophages in *Bmal1*^*−/−*^*Apoe*^*−/−*^ and *Bmal1*^*+/+*^*Apoe*^*−/−*^ mice. The amounts of cholesterol in the bile and feces were significantly less in *Bmal1*^*−/−*^*Apoe*^*−/−*^ mice compared with controls (Fig. 2i). This was not secondary to reduced delivery of cholesterol to the liver as the amounts of cholesterol delivered to the liver were similar in these mice. These studies indicated that cholesterol delivered to the liver via RCT is not excreted efficiently to the bile and feces in Bmal1-deficient mice.

Lxrα, Hnf4α and Gata4 are the major transcription factors involved in the regulation of ABCG5/G8 expression^6^. Lxrα and Hnf4α levels did not differ between the livers of *Bmal1*^*−/−*^*Apoe*^*−/−*^ and *Bmal1*^*+/+*^*Apoe*^*−/−*^mice, but *Bmal1*^*−/−*^*Apoe*^*−/−*^ livers had significantly reduced the levels of Abcg5, Abcg8 and Gata4 mRNA and protein levels (Fig. 2f,g) suggesting that Bmal1 might modulate Gata4 expression to regulate Abcg5/Abcg8 expression and biliary cholesterol secretion.

### Hepatic Bmal1 deficiency increases atherosclerosis

**Figure 3:**
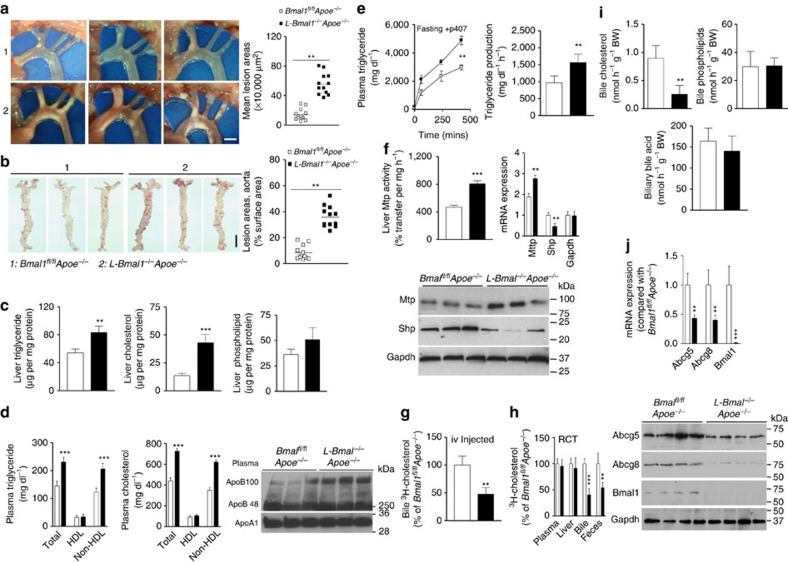
Effects of liver-specific Bmal1 ablation in *Apoe*^*−/−*^ mice. *Bmal1*^*fl/fl*^*Apoe*^*−/−*^ (white square) and *L-Bmal1*^*−/−*^*Apoe*^*−/−*^ (black square) male mice were fed a chow diet for 8 months. (**a**) Mice were dissected to visualize atherosclerotic lesions at the aortic arch and photographed (right). Plaque areas were quantified and graphed (left) and a representative cardiac-aorta stained with Oil Red O for lesion areas were quantified (right). Scale bar, 2 mm. (**b**) Whole aortas were stained with Oil Red O (left), and lesions were quantified (right). Scale bar, 5 mm. (**c**) Hepatic lipids were quantified and normalized to protein for comparison. (**d**) Triglyceride and cholesterol were measured in total plasma and in HDL after precipitating apoB-lipoproteins. Non-HDL values were deduced after subtracting HDL from total (left). Plasma apoB and apoAI were analysed by western blotting (right). (**e**) Hepatic lipoprotein production was studied after injecting P407 in 5 h fasted animals. Increases in plasma triglyceride (left) and production rates (right) were plotted. (**f**) MTP activity in liver homogenates (left), and mRNA levels of *Mttp* and *Shp* (right). Hepatic protein levels of MTP, Shp and Gapdh were detected by western blotting (bottom). (**g**) Mice were injected with ^3^H-cholesterol. Amounts of cholesterol were quantified in gall bladder bile. (**h**) J774 macrophages were incubated with 5 μCi ml^−1^ of ^3^H-cholesterol with AcLDL (50 μg ml^−1^) for 24 h. Cells were detached, washed and placed in the peritoneum of different mice. Amounts of cholesterol found after 48 h in the plasma, liver, bile and feces are shown. (**i**) Cholesterol, phospholipid and bile acid were quantified in the bile. (**j**) mRNA (top) and protein (bottom) levels of Abcg5 and Abcg8 were quantified. Data in **a** and **b** were analysed by one-way ANOVA (*n*=12 mice per group). Data in **c**,**d** and **f**–**j** were evaluated using the unpaired Student's *t*-test (*n*=12 per group). Data in **e** were examined via two-way ANOVA (*n*=4). Values are mean±s.d. *P<0.05, **P<0.01 and ***P<0.001. Error bars represent s.d.

The above studies showed that global Bmal1 deficiency increases hepatic lipoprotein production, plasma lipids and atherosclerosis while reducing cholesterol secretion to bile. To address whether this is a consequence of global Bmal1 deficiency or liver-specific function of Bmal1, we generated liver-specific Bmal1-deficient Apoe^*−/−*^mice (*L-Bmal1*^*−/−*^*Apoe*^*−/−*^). Visualization of aortic arches revealed increased lesions in chow fed *L-Bmal1*^*−/−*^*Apoe*^*−/−*^ mice compared with *Bmal1*^*fl/fl*^*Apoe*^*−/−*^ *mice* (Fig. 3a). Further, these mice had higher lipids in their aortas (Fig. 3b). Amounts of hepatic triglyceride and cholesterol, but not phospholipids, were significantly increased in *L-Bmal1*^*−/−*^*Apoe*^*−/−*^ mice compared with *Bmal1*^*fl/fl*^*Apoe*^*−/−*^ controls (Fig. 3c). *L-Bmal1*^*−/−*^*Apoe*^*−/−*^ mice had significantly higher plasma triglyceride and cholesterol in non-HDL (Fig. 3d), VLDL/LDL (Supplementary Fig. 8A) fractions. Plasma of *L-Bmal1*^*−/−*^*Apoe*^*−/−*^ mice contained higher amounts of apoB100, while apoB48 and apoAI levels were similar (Fig. 3d and Supplementary Fig. 8B). Thus, liver-specific Bmal1 deficiency causes hyperlipidaemia due to increases in apoB100-containing triglyceride and cholesterol enriched lipoproteins.

### Hepatic Bmal1 deficiency increases lipoprotein production

Physiologic studies revealed that triglyceride production rates were higher in *L-Bmal1*^*−/−*^*Apoe*^*−/−*^ mice compared with *Bmal1*^*fl/fl*^*Apoe*^*−/−*^ mice (Fig. 3e). Further, MTP activity, mRNA and protein levels were significantly increased in the livers of *L-Bmal1*^*−/−*^*Apoe*^*−/−*^ mice (Fig. 3f). We previously showed that MTP expression was increased in *Clk*^*Δ19/Δ19*^ mice due to reduced Shp expression^16^. Shp mRNA and protein levels were also reduced in the livers of *L-Bmal1*^*−/−*^*Apoe*^*−/−*^ mice compared with *Bmal1*^*fl/fl*^*Apoe*^*−/−*^ mice (Fig. 3f). These studies suggest that hepatic Bmal1 deficiency increases lipoprotein production most likely by reducing Shp and increasing MTP expression.

### Hepatic Bmal1 deficiency reduces cholesterol efflux to bile

Next, we looked at the secretion of cholesterol to the bile in *L-Bmal1*^*−/−*^*Apoe*^*−/−*^ and *Bmal1*^*fl/fl*^*Apoe*^*−/−*^ mice. Bile flow rates were similar in these mice. Fecal cholesterol levels were significantly lower in *L-Bmal1*^*−/−*^*Apoe*^*−/−*^ compared to *Bmal1*^*fl/fl*^*Apoe*^*−/−*^ mice (Supplementary Fig. 9A,B). *L-Bmal1*^*−/−*^*Apoe*^*−/−*^ mice secreted less cholesterol to bile when injected intravenously (Fig. 3g). And, *L-Bmal1*^*−/−*^*Apoe*^*−/−*^ hepatocytes effluxed less cholesterol to bile acid acceptors (Supplementary Fig. 9C). Further, mRNA levels of Gata4 and Gata6 were significantly reduced (Supplementary Fig. 9D). The amounts of cholesterol delivered from J774 macrophage to the liver were not affected but those excreted to the bile and feces during RCT were significantly reduced in these mice (Fig. 3h). Moreover, cholesterol, but not total phospholipids and bile acid, mass in the bile of *L-Bmal1*^*−/−*^*Apoe*^*−/−*^ mice was significantly less compared with controls (Fig. 3i). The mRNA and protein (Fig. 3j) levels of Abcg5 and Abcg8 were significantly decreased in the livers of *L-Bmal1*^*−/−*^*Apoe*^*−/−*^ mice compared with *Bmal1*^*fl/fl*^*Apoe*^*−/−*^ mice. These studies suggested that liver-specific Bmal1 deficiency reduces Abcg5 and Abcg8 expression to lower excretion of cholesterol to bile.

Western diet enhanced atherosclerosis in *L-Bmal1*^*−/−*^*Apoe*^*−/−*^ mice (Supplementary Fig. 10A,B). It increased hepatic triglyceride, cholesterol and cholesterol esters but had no effect on free cholesterol (Supplementary Fig. 10C). Plasma fractionation studies showed higher triglyceride and cholesterol in VLDL (Supplementary Fig. 10D). Thus, western diet augments atherosclerosis in liver-specific Bmal1-deficient *Apoe*^*−/−*^ mice.

### Effects of hepatic Bmal1 overexpression

**Figure 4:**
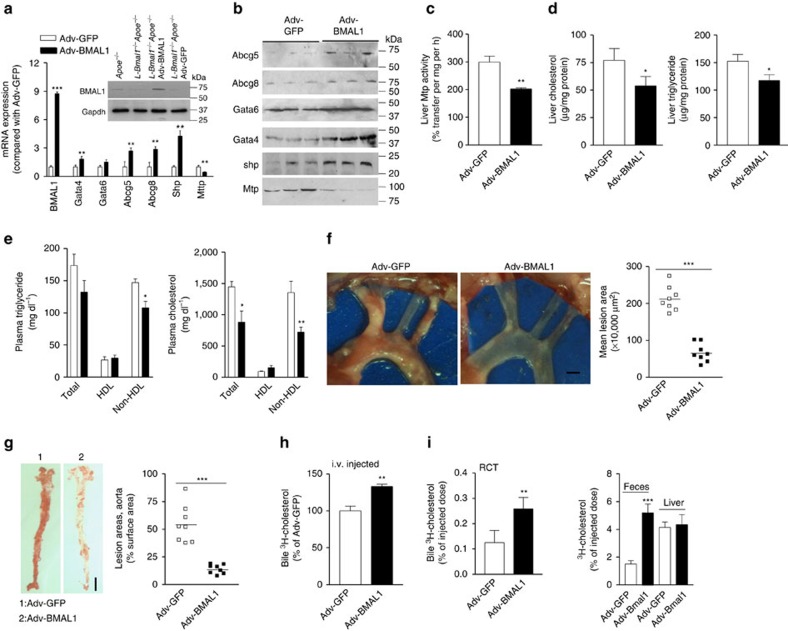
Effects of Bmal1 expression in *L-Bmal1*^*−/−*^*Apoe*^*−/−*^ mice. *L-Bmal1*^*−/−*^*Apoe*^*−/−*^ mice (3 months, male) were transduced with 1.5 × 10^11^ virus particles of either Adv-BMAL1 (black square) or Adv-GFP (white square) and started on a Western diet. After 4 weeks, plasma and tissues were collected for analysis. (**a**) mRNA levels of different indicated genes were quantified. Inset shows Bmal1 protein in the livers of different mice. (**b**) Different proteins were detected in the livers of *L-Bmal1*^*−/−*^*Apoe*^*−/−*^ mice transduced with Adv-BMAL1 or Adv-GFP. (**c**,**d**) Hepatic MTP activity (**c**), cholesterol and triglyceride (**d**) were measured. (**e**) Plasma triglyceride and cholesterol were measured in total plasma and different lipoprotein fractions after separation by precipitation. (**f**) Aortic arches were dissected, photographed (left) and lesions areas were quantified (right). Scale bar, 2 mm. (**g**) Aortas were stained with Oil Red O (left) and quantified (right). Scale bar, 5 mm. (**h**) Amounts of cholesterol excreted to bile after intravenous injection (*n*=4 per group). (**i**) Amounts of cholesterol in the bile, feces, liver and plasma 48 h after placing ^3^H-cholesterol-loaded J774 macrophages in the peritoneal cavity of different mice (*n*=4 per group). Data in **a**,**c–e**, **h** and **i** were evaluated using the unpaired Student's *t*-test. Data in **e** and **f** were tested using one-way ANOVA. Values are mean±s.d., *n*=8 per group. *P<0.05, **P<0.01, and ***P<0.001. Error bars represent s.d.

To determine further the role of hepatic Bmal1, we expressed human BMAL1 using adenoviruses (Adv-BMAL1) in western diet fed *L-Bmal1*^*−/−*^*Apoe*^*−/−*^ mice. After 4 weeks of transduction, hepatic BMAL1 mRNA and protein levels were ∼9-fold higher compared with controls (Fig. 4a). Overexpression of BMAL1 had no effect on Gata6 mRNA; increased Gata4, Abcg5, Abcg8 and Shp; and decreased MTP mRNA and protein (Fig. 4a,b) as well as activity (Fig. 4c). Further, it significantly reduced hepatic cholesterol and triglyceride compared with mice transduced with Ad-GFP control virus (Fig. 4d). BMAL1 expressing mice had lower plasma triglyceride and cholesterol due to reductions in non-HDL (Fig. 4e). Moreover, these mice had ∼75% less atherosclerosis (Fig. 4f,g). Overexpression of Bmal1 increased cholesterol and bile acids in the bile but had no effect on phospholipids. Further, it increased fecal cholesterol but had no effect on bile acids (Supplementary Fig. 11). Cholesterol efflux to bile after intravenous injection was increased in BMAL1 expressing mice compared with GFP expressing mice (Fig. 4h). Further, amounts of cholesterol delivered to the bile and feces from J774 cells during RCT were increased in Bmal1 expressing mice (Fig. 4i). Similar observations were made in *Bmal1*^*fl/fl*^*Apoe*^*−/−*^ mice after overexpressing Bmal1 (Supplementary Fig. 12). Thus, hepatic over expression of Bmal1 lowers plasma lipids, mitigates atherosclerosis and enhances biliary cholesterol secretion in *L-Bmal1*^*−/−*^*Apoe*^*−/−*^ and in *Bmal1*^*fl/fl*^*Apoe*^*−/−*^ mice. In short, these studies involving both hepatic Bmal1 ablation and over expression indicate that hepatic Bmal1 is a major regulator of plasma and tissue lipid levels.

### Effects of forced hepatic SHP expression

**Figure 5:**
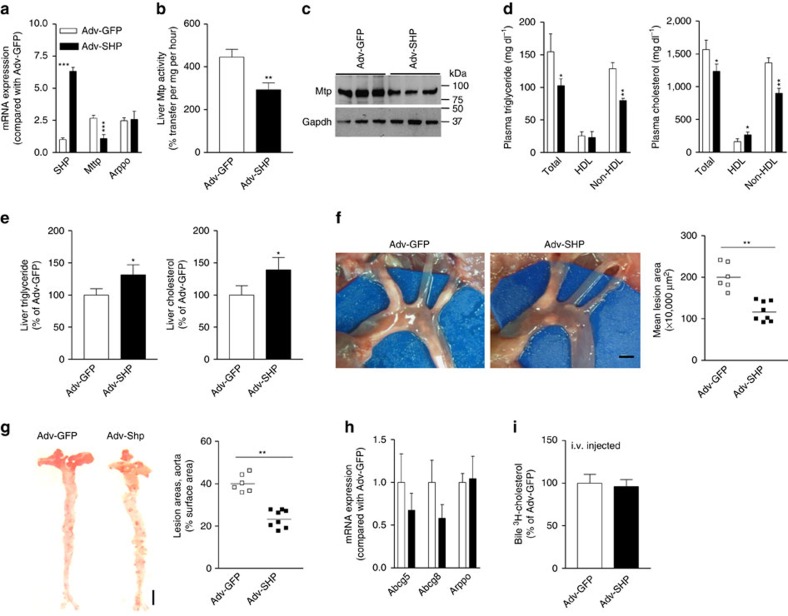
Effects of hepatic Shp expression in *L-Bmal1*^*−/−*^*Apoe*^*−/−*^ mice. *L-Bmal1*^*−/−*^*Apoe*^*−/−*^ mice (male, 12 weeks) were intravenously (i.v.) injected (1 × 10^11^ p.f.u. per mouse) with adenoviruses expressing SHP (Adv-SHP, black square) or GFP (Adv-GFP, white square) and fed a Western diet for 4 weeks. (**a**) Hepatic mRNA levels of different genes. (**b**,**c**) Livers from these mice were used to measure MTP activity (**b**) and protein (**c**). (**d**) Triglyceride and cholesterol were measured in total plasma and different lipoprotein fractions. (**e**) Lipids were measured in the livers. (**f**) The aortic arches were exposed, photographed (left) and lesion areas were quantified (right). Scale bar, 2 mm. (**g**) Aortas were dissected, stained with Oil Red O (left), and lesion areas were quantified (right) with Image-Pro. Scale bar, 5 mm. (**h**) Hepatic Abcg5 and Abcg8 mRNA levels were unaltered by Shp overexpression. (**i**) Excretion of injected ^3^H-cholesterol to the bile was unaffected by Shp expression (*n*=4 per group). Data in **a**,**b**,**d**,**e**,**h** and **i** were evaluated by the unpaired Student's *t*-test. Data in **f** and **g** were tested via one-way ANOVA. Values are mean±s.d. from 6 to 8 mice. *P<0.05, **P<0.01 and ***P<0.001. Error bars represent s.d.

Bmal1 ablation studies indicated that Bmal1 might regulate Shp to affect MTP expression and hepatic lipoprotein production. To determine whether Shp is an intermediary transcription factor used by Bmal1 to control plasma lipids, we injected *L-Bmal1*^*−/−*^*Apoe*^*−/−*^ mice with adenoviruses expressing human SHP (Adv-SHP) or green fluorescence protein (Adv-GFP) and started on a Western diet. Four weeks later, *L-Bmal1*^*−/−*^*Apoe*^*−/−*^ mice transduced with Adv-SHP had six-fold higher hepatic SHP expression (Fig. 5a) and ∼50% lower MTP mRNA, activity, and protein (Fig. 5a–c). SHP expressing mice had significantly lower plasma triglyceride and cholesterol mainly in non-HDL particles (Fig. 5d) most likely due to lower hepatic lipoprotein production (Supplementary Fig. 13). In contrast, hepatic triglyceride and cholesterol were increased (Fig. 5e). Further, SHP expressing mice had ∼50% less atherosclerotic lesions compared with controls (Fig. 5f,g). Gene expression analysis showed that SHP overexpression had no effect on Abcg5 and Abcg8 mRNA (Fig. 5h) and biliary cholesterol secretion (Fig. 5i). Similar results were obtained in *Bmal1*^*fl/fl*^*Apoe*^*−/−*^ mice transduced with Adv-SHP (Supplementary Fig. 14). Thus, Shp is involved in the regulation of MTP and VLDL production but not in the regulation of biliary cholesterol secretion in these mice.

### Bmal1 regulates Gata4 to modulate Abcg5

**Figure 6:**
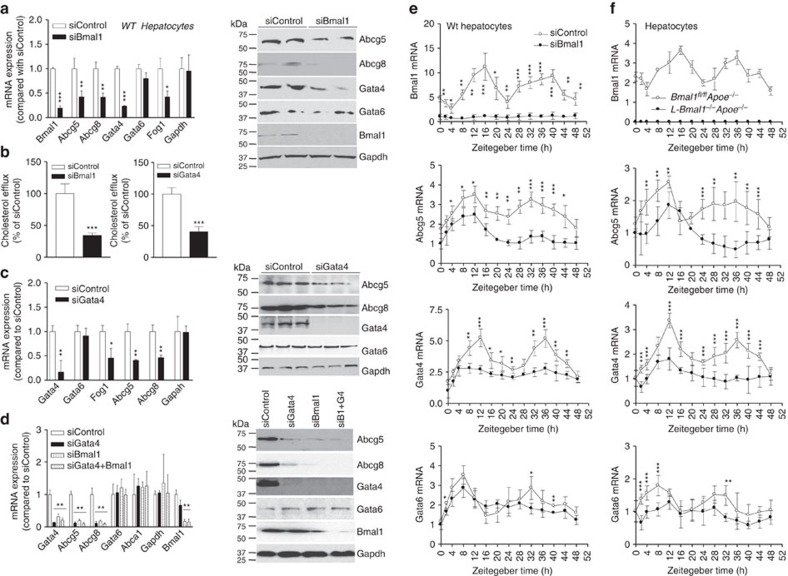
Bmal1 regulates cholesterol excretion to bile by modulating Gata4 expression. (**a**) Wild-type primary hepatocytes were transfected in triplicate with siControl or siBmal1 and different mRNA levels were measured after 48 h (left). In a separate study, hepatocytes were transfected and used to detect protein levels (right). (**b**) Cholesterol efflux to bile acid acceptors was measured in wild-type hepatocytes transfected with siControl or siBmal1 (left). Also, primary hepatocytes were transfected with siControl or siGata4 (right). After 48 h, cells were labelled with ^3^H-cholesterol, washed and incubated with media containing 10 mM TUDC for 60 min. Radioactivity was determined by scintillation counting. (**c**) Wild-type hepatocytes were transfected with siGata4 or siControl and mRNA (left) and protein (right) levels of different indicated genes were quantified. (**d**) Primary hepatocytes were transfected with siControl, siGata4, siBmal1 or siBmal1+siGata4. After 48 h, mRNA (left) and protein (right) levels of indicated proteins were quantified. (**e**) Wild-type primary hepatocytes were transfected with siControl or siBmal1. After 48 h, cells were subjected to 2 h serum shock. At indicated times; mRNA levels of different genes were measured and corrected with 18S rRNA. The values in one well were normalized to 1. Other values represent fold-change compared with 0 h. (**f**) Hepatocytes from *L-Bmal1*^*−/−*^*Apoe*^*−/−*^ and *L-Bmal1*^*fl/fl*^*Apoe*^*−/−*^ mice (3 months, male) were cultured and subjected to serum shock and changes in mRNA levels of indicated genes were quantified at indicated times. Data in **a**–**d** were tested using the unpaired Student's *t*-test. Data in **e** and **f** were examined by two-way ANOVA. Values are mean±s.d. *P<0.05, **P<0.01 and ***P<0.001. Error bars represent s.d.

Since Shp expression had no effect on cholesterol efflux to bile in *L-Bmal1*^*−/−*^*Apoe*^*−/−*^ mice, we hypothesized that Bmal1 might regulate this pathway involving another transcription factor. To test this, wild-type primary hepatocytes were transfected with siBmal1 and changes in several candidate genes known to regulate Abcg5/Abcg8 were quantified. SiBmal1 significantly reduced Bmal1, Abcg5, Abcg8, Gata4, Fog1 (a Gata4 response gene); but had no effect on Gata6 and Gapdh mRNA and protein levels (Fig. 6a). Further, it reduced cholesterol efflux from hepatocytes to bile acid acceptors (Fig. 6b, left). Similar reductions in gene expression and cholesterol excretion to bile acid acceptors were observed in siBmal1-treated human hepatoma Huh-7 cells (Supplementary Fig. 15). These studies indicated that Bmal1 deficiency reduces Gata4, Abcg5, Abcg8 expression and cholesterol efflux to bile acid acceptors in liver cells.

To evaluate the role of Gata4 in the regulation of Abcg5 and Abcg8, wild-type hepatocytes were treated with siGata4. SiGata4 decreased cholesterol efflux to bile acid acceptors (Fig. 6b right); reduced Gata4, Abcg5, Abcg8, and Fog1 expression; and had no effect on Gata6 and Gapdh mRNA and protein levels (Fig. 6c). These studies suggest that Gata4 regulates Abcg5 and Abcg8 expression and cholesterol efflux to bile acids.

To determine whether Bmal1 acts via Gata4 to regulate Abcg5 and Abcg8, hepatocytes were transfected with siBmal1, siGata4 or siBmal1+siGata4. SiBmal1 significantly reduced Bmal1, Gata4, Abcg5 and Abcg8 without affecting Gapdh, Abca1 and Gata6 mRNA and protein levels (Fig. 6d). As expected, siGata4 reduced its own expression as well as that of Abcag5 and Abcg8, but had no effect on Gapdh, Abca1 and Gata6. A combination of siBmal1+siGata4 reduced the levels of Abcg5 and Abcg8 to the same extent as individual siRNAs indicating that both Bmal1 and Gata4 are in the same pathway.

### Bmal1 modulates cyclic expression of Abcg5 and Gata4

Next, we asked whether Abcg5 shows cyclic expression and whether Gata4 is involved in the rhythmic regulation of Abcg5 by Bmal1. wild-type hepatocytes were transfected with siControl or siBmal1 and then treated with 50% serum for 2 h. Cyclic expression of candidate genes was followed over time in normal media. Bmal1 expression showed cyclic expression with first peak at 12–16 h followed by a second peak at 36–40 h. SiBmal1 significantly reduced Bmal1 expression and residual levels did not show cyclic changes (Fig. 6e). SiControl-treated cells showed peak expressions of Abcg5 and Gata4 expression at ∼12 h after serum supplementation and a second peak at ∼36 h (Fig. 6e). Gata6 mRNA exhibited peak expressions at 8 and 32 h. In siBmal1-treated cells, peak expression levels of Abcg5 and Gata4 at 12 h were significantly reduced. However, there were more pronounced reductions in their expressions at the second 36 h peak. SiBmal1 had no significant effect on the peak expression of Gata6 at 8 h and appears to dampen second peak expression at 32–36 h. These studies indicate that Abcg5, Abcg8 and Gata4 exhibit cyclic changes in hepatocytes after serum synchronization and these changes are diminished in the absence of Bmal1.

Further, we studied the cyclic expression of Bmal1, Abcg5, Gata4 and Gata6 in primary hepatocytes isolated from *Bmal1*^*+/+*^*Apoe*^*−/−*^ and *Bmal1*^*−/−*^*Apoe*^*−/−*^ mice (Supplementary Fig. 16), and in *L-Bmal1*^*−/−*^*Apoe*^*−/−*^ and *Bmal1*^*fl/fl*^*Apoe*^*−/−*^ mice (Fig. 6f). As expected, Bmal1 showed cyclic expression in *Bmal1*^*fl/fl*^*Apoe*^*−/−*^ and in *Bmal1*^*+/+*^*Apoe*^*−/−*^ but not in *L-Bmal1*^*−/−*^*Apoe*^*−/−*^ and *Bmal1*^*−/−*^*Apoe*^*−/−*^ mice. Abcg5 expression showed two peaks in the *Bmal1*^*fl/fl*^*Apoe*^*−/−*^ and *Bmal1*^*+/+*^*Apoe*^*−/−*^ hepatocytes. In *L-Bmal1*^*−/−*^*Apoe*^*−/−*^ and *Bmal1*^*−/−*^*Apoe*^*−/−*^ hepatocytes, the first peak was lower and the second peak was absent. In *L-Bmal1*^*−/−*^*Apoe*^*−/−*^ and *Bmal1*^*−/−*^*Apoe*^*−/−*^hepatocytes, peak expressions levels of Gata4, but not Gata6, were significantly lower compared with control *Bmal1*^*fl/fl*^*Apoe*^*−/−*^ and *Bmal1*^*+/+*^*Apoe*^*−/−*^ hepatocytes. Thus, Bmal1 deficiency appears to dampen the peak expression of Gata4; in contrast it abolishes the second peak expression of Abcg5 seen in control hepatocytes.

**Figure 7:**
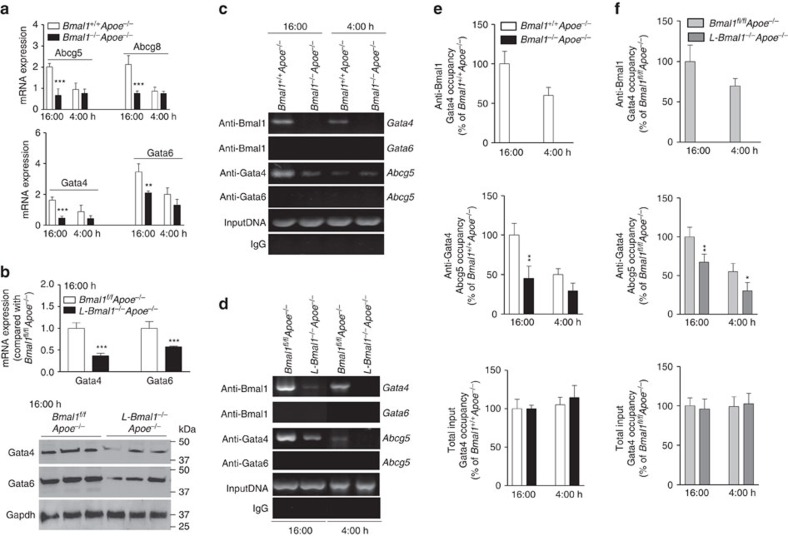
Bmal1 interacts with the promoter to regulate Gata4 expression. (**a**) Liver samples were collected at 4:00 h and 16:00 h from *Bmal1*^*−/−*^*Apoe*^*−/−*^ and *Bmal1*^*+/+*^*Apoe*^*−/−*^ mice to measure temporal changes in the mRNA levels of Abcg5, Abcg8 (top), Gata4 and Gata6 (down). (**b**) Liver samples were collected at 16:00 h from *L-Bmal1*^*−/−*^*Apoe*^*−/−*^ and control mice to measure mRNA (top) and protein (bottom) levels of Gata4 and Gata6. (**c**) Liver samples from *Bmal1*^*−/−*^*Apoe*^*−/−*^ and *Bmal1*^*+/+*^*Apoe*^*−/−*^ mice were collected at different times to study the binding of different transcription factors using indicated antibodies to the promoters of *Gata4*, *Gata6*, and *Abcg5* genes by ChIP. For this purpose, proteins were cross-linked to DNA, sheared and used to immunoprecipitate protein/DNA complexes using specific antibodies against indicated transcription factors. Sequences in the promoters of *Gata4*, *Gata6* and *Abcg5* were amplified using specific primers (Supplementary Table 1), separated on agarose gels and photographed. Representative of three experiments. (**d**) Liver from *Bmal1*^*fl/fl*^*Apoe*^*−/−*^ and *L-Bmal1*^*−/−*^*Apoe*^*−/−*^ mice were collected at 16:00 h and at 4:00 h to study the binding of Bmal1, Gata4 or Gata6 to the promoters of *Gata4*, *Gata6* and *Abcg5* genes by ChIP (representative of three different experiments). (**e**,**f**) Occupancy of Bmal1 on the Gata4 promoter and the occupancy of Gata4 on the Abcg5 promoter was studied by ChIP and then quantified by qRT–PCR in the livers of *Bmal1*^*−/−*^*Apoe*^*−/−*^ and *Bmal1*^*+/+*^*Apoe*^*−/−*^ (**e**) and *Bmal1*^*fl/fl*^*Apoe*^*−/−*^ and *L-Bmal1*^*−/−*^*Apoe*^*−/−*^ (**f**) mice. Data in **a**,**b**,**e** and **f** were examined using the unpaired Student's *t*-test. Values are mean±s.d., *n*=6 per group. *P<0.05, **P<0.01 and ***P<0.001. Error bars represent s.d.

Next, we asked whether expressions of Abcg5, Abcg8 and Gata4 change within a day and whether these changes are controlled by Bmal1 in mice. In *Bmal1*^*+/+*^*Apoe*^*−/−*^ livers, high and low expressions of Abcg5, Abcg8, Gata4 and Gata6 were seen at 16:00 and 4:00 h, respectively (Fig. 7a). In *Bmal1*^*−/−*^*Apoe*^*−/−*^ livers, the peak expressions of these genes at 16:00 h were significantly reduced but not the nadirs seen at 4:00 h. Similar to global deficiency of Bmal1, liver-specific deficiency also significantly reduced mRNA and protein levels of Gata4 and Gata6 at 16 h (Fig. 7b). These studies indicated that Bmal1 augments expression of these genes just before the nighttime suggesting that Bmal1 contributes to their peak expressions.

### Bmal1 binds to the Gata4 and Gata4 interacts with the Abcg5 promoter

The above studies suggested that Bmal1 might regulate Abcg5 and Abcg8 expression via Gata4 to modulate cholesterol excretion to bile. We then asked whether Bmal1 can interact with the *Gata4* promoter. Bioinformatics analysis revealed several E-boxes in the promoter of Gata4. We concentrated on the one E-box that was proximal to the transcription start site in the promoter of *Gata4* (Supplementary Fig. 17A); therefore, we performed semi-quantitative (Fig. 7c,d) and quantitative (Fig. 7e,f) chromatin immunoprecipitation (ChIP) in the livers at different times to determine the binding of Bmal1. In *Bmal1*^*+/+*^*Apoe*^*−/−*^ livers, the binding of Bmal1 to *Gata4* E-box was high at 16:00 h than at 4:00 h (Fig. 7c,e). In *Bmal1*^*−/−*^*Apoe*^*−/−*^ liver, as expected, Bmal1 was not associated with the promoter. Bmal1 did not appear to bind the *Gata6* promoter (Fig. 7c). We then studied the binding of Gata4 and Gata6 to the *Abcg5* promoter that contains a GATA-box (Supplementary Fig. 17B). In *Bmal1*^*+/+*^*Apoe*^*−/−*^ control mice, Gata4 binding to the *Abcg5* promoter was high at 16:00 h and low at 4:00 h (Fig. 7c,e). In *Bmal1*^*−/−*^*Apoe*^*−/−*^ mice, the binding of Gata4 to the *Abcg5* promoter at 16:00 h was significantly reduced but this binding was not reduced at 4:00 h. Gata6 did not bind to the *Abcg5* promoter. Similar results were obtained in liver-specific Bmal1 ablated mice (Fig. 7d,f). Bmal1 interacted with the *Gata4*, but not with *Gata6*, promoter and this binding was not seen in *L-Bmal1*^*−/−*^*Apoe*^*−/−*^ livers. Binding of Bmal1 to Gata4 promoter was higher at 16:00 h compared with 4:00 h. Gata4 binding to the *Abcg5* promoter was high in *Bmal1*^*fl/fl*^*Apoe*^*−/−*^ mice at 16:00 h but was significantly attenuated in *L-Bmal1*^*−/−*^*Apoe*^*−/−*^ mice. These data suggest that Bmal1 interacts with *Gata4* promoter at the onset of nighttime to increase expression resulting in enhanced binding of Gata4 to the *Abcg5* promoter.

### GATA4 affects atherosclerosis and biliary cholesterol excretion

**Figure 8:**
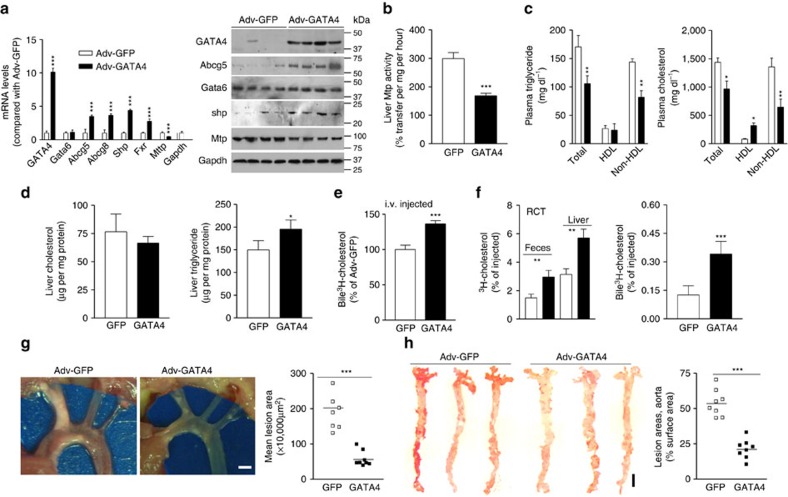
Effects of hepatic GATA4 expression in *L-Bmal1*^*−/−*^*Apoe*^*−/−*^ mice. *L-Bmal1*^*−/−*^*Apoe*^*−/−*^ mice (*n*=8, each group) were transduced with (1.5 × 10^11^ p.f.u.) Adv-GFP (white square) or Adv-GATA4 (black square) and started on a Western diet. After 4 weeks, plasma and liver were collected for different analysis. (**a**) mRNA and protein levels of indicated proteins in the liver. (**b**) Hepatic MTP activity was measured in mice transduced with different viruses. (**c**) Triglyceride and cholesterol in total plasma, HDL and non-HDL. (**d**) Liver cholesterol and triglyceride were quantified. (**e**) Amount of ^3^H-cholesterol excreted into the bile after intravenous injections. (**f**) Amounts of cholesterol found in the bile, feces and liver 48 h after the placement of ^3^H-cholesterol-loaded macrophages in the peritoneum during RCT. (**g**) Atherosclerotic plaques in the aortic branches were exposed, photographed (left) and lesion areas were quantified (right). Scale bar, 2 mm. (**h**) Aortas were stained for lipids and lesion areas were quantified. Scale bar, 5 mm. Data in **a**–**f** were evaluated using the unpaired Student's *t*-test. Data in **g** and **h** were analysed by one-way ANOVA. Values are mean±s.d., *n*=8 per group. *P<0.05, **P<0.01 and ***P<0.001. Error bars represent s.d.

Studies described above indicated that Gata4 might be an intermediary transcription factor regulating *Abcg5* and *Abcg8* gene expression by Bmal1. To test this further, *L-Bmal1*^*−/−*^*Apoe*^*−/−*^ mice were transduced with Adv-GATA4 expressing human GATA4. This transduction increased GATA4 expression by 10-fold (Fig. 8a). GATA4 overexpression had no effect on Gata6 and Gapdh mRNA and protein levels, but increased Abcg5, Abcg8, Fxr and Shp mRNA and protein while decreasing MTP expression when compared with mice transduced with Ad-GFP (Fig. 8a). Further, Gata4 overexpression decreased MTP activity (Fig. 8b), and plasma triglyceride and cholesterol in non-HDL lipoproteins (Fig. 8c). Hepatic cholesterol was unaffected but triglyceride was increased (Fig. 8d). In addition, bile and fecal cholesterol levels were increased after the overexpression of GATA4 (Supplementary Fig. 18). Mice overexpressing GATA4 accumulated more cholesterol in the bile compared with mice overexpressing GFP after intravenous injection of ^3^H-cholesterol (Fig. 8e). Further, cholesterol efflux from J774 macrophages during RCT to the liver, feces and bile was higher in GATA4 expressing mice compared with GFP expressing mice (Fig. 8f). GATA4 expression reduced lesion areas in the aortic arches and lipid staining in the abdominal aortas by ∼60–75% (Fig. 8g,h). Similar effects were observed in the *Bmal1*^*fl/fl*^*Apoe*^*−/−*^ mice after injecting Adv-GATA4 mice (Supplementary Fig. 19). Thus, overexpression of GATA4 enhances hepatic expression of Abcg5 and Abcg8, and cholesterol excretion to the bile in *L-Bmal1*^*−/−*^*Apoe*^*−/−*^ and *Bmal1*^*fl/fl*^*Apoe*^*−/−*^ mice, while reducing MTP expression and atherosclerosis.

### Bmal1 regulates cholesterol excretion to bile in wild type mice

**Figure 9:**
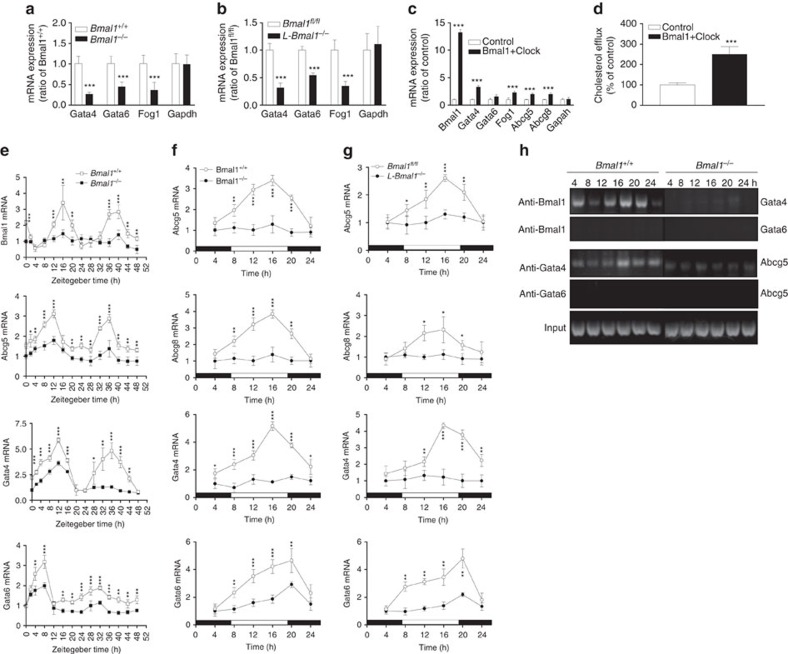
Bmal1 regulates cholesterol transporters and Gata4 in wild-type mice. (**a**,**b**) Gene expression analysis in the livers of *Bmal1*^+/+^, *Bmal1*^*−/−*^ (**a**), *Bmal1*^*fl*/fl^ and *L-Bmal1*^*−/−*^ mice (**b**). (**c**,**d**) Wild-type primary hepatocytes were transfected with plasmids expressing Bmal1 and Clock and used for gene expression studies (**c**) and for cholesterol efflux to bile acid acceptors (**d**). (**e**) Primary hepatocytes from *Bmal1*^+/+^ and *Bmal1*^*−/−*^ mice were treated with 50% serum for 2 h and collected at indicated times to measure changes in gene expression. (**f**,**g**) Livers from *Bmal1*^+/+^, *Bmal1*^*−/−*^(**f**), *Bmal1*^*fl*/*fl*^ and *L-Bmal1*^*−/−*^ mice (**g**) (six mice for each time point) were collected at indicated times to measure mRNA levels of different genes. (**h**) ChIP analysis for the binding of Bmal1 to the *Gata4* and *Gata6* promoters, and for the binding of Gata4 and Gata6 to the *Abcg5* promoter. Data in **a**–**d** were evaluated using the unpaired Student's *t*-test. Data in **e**–**g** were examined by two-way ANOVA. Values are mean±s.d. *P<0.05, **P<0.01 and ***P<0.001. Error bars represent s.d.

The above studies were performed in *Bmal1*^*+/+*^*Apoe*^*−/−*^ mice. To determine whether this regulation is specific to these mice, we studied the effect of global (*Bmal1*^*−/−*^) and liver-specific (*L-Bmal1*^*−/−*^) Bmal1 deficiency in C57Bl6J mice on the hepatic expression of Abcg5 and Gata4 (Fig. 9). Livers from *Bmal1*^*−/−*^ and *L-Bmal1*^*−/−*^ mice had significantly lower levels of Gata4, Gata6 and Fog1 compared with their respective controls (Fig. 9a,b). Overexpression of Bmal1+Clock in wild-type hepatocytes significantly increased the expression of Bmal1, Gatat4, Fog1, Abcg5 and Abcg8 (Fig. 9c), and increased cholesterol efflux to bile acid acceptors (Fig. 9d). These studies indicate that Bmal1 regulates expression of Gata4 and Abcg5.

In addition, we studied cyclic expression of these genes in *Bmal1*^*−/−*^ deficient hepatocytes subjected to serum shock (Fig. 9e). Bmal1 showed cyclic expression with two peaks at 16 and 36–40 h. Bmal1 expression was not seen in *Bmal1*^*−/−*^ hepatocytes. Abcg5 and Gata4 mRNA showed peak expressions at 12 and 36 h consistent with (Fig. 6e,f). Gata6 showed peak expressions at 8 and 32 h, and these peaks were significantly reduced in Bmal1-deficient hepatocytes. Thus, Bmal1 deficiency significantly reduced the expression of Abcg5, Gata4 and Gata6 at peak hours.

We also studied temporal changes within a day in the hepatic expression of these genes in *Bmal1*^*−/−*^ and *L-Bmal1*^*−/−*^ mice (Fig. 9f,g). Expression of Abcg5, Abcg8 and Gata4 showed maximum expression in the daytime with a peak at 16:00 h. In Bmal1-deficient animals, mRNA levels of Abcg5, Abcg8 and Gata4 were very low and they did not show significant changes within a day. Gata6 expression was also higher in the daytime but the peak was at 20:00 h. These data are consistent with CircaDB^31^. In Bmal1-deficient mice expression of Gata6 was significantly reduced and these low levels showed peak expression at 20:00 h.

Changes in the expression of these genes within a day were coincident with the binding of Bmal1 to the *Gata4* promoter and the binding of Gata4 to the *Abcg5* promoter. The binding of Bmal1 to the *Gata4* promoter was the highest at 16:00 h and low at 24:00 h (Fig. 9h). This binding was not seen in *Bmal1*^*−/−*^ livers. The binding of Gata4 to the *Abcg5* promoter was high at 16–24 h. These studies indicate that Bmal1 regulates diurnal expression of Abcg5 by upregulating Gata4 in wild-type C57Bl6J mice.

## Discussion

**Figure 10:**
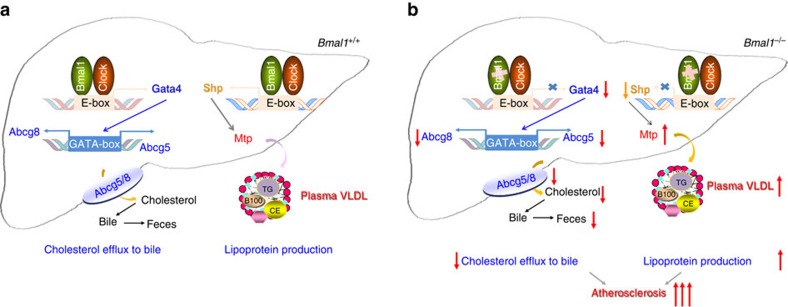
Role of hepatic Bmal1 in plasma and hepatic lipid metabolism. (**a**) Our data show that Bmal1 may regulate Gata4 and Shp to modulate two different hepatic functions. Bmal1/Clock heterodimer interacts with the E-boxes in the Gata4 and Shp promoter to enhance their expression. Increased expression of Gata4 results in enhanced binding to the GATA-box present in the Abcg5/Abcg8 promoter resulting in increased transcription. This may contribute to increased excretion of cholesterol to bile and feces. On the other hand, increased expression of Shp represses MTP expression leading to reduced lipoprotein production. (**b**) Bmal1 deficiency reduces Gata4, Abcg5 and Abcg8 expression leading to reduced cholesterol excretion into the bile. Gata4 deficiency may also reduce Shp expression and increase MTP expression and VLDL production. Increases in VLDL secretion and reductions in cholesterol excretion to the bile may additively contribute to atherosclerosis.

We used global and liver-specific Bmal1-deficient *Apoe*^*−/−*^ mice to examine the role of Bmal1 in the regulation of plasma and hepatic lipids as well as development of atherosclerosis. Both *Bmal1*^*−/−*^*Apoe*^*−/−*^ and *L-Bmal1*^*−/−*^*Apoe*^*−/−*^ mice showed increased hyperlipidaemia and atherosclerosis compared with apoE-deficient mice. Adenovirus mediated hepatic overexpression of Bmal1 in *L-Bmal1*^*−/−*^*Apoe*^*−/−*^ mice reduced hyperlipidaemia and atherosclerosis. On the basis of these knockout and overexpression studies, we conclude that hepatic Bmal1 plays a significant role in the regulation of plasma lipids and atherosclerosis. Mechanistic studies showed that hepatic Bmal1 regulates plasma and hepatic lipids by regulating Shp and Gata4 (Fig. 10a). Bmal1 deficiency reduces Shp and increases Mtp expression and VLDL production. Further, it reduces Gata4 expression leading to diminished Abcg5/Abcg8 expression and cholesterol efflux to bile (Fig. 10b). Thus, hepatic Bmal1 regulates at least two pathways (VLDL production and cholesterol efflux to bile) and disruptions in these pathways increase plasma lipids and atherosclerosis.

These studies for the first time showed that Bmal1 deficiency affects cholesterol secretion to the bile. Several lines of evidence suggest that Bmal1 regulates this pathway by modulating the expression of Abcg5/Abcg8 via Gata4. First, simultaneous knockdown of Bmal1 and Gata4 reduce Abcg5/Abcg8 to the same extent as their individual knockdowns (Fig. 6d). Second, Bmal1 deficiency reduces mRNA and protein levels of Abcg5, Abcg8 and Gata4 in the liver (Fig. 2f,g), and BMAL1 overexpression increases their levels (Fig. 4a,b). Third, temporal variations in the expression of Gata4, Abcg5 and Abcg8 were correlated with changes in Bmal1 levels within 24 h and these changes were dampened in Bmal1 deficiency (Figs 6e,f and 9e–g). Fourth, overexpression of GATA4 in Bmal1-deficient mice increased Abcg5/Abcg8 expression and cholesterol secretion to bile in *Apoe*^*−/−*^ mice (Fig. 8a,b,e,f and Supplementary Fig. 18). Fifth, Bmal1 showed time-dependent interactions with the Gata4 promoter (Fig. 7c-f). Sixth, temporal binding of Gata4 to the *Abcg5* promoter was dampened in the absence of Bmal1 (Figs 7c–f and 9h).

Excretion of cholesterol to bile is a critical step in RCT, an important physiologic process that extracts cholesterol from peripheral tissues, mainly macrophages, delivers it to the liver for excretion out of the body^32^,^33^,^34^. We did not see significant differences in the delivery of cholesterol from macrophages to the liver in *L-Bmal1*^*−/−*^*Apoe*^*−/−*^ mice. Instead, we observed significant reduction in the excretion of macrophage-derived radiolabelled cholesterol from the liver to the bile and feces. We infer this to suggest that Bmal1 deficiency might not affect cholesterol efflux from macrophages and subsequent delivery to the liver. This is supported by the observation that hepatic Abca1 and Sr-b1 were not affected in Bmal1-deficient mice (Fig. 2g). Instead, Bmal1 deficiency selectively regulates cholesterol secretion to the bile and plasma.

Due to strong epidemiological evidence for an inverse relationship between HDL and cardiovascular disease, attempts are underway to increase HDL functionality. This is usually assayed by studying cholesterol efflux from cultured macrophages using plasma or isolated HDL. Our studies suggest that monitoring of this first step might not be sufficient. There is a possibility of defects in subsequent steps of RCT, such as we have observed for cholesterol excretion into bile in Bmal1-deficient mice. Therefore, measurement of cholesterol excretion to the feces might be needed to evaluate efficacy of new therapeutic drugs.

Bmal1 deficiency increased triglyceride concentration in the plasma. Physiological studies suggested that increases in plasma triglyceride might be secondary to overproduction of hepatic lipoproteins. Molecular studies indicated that this might be related to enhanced expression of MTP. Mechanistic studies showed that this increase might be due to reduced expression of Shp, a repressor of MTP. The critical role of Shp in the regulation of MTP and lipoprotein production was evaluated by over expressing Shp in *L-Bmal1*^*−/−*^*Apoe*^*−/−*^ mice (Fig. 5 and Supplementary Fig 13). Expression of SHP reduced MTP expression, plasma lipids and atherosclerosis supporting the hypothesis that Bmal1 regulates hepatic lipoprotein production by regulating Shp.

In global Bmal1-deficient mice, both enhanced hepatic lipoprotein production and reduced cholesterol secretion to the bile might play an important role in the development of atherosclerosis. This is based on the observations that reductions in MTP activity reduces plasma lipids and atherosclerosis^35^, and global ablation of Abcg5 and Abcg8 increases atherosclerosis^36^,^37^,^38^. However, it is unclear if reductions in cholesterol secretion to the bile could contribute to atherosclerosis in *L-Bmal1*^*−/−*^*Apoe*^*−/−*^ mice. It has been shown that the liver-specific overexpression of Abcg5 in *Abcg5*^*−/−*^ mice does not reduce plasma cholesterol and atherosclerosis most likely due to increased absorption of cholesterol in the intestine^38^. However, our studies involving overexpression of BMAL1, SHP or GATA4 in *L-Bmal1*^*−/−*^*Apoe*^*−/−*^ mice indicate that a combination of hepatic lipoprotein over production and reduced biliary cholesterol excretion might contribute additively to atherosclerosis. This is based on the observation that expression of BMAL1 or GATA4 reduced atherosclerosis to similar extents (∼75%), whereas Shp expression reduced it by ∼50%. Since, Bmal1 and Gata4 affect both lipoprotein production and cholesterol secretion to bile, it is likely that their higher efficacy in reducing atherosclerosis compared with Shp is secondary to their effects on both these processes.

It is unclear why Bmal1 coordinately regulates hepatic lipoprotein production and cholesterol excretion. It is possible that increased VLDL production might protect liver from excess cholesterol accumulation that might ensue when excretion to the bile is curtailed. This knowledge about the modulation of two functions probably indicates that Bmal1 might regulate hepatic lipid metabolism by affecting several different pathways.

By studying atherosclerosis in *Bmal1*^*−/−*^*Apoe*^*−/−*^ and *L-Bmal1*^*−/−*^*Apoe*^*−/−*^ mice we tried to learn about the contribution of hepatic Bmal1 to atherogenesis. The atherosclerotic lesion areas were smaller in *L-Bmal1*^*−/−*^*Apoe*^*−/−*^ mice indicating that Bmal1 deficiency in other tissues also contributes to atherosclerosis. In this regards, it would be interesting to explore in the future the role of intestine and macrophage-specific Bmal1 on atherosclerosis. Aortic transplantation studies have shown that aortic Bmal1 contributes to atherosclerosis. Thus, further studies can also be conducted to address the role of endothelial and smooth muscle cell Bmal1 on atherogenesis.

Our studies showed that Bmal1 deficiency does not affect hepatic Abca1 and Abcg1 levels in the liver. This was surprising as we had previously noted that Clock^Δ19/Δ19^ regulates cholesterol efflux from macrophages by modulating Abca1 expression^17^. More studies are needed to fully explain differential, tissue-specific mechanisms involved in the regulation of Abca1 by clock genes.

Bmal1 is an integrated component of the clock regulatory transcription factors that form an auto-regulatory loop. Therefore, it is possible that the effect of Bmal1 deficiency might be a reflection of disruptions in the whole circadian regulatory mechanisms. More studies are needed to find out whether the observed effects are due to Bmal1 deficiency or they are related to altered expression of downstream targets such as Pers, Crys and other transcription factors. In fact, it has been shown that Rev-erba regulates lipid metabolism by both circadian and epigenetic mechanisms^39^. Studies in mice deficient in individual or several of these transcription factors may provide information about circadian and transcription factor specific effects of clock genes in the regulation of plasma lipids and atherosclerosis.

In short, these studies show that Bmal1 deficiency affects two different hepatic pathways of cholesterol secretion. Bmal1 deficiency increases cholesterol secretion via VLDL and reduces cholesterol secretion to bile. Increased VLDL assembly and secretion is due to suppression of Shp and induction of MTP expression. The reductions in cholesterol secretion to the bile are due to lower expressions of Gata4, Abcg5 and Abcg8. Thus, Bmal1 regulates a repressor and an activator to control hepatic lipid homeostasis. It is likely that Bmal1 might regulate additional pathways and acts as a master regulator of hepatic lipid metabolism.

## Methods

### Materials

[^3^H]Cholesterol and [^3^H]glycerol were from NEN LifeScience Products. AcLDL was from Biomedical Technologies, Inc.

### Animals

Bmal1^+/−^, Bmal1^+/−^Ldlr^−/−^ and Bmal1^+/−^Apoe^−/−^ heterozygous mice were bred to obtained Bmal1^+/−^, Bmal1^−/−^, Bmal1^−/−^Ldlr^−/−^, Ldlr^−/−^, Bmal1^−/−^Apoe^−/−^ and Apoe^−/−^ siblings. To obtain liver-specific Bmal1 gene deleted (Alb-cre-Bmal1^fl/fl^, L-Bmal1^−/−^) mice, Bmal1^fl/fl^ mice were crossed with Alb-Cre (Jackson Laboratories) mice. They were then bred with Apoe^−/−^ mice to get Bmal1^fl/fl^Apoe^−/−^ and L-Bmal1^−/−^Apoe^−/−^ mice. All mice were on C57Bl/6J background. Knockout and wild-type siblings from heterozygous parents were used for studies. Global Bmal1 deficient and their controls were provided food in the bottom of their cages as these mice are unable to reach to the top for food^40^,^41^. Provision of food in the bottom of the cage prevents early death reported in these mice^42^. They were fed a chow diet (LabDiet, #5001). Unless indicated otherwise, male, 3–4 months old mice were fed a Western diet (Harland Tekland, TD88137) for atherosclerosis studies. Mice were housed with a 12-h lighting schedule (7:00–19:00 h). Animal experiments were approved by the Animal Care and Use Committee of the SUNY Downstate Medical Center.

### Other analyses

After 4 h fast, plasma was obtained to measure lipids using kits. Mice were not fasted when daily changes in plasma and tissue lipids were studied. Triglyceride, cholesterol, free cholesterol and phospholipid in tissues, bile and feces were determined using kits^4^,^16^. All the feces collected during 48 h were used for lipid extraction.

### Characterization of lipoproteins

Plasma was subjected to FPLC and different fractions were collected. In addition, plasma was subjected to sequential density gradient centrifugation to isolate VLDL, LDL and HDL. Plasma was also subjected to precipitation with tungstate/MgCl_2_ to obtain values in HDL and non-HDL fractions^43^. Lipid synthesis and secretion were also studied^43^.

### Hepatic lipoprotein production

Mice were fasted for 4 h and injected intraperitoneally with 0.5 ml of Poloxamer P407 in PBS (1:6, v/v). Plasma was collected at indicated times to measure lipids and determine triglyceride production rates^16^. MTP activity was determined using a kit^44^.

### Western blot analyses

Proteins were separated on polyacrylamide gels, transferred to nitrocellulose membranes, blocked for 2 h in 20 mM Tris, 137 mM NaCl, pH 7.5, containing 0.1% Tween 20 and 5% nonfat dry milk at room temperature. The blots were washed three times and incubated overnight at 4 °C in the same buffer containing 0.5% dry milk with different antibodies (Supplementary Table 2), washed, and then incubated with mouse horseradish peroxidase-conjugated secondary antibody (1:1,000–1:4,000) in 1% skim milk for 1 h. Immune reactivity was detected by chemiluminescence^4^,^16^,^45^ . Full scans of western blots and gels are presented in Supplementary Fig. 20.

### Electron microscopy of lipoproteins

VLDL, LDL and HDL fractions were isolated from the plasma by ultracentrifugation. Fractions were mixed 1:1 (v/v) with 2% phosphotungstate for 1 min and applied to a carbon-formvar grid^46^. Excess sample was removed by blotting, air-dried and viewed under an electron microscope.

### Cholesterol efflux from hepatocytes to bile acid acceptors

Primary hepatocytes (10 × 10^6^ cells in each well of a six-well plate) from different mice were incubated in triplicate with 1 μCi ml^−1^ of [^3^H]cholesterol for 16 h. Cells were washed and then incubated in fresh media containing 100 μM tauroursodeoxycholate (TUDC) for different time^47^. After the incubation in a humidified 37 °C incubator in the presence of 5% CO_2_, media from each well were collected and centrifuged (12,000 r.p.m., 5 min) to remove cellular debris. Aliquots of the supernatant were taken for counting radioactivity. The remaining cell-associated [^3^H]cholesterol was determined after extraction for at least 1 h with β-propanol. The radioactivity released to the medium was expressed as the fraction of the total radioactive cholesterol present in the media and cells.

### Evaluation of atherosclerosis

The proximal aorta was collected after saline perfusion. The aortic root and ascending aorta were sectioned at a thickness of 10 μm. Alternate sections were stained for lipids using Oil Red O (ref. 17). Further sections were treated with Trichrome to measure collagen deposition and with antibodies AIA31240 (Accurate Chemical and Scientific Corp) to assess macrophage infiltration^17^.

### Synchronization of cells to determine cyclic changes

Primary hepatocytes (10 × 10^6^) were plated in 12-well plates. Hepatocytes were starved in the same medium with no FBS for 18 h. They were then incubated in media containing 50% horse serum for 2 h. Media was changed to serum-free media and cells were harvested at 4 h intervals for analyses^16^,^17^.

### *In vivo* liver cholesterol biliary excretion

Separate groups of mice (*n*=6) received an intravenous (tail vein) injection of [^3^H]cholesterol (1 μCi) with unlabelled cholesterol as lipid emulsions^28^,^29^. At 4 h, mice were anaesthetized by intraperitoneal injection with ketamine and xylazine. Bile was collected by cannulation of the gallbladder for 30 min, during which body temperature was stabilized using a heating humidified incubator.

### *In vivo* reverse cholesterol transport

J774A.1 cells were radiolabelled with 5 μCi ml^−1^ of [^3^H]cholesterol and 50 μg ml^−1^ acetylated LDL for 48 h. These labelled foam cells were washed twice, equilibrated in medium with 0.2% bovine serum albumin for 8 h, centrifuged and resuspended in RPMI medium immediately before use. The washed cells (2 × 10^6^) were injected intraperitoneally into different mice housed individually with unlimited access to food and water. After 48 h of injection, mice were exsanguinated and perfused with cold PBS. Bile, feces and liver were collected and the radioactivity was measured by liquid scintillation counting (Packard Tri-Carb model 1,500 liquid scintillation counter)^4^,^17^.

### Quantitative RT–PCR

Total RNA from tissues and cells were isolated using TriZol. The first-strand complementary DNA was then synthesized using Omniscript RT (Qiagen) kit, and then used for quantitative RT–PCR using SYBR Green to quantify changes in mRNA. The data were analysed using the ΔΔC_T_ method and presented as arbitrary units^16^,^17^.

### Chromatin immunoprecipitation

ChIP^16^,^17^ was performed to study the binding of different transcription factors to the promoters of different genes using polyclonal antibodies (Supplementary Table 1). DNA samples recovered after immunoprecipitation were subjected to quantitative PCR using specific primers (Supplementary Table 2). As negative controls, ChIP was performed in the absence of antibody or in the presence of rabbit IgG. These experiments were repeated three to four times with similar results and a representative experiment is shown.

### Statistical analyses

Data are presented as mean±s.d., *n*=6–12 animals for each time point. Statistical testing was performed by the unpaired Student's *t*-test. For comparison between two groups one-way analysis of variance (ANOVA) was performed followed by Dunnett's correction. Temporal comparisons between two groups were performed using two-way ANOVA as indicated in figure legends. Differences were considered statistically significant when *P*<0.05. GraphPad Prism was used for graphing and statistical evaluations.

### Data availability

All relevant data are available from the authors upon request.

## Additional information

**How to cite this article**: Pan, X. *et al*. Global and hepatocyte-specific ablation of Bmal1 induces hyperlipidaemia and enhances atherosclerosis. *Nat. Commun.* **7**, 13011 doi: 10.1038/ncomms13011 (2016).

## Supplementary Material

Supplementary Information Supplementary Figures 1-20 and Supplementary Tables 1-2
