# Supplementary material for: Global and hepatocyte-specific ablation of Bmal1 induces hyperlipidaemia and enhances atherosclerosis
Source: Nat Commun. 2016 Oct 10;7:13011. doi: 10.1038/ncomms13011 (PMC5062545; doi:10.1038/ncomms13011)
Supplement: Supplementary Information — Supplementary Figures 1-20 and Supplementary Tables 1-2 [file ncomms13011-s1.pdf]

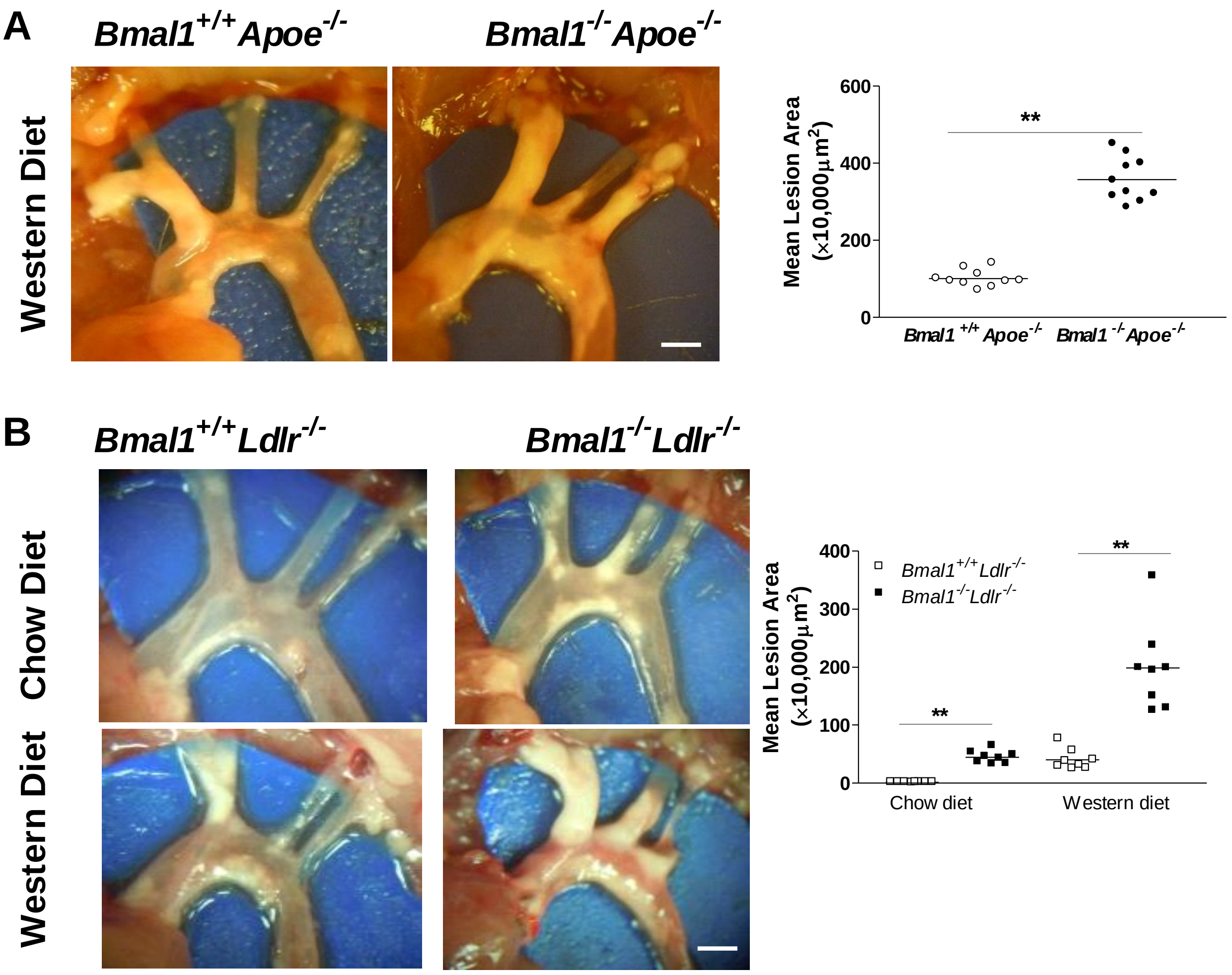

Supplementary Fig 1. Extensive atherosclerosis in Western diet fed *Bmal1*$^{-/-}$ deficient mice.

(**A**) *Bmal1*$^{-/-}$*Apoe*$^{-/-}$ and *Bmal1*$^{+/+}$*Apoe*$^{-/-}$ mice (male, 6 months old, n=10) were fed a Western diet for 2 months. Atherosclerotic lesions were photographed (left) and quantified (right). Scale bar, 2 mm. Values are mean ± SD, n=10/group, one-way ANOVA. ** p<0.01 compared with *Bmal1*$^{+/+}$*Apoe*$^{-/-}$. (**B**) *Bmal1*$^{-/-}$*Ldlr*$^{-/-}$ and *Bmal1*$^{+/+}$*Ldlr*$^{-/-}$ mice (male, 6 months old,) were fed a chow diet or Western diets for 2 months and used to measure atherosclerotic lesion. Scale bar, 2 mm.

Values are mean±SD, n=7-8/group, one-way ANOVA. ** p<0.01 compared with chow fed *Bmal1*$^{+/+}$*Ldlr*$^{-/-}$. Error bars represent SD.

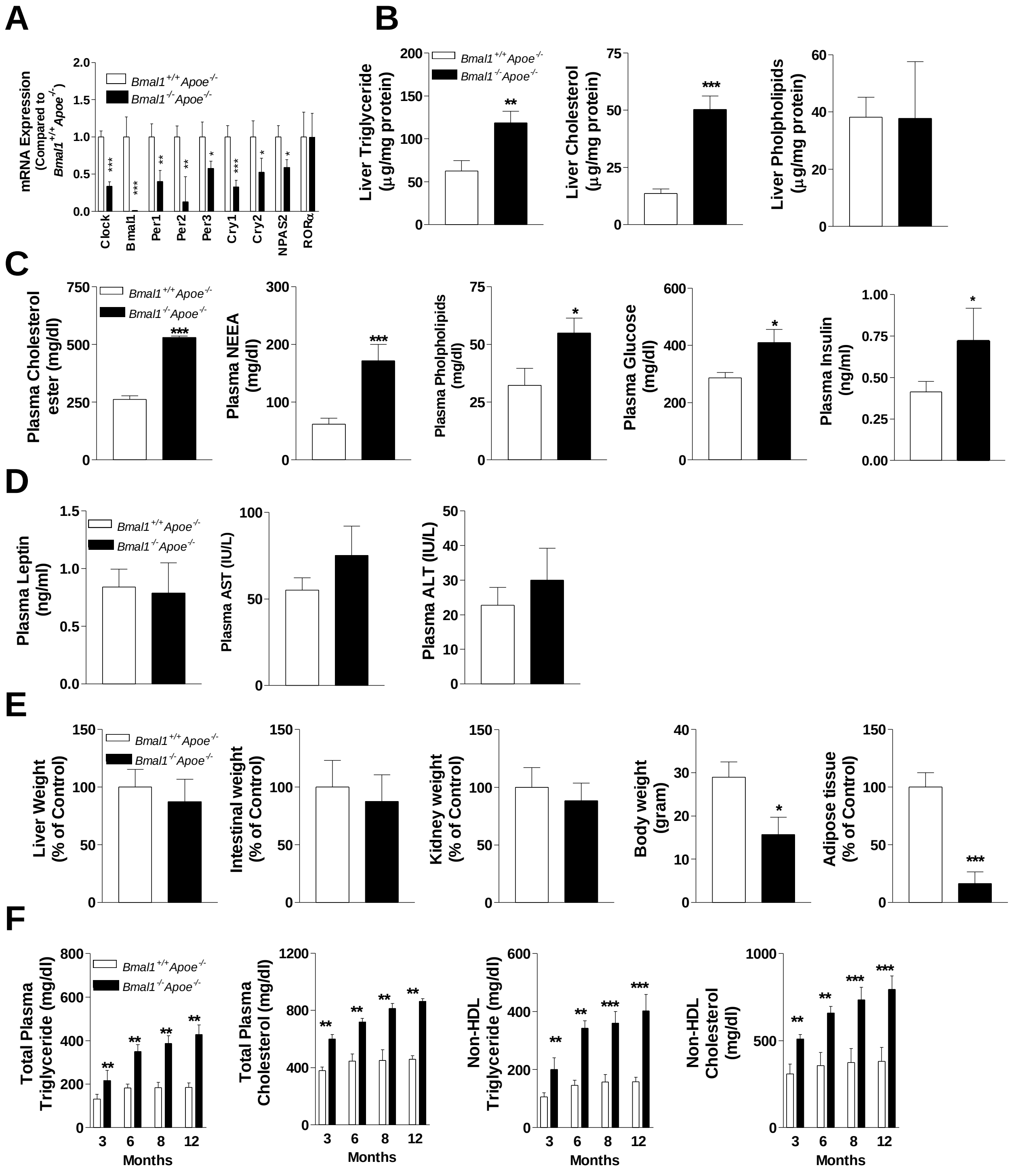

Supplementary Fig 2: Effect of Bmal1 ablation in chow fed *Bmal1*$^{+/+}$*Apoe*$^{-/-}$ mice on plasma and hepatic lipids.

(**A-B**) Quantification of clock genes (A) and lipids (B) in the livers of *Bmal1*$^{-/-}$*Apoe*$^{-/-}$ and *Bmal1*$^{+/+}$*Apoe*$^{-/-}$ mice (male, 8 months old,).

(**C-D**) Quantification of plasma lipids, glucose, insulin (C), leptin and transaminases (D).

(**E**) Differences in body and tissue weights in these mice at 8 months of age.

(**F**) Changes in plasma lipids and lipoproteins with age.

Values (A-F) are mean ± SD, n=10/group, unpaired Student's *t*-test. * $p<0.05$, ** $p<0.01$, and *** $p<0.001$ compared with *Bmal1*$^{+/+}$*Apoe*$^{-/-}$. Error bars represent SD.

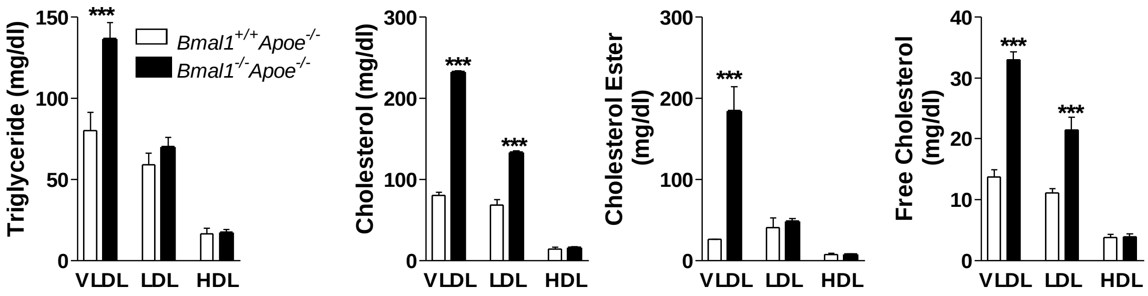

Supplementary Fig 3: Global ablation of Bmal1 enhances hyperlipidemia in different lipoprotein. *Bma1l*$^{+/+}$*Apoe*$^{-/-}$ (□) and *Bmal1*$^{-/-}$*Apoe*$^{-/-}$ (■) male mice were fed a chow diet for 8 months. Plasma was subjected to sequential ultracentrifugation to obtain different lipoproteins. Triglyceride and cholesterol were measured in purified lipoproteins. Values are mean ± SD, n=10/group, unpaired Student's *t*-test. ***$P<0.001$ vs *Bmal1*$^{+/+}$*Apoe*$^{-/-}$. Error bars represent SD.

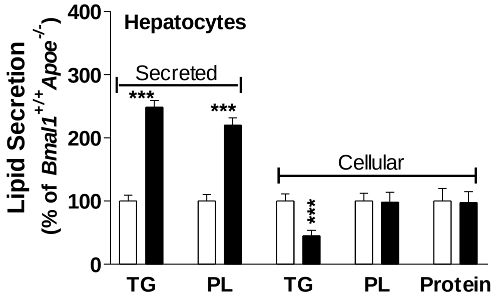

Supplementary Fig 4: *Bmal1*$^{-/-}$*Apoe*$^{-/-}$ hepatocytes secrete more lipids. *Bma1l*$^{+/+}$*Apoe*$^{-/-}$ (□) and *Bmal1*$^{-/-}$*Apoe*$^{-/-}$ (■) male mice were fed a chow diet for 8 months. Primary hepatocytes from *Bmal1*$^{-/-}$*Apoe*$^{-/-}$ and *Bmal1*$^{+/+}$*Apoe*$^{-/-}$ mice were labeled with [^{3}H]glycerol (5 µCi/ml) for 12 h. Lipids were extracted from media and cells, and separated on thin layer plates to quantify counts in triglycerides (*TG*) and phospholipids (*PL*). Protein was measured in cells after lipid extraction. Values are mean ± SD, n=6/group, unpaired Student's *t*-test. ***$P<0.001$ vs *Bmal1*$^{+/+}$*Apoe*$^{-/-}$. Error bars represent SD.

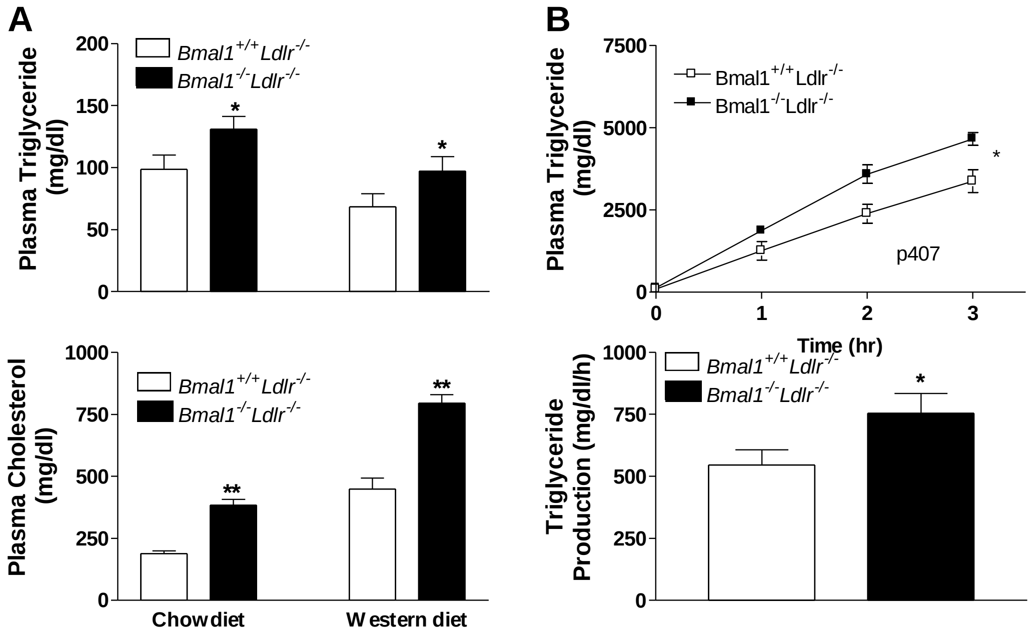

Supplementary Fig 5: Plasma lipids and lipoprotein production in *Bmal1*$^{-/-}$*Ldlr*$^{-/-}$ and *Bmal1*$^{+/+}$*Ldlr*$^{-/-}$ mice.

(**A**) Plasma triglyceride and cholesterol in mice (male, 6 months old) fed a chow or Western diet for 2 months. Values are mean ± SD, n=8, unpaired Student’s *t*-test. *P<0.05, and **P<0.01 vs *Bmal1*$^{+/+}$*Ldlr*$^{-/-}$.

(**B**) Chow-fed mice (male, 6 months old) were fasted for 5 h, injected with P407, and plasma triglycerides were determined at indicated times (top) and production rates were deduced (bottom). Values are mean ± SD, n=4, two-way ANOVA (top) and unpaired Student's *t*-test (bottom). *P<0.05, vs. *Bmal1*$^{+/+}$*Ldlr*$^{-/-}$. Error bars represent SD.

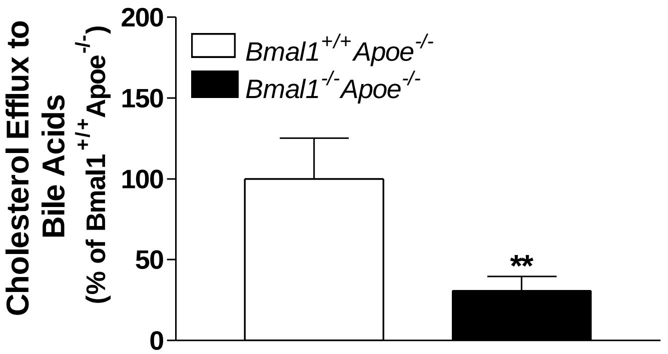

Supplementary Fig 6: Global ablation of Bmal1 decreases cholesterol excretion to bile acid acceptors in hepatocytes of *Apoe*$^{-/-}$ mice.

Bma1l$^{+/+}$*Apoe*$^{-/-}$ (□) and *Bmal1*$^{-/-}$*Apoe*$^{-/-}$ (■) male mice were fed a chow diet for 8 months. Primary hepatocytes from these mice were plated in 12 well plates. They were labeled for 16 hour with ^{3}H-cholesterol (1 µCi/ml), washed and then incubated with media containing 100 µM TUDC bile acid acceptors for 4 h. Amount of radioactivity in the cells and media were quantified. Values are mean ± SD, n=6/group, unpaired Student's *t*-test. **P<0.01 vs *Bmal1*$^{+/+}$*Apoe*$^{-/-}$. Error bars represent SD.

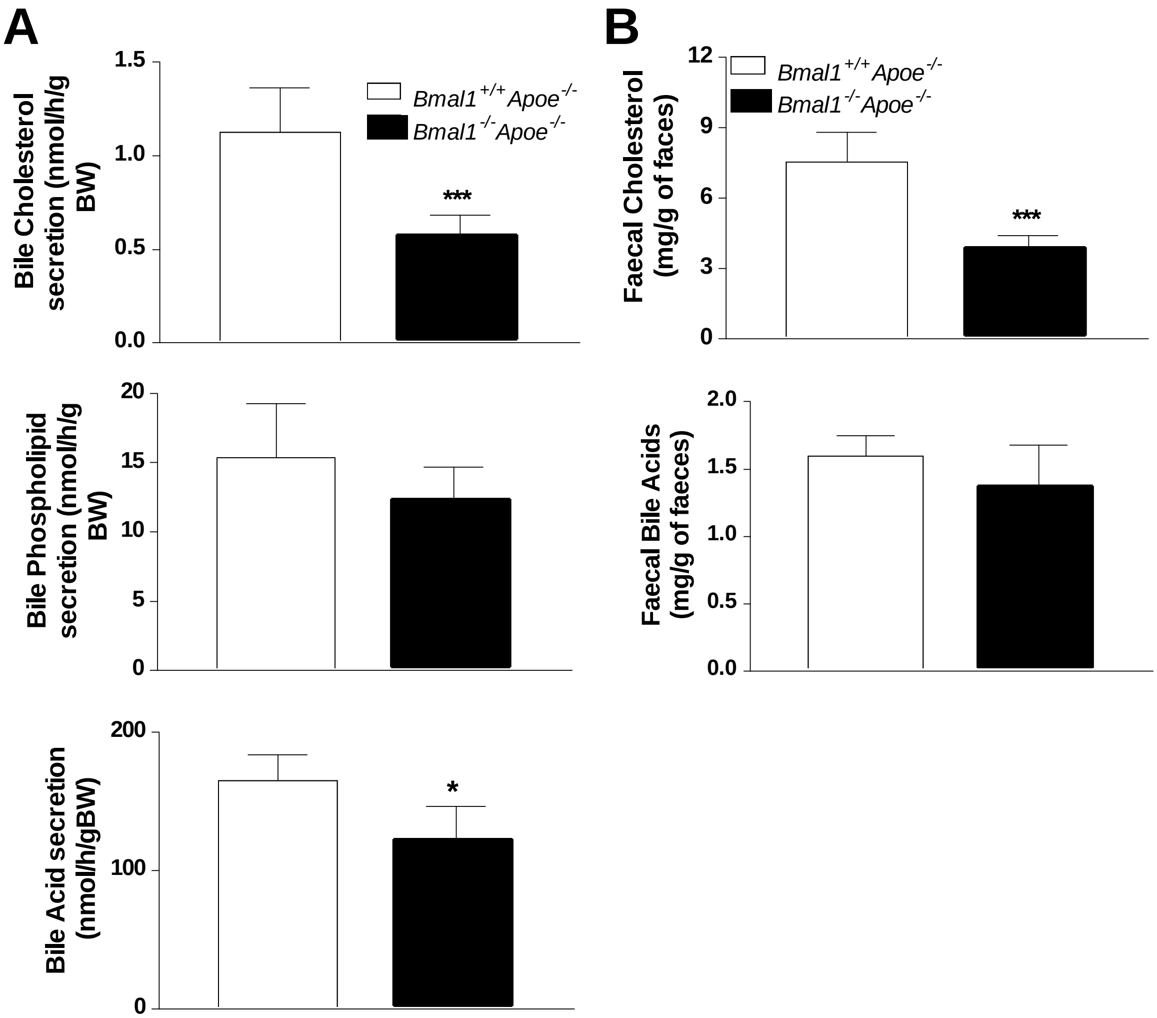

Supplementary Fig 7: Cholesterol, phospholipid, bile acid in bile and feces of *Bma1l*$^{+/+}$*Apoe*$^{-/-}$ and *Bmal1*$^{-/-}$*Apoe*$^{-/-}$ mice.

(**A**) Total cholesterol, phospholipid, bile acid secretion rate (nmol per h per gram of body weight) in *Bma1l*$^{+/+}$*Apoe*$^{-/-}$ (□) and *Bmal1*$^{-/-}$*Apoe*$^{-/-}$ (■) male mice fed a chow diet for 8 months.

(**B**) Total fecal cholesterol and bile acid excretion (mg/g of feces weight) over 48 h in 8 month old *Bma1l*$^{+/+}$*Apoe*$^{-/-}$ (□) and *Bmal1*$^{-/-}$*Apoe*$^{-/-}$ (■) male mice fed a chow diet.

Values are mean ± SD, n=6/group, unpaired Student's *t*-test. * p<0.05, and *** p<0.001 compared with *Bmal1*$^{+/+}$*Apoe*$^{-/-}$. Error bars represent SD.

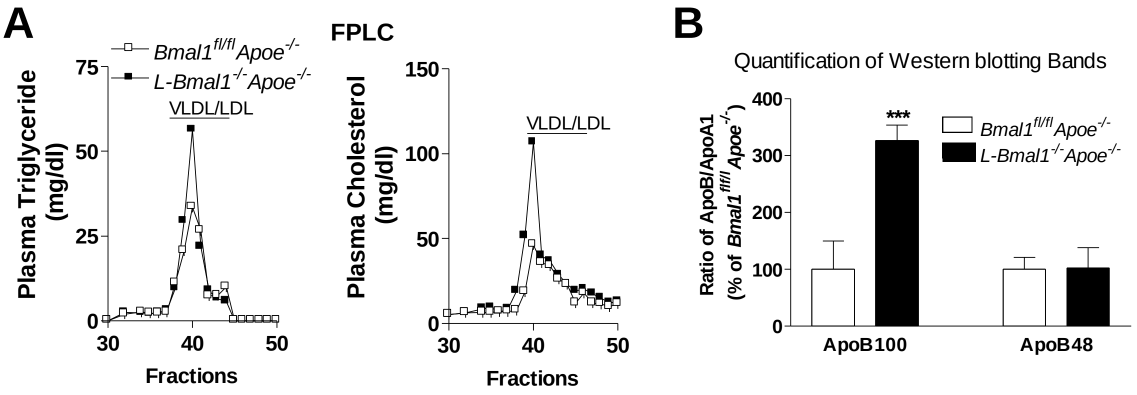

Supplementary Fig 8: Effect of liver specific Bmal1 ablation in *Apoe*$^{-/-}$ mice on plasma lipoproteins and cholesterol excretion to bile acid acceptors in hepatocytes.

Bmal1$^{fl/fl}$*Apoe*$^{-/-}$ (□) and *L-Bmal1*$^{-/-}$*Apoe*$^{-/-}$ (■) male mice were fed a chow diet (n=8/group) for 8 months. (**A**) Plasma from *Bmal1*$^{fl/fl}$*Apoe*$^{-/-}$ and *L-Bmal1*$^{-/-}$*Apoe*$^{-/-}$ mice (n=8/group) was pooled and subjected to FPLC. Triglyceride and cholesterol were measured in different fractions.

(**B**) Bands corresponding to apoB100 and apoB48 and apoA1 in Fig 3D were quantified and ApoB/ApoA1 ratios were plotted. Each group represents mean ± SD, n=6/group, unpaired Student's *t*-test. *** $P < 0.001$, compared to *Bmal1*$^{fl/fl}$*Apoe*$^{-/-}$ mice (Experiments were repeated two times). Error bars represent SD.

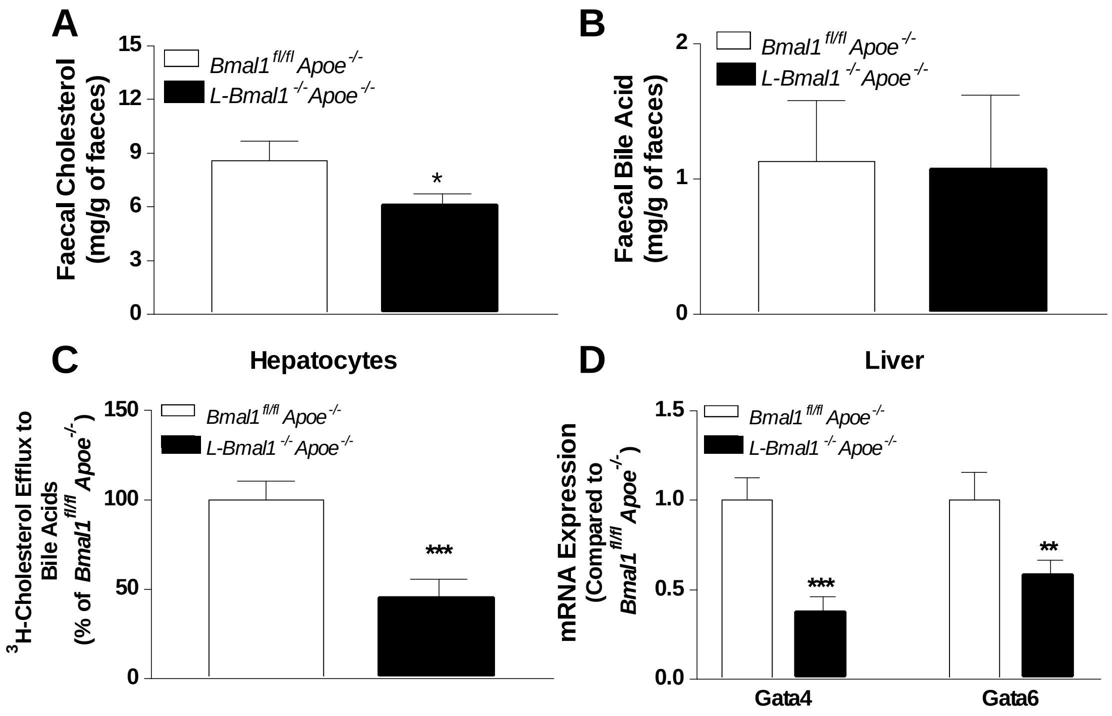

Supplementary Fig 9: Total fecal cholesterol (A) and bile acid (B) excretion (mg/g of feces weight) over 48 h in 8 month old *Bma1l*$^{fl/fl}$*Apoe*$^{-/-}$ (□) and *L-Bmal1*$^{-/-}$*Apoe*$^{-/-}$ (■) male mice were fed a chow diet.

(**C**) Primary hepatocytes from *Bmal1*$^{fl/fl}$*Apoe*$^{-/-}$ and *L-Bmal1*$^{-/-}$*Apoe*$^{-/-}$ mice were incubated for 16 hour with ^{3}H-cholesterol (1 µCi/mL). Cells were washed and amounts of cholesterol effluxed to 100 µM TUDC bile acid acceptors in 4 h were plotted.

(**D**) Hepatic mRNA levels of Gata4 and Gata6 were quantified.

Values are mean ± SD, n=6/group, unpaired Student's *t*-test. * $p<0.05$, ** $p<0.01$, and *** $p<0.001$ compared with *Bmal1*$^{fl/fl}$*Apoe*$^{-/-}$. Error bars represent SD.

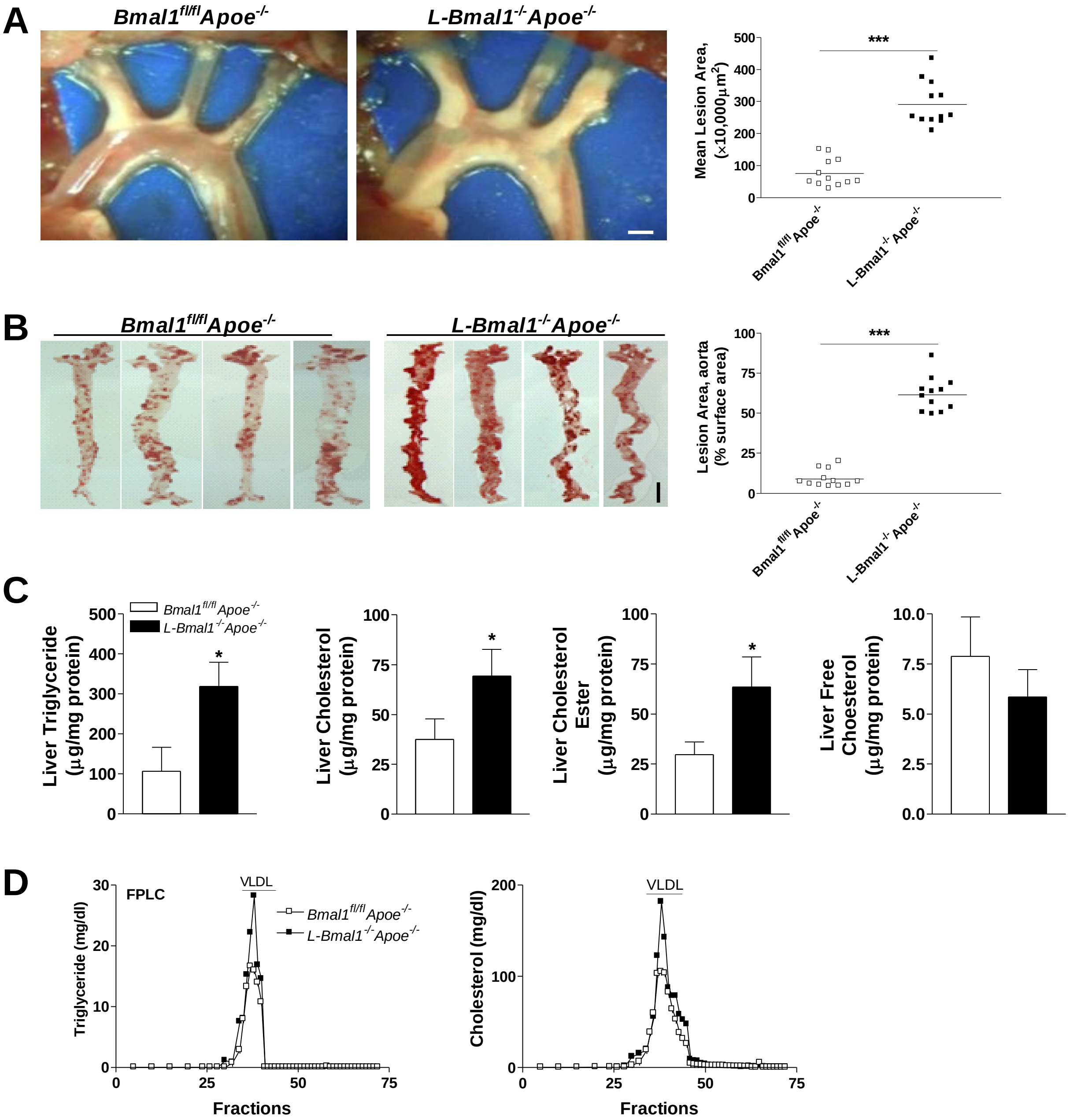

Supplementary Fig 10: Atherosclerosis in western diet fed *Bmal1$^{fl/fl}$Apoe$^{-/-}$* and *L-Bmal1$^{-/-}$Apoe$^{-/-}$* mice.

(**A-B**) Male mice (6 months,) were fed a Western diet for 2 months and plaques were photographed and quantified (A) Scale bar, 2 mm. Aortas from these mice were collected, Oil Red O stained, photographed and quantified (B) Scale bar, 5.0 mm. Values are mean ± SD, n=12/group, one-way ANOVA. *P<0.05, **P<0.01, and ***P<0.001 vs *Bmal1$^{fl/fl}$Apoe$^{-/-}$*. Error bars represent SD.

(**C**) Hepatic lipid content in these mice. Values are mean ± SD, n=12/group, unpaired Student's *t*-test. *P<0.05, **P<0.01, and ***P<0.001 vs *Bmal1$^{fl/fl}$Apoe$^{-/-}$*. Error bars represent SD.

(D) Pooled plasma samples (n=12) were subjected to FPLC.

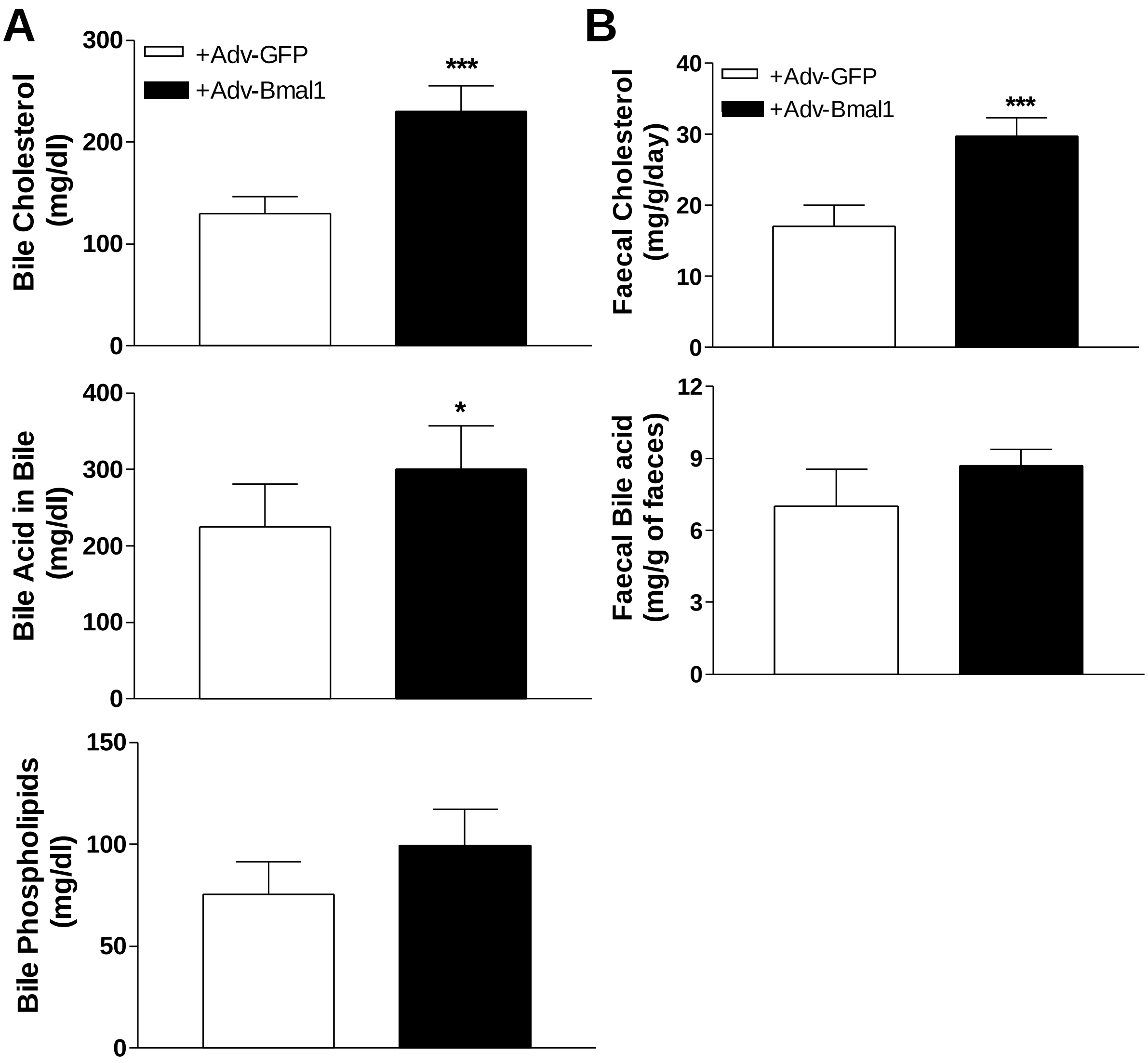

Supplementary Fig 11: Over expression of Bmal1 increase biliary cholesterol and bile acids, and fecal cholesterol in *L-Bmal1*$^{-/-}$*Apoe*$^{-/-}$ mice.

L-Bmal1$^{-/-}$*Apoe*$^{-/-}$ mice (3 months, male) were transduced with 1.5 x 10^{11} virus particles of either Adv-BMAL1 (■) or Adv-GFP (☐) and started on a Western diet. After 4 weeks, Bile and feces were collected for analysis.

(**A**) Total cholesterol, phospholipid, bile acid concentration were measured in bile of male *L-Bmal1*$^{-/-}$*Apoe*$^{-/-}$ mice transduced with Adv-GFP (☐) and Adv-Bmal1 (■).

(**B**) Total fecal cholesterol and bile acid (mg/g of feces weight) in *L-Bmal1*$^{-/-}$*Apoe*$^{-/-}$ male mice transduced with Adv-GFP (☐) and Adv-Bmal1 (■).

Values are mean ± SD, n=8/group, unpaired Student's *t*-test. * p<0.05, ** p<0.01, and *** p<0.001 compared with GFP. Error bars represent SD.

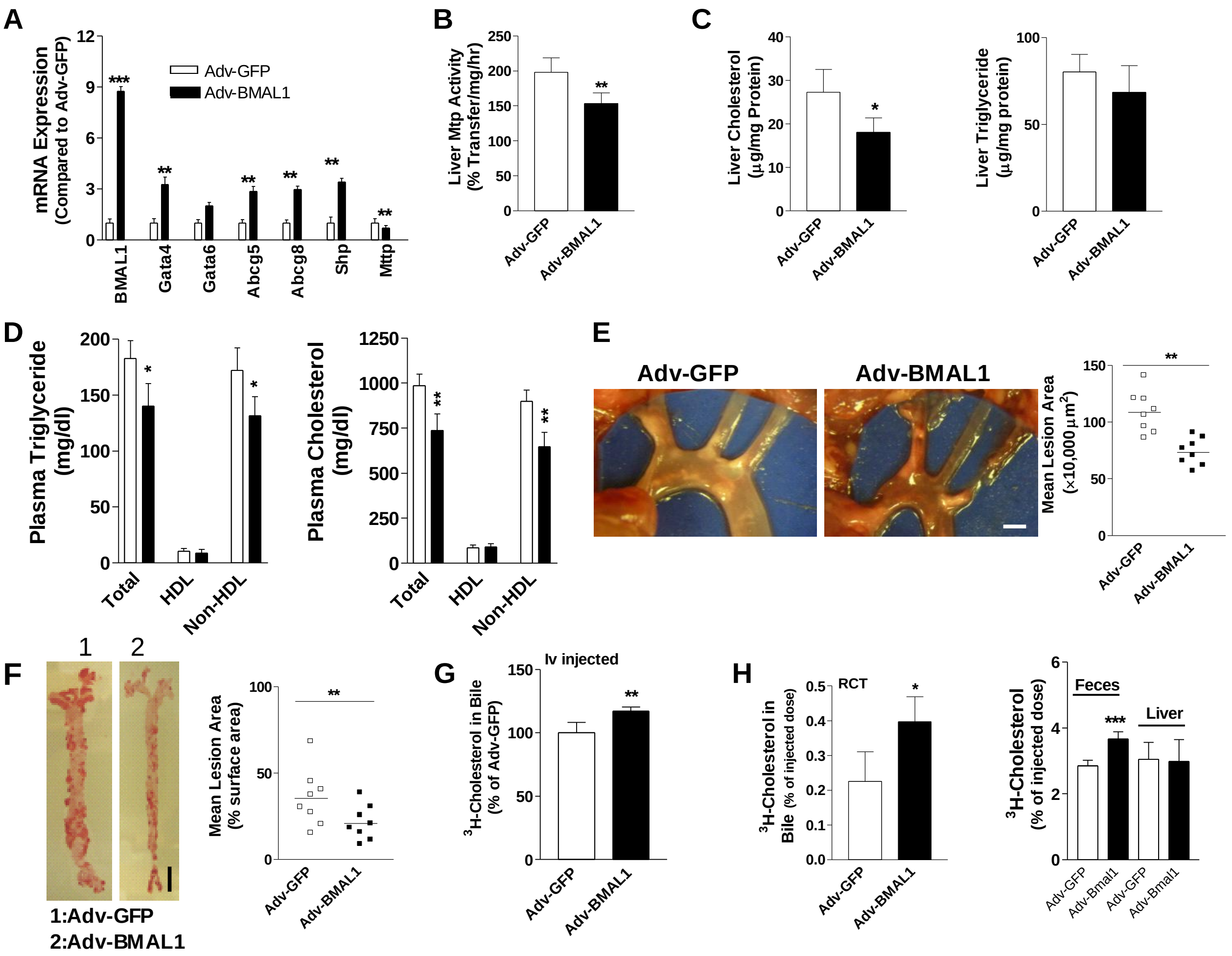

Supplementary Fig 12: Over expression of Bmal1 lowers plasma lipids, hepatic lipids and atherosclerotic lesions, and increases cholesterol excretion to bile in *Bmal1*$^{fl/fl}$*Apoe*$^{-/-}$ mice.

Male mice (*Bmal1*$^{fl/fl}$*Apoe*$^{-/-}$ mice (5 months, male) were transduced with 1.5 x 10^{11} virus particles of either Adv-BMAL1 (■) or Adv-GFP (☐) and started on a Western diet. After 4 weeks, plasma and tissues were collected for analysis. **(A)** mRNA levels of different indicated genes were quantified.

(B-C) Hepatic MTP activity (B), cholesterol and triglyceride (C) were measured.

(D) Plasma triglyceride and cholesterol were measured in total plasma and different lipoprotein fractions after separation by precipitation.

(E) Aortic arches were dissected, photographed (left) and quantified (right). Scale bar, 2 mm.

(F) Aortas were stained with Oil Red O (left) and quantified (right). Scale bar, 5.0 mm.

(G) Amounts of cholesterol excreted to bile after intravenous injection (n=4/group).

(H) Amounts of cholesterol in the bile, feces and liver 48 h after placing ^{3}H-cholestreol loaded J774 macrophages in the peritoneal cavity of different mice (n=4/group).

Values (A-D, G-H) are mean ± SD, n=8/group, unpaired Student's *t*-test. **P*<0.05, ***P*<0.01, and ****P*<0.001. Values (E-F) are mean ± SD, n=8/group, one-way ANOVA. **P*<0.05, ***P*<0.01, and ****P*<0.001. Error bars represent SD.

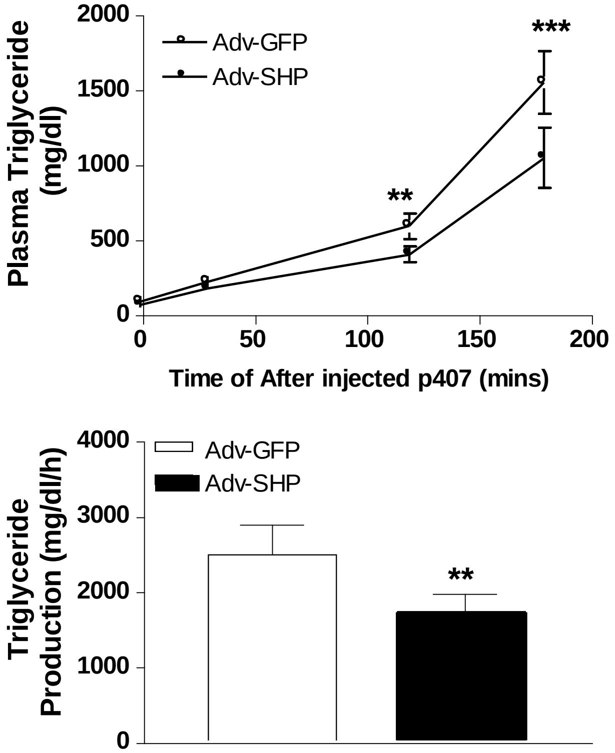

Supplementary Fig 13: Hepatic overexpression of Shp improves hepatic lipoprotein production in *L-Bmal1*$^{-/-}$*Apoe*$^{-/-}$ mice.

L-Bmal1$^{-/-}$*Apoe*$^{-/-}$ mice (male, 3 months,) were injected (1 x 10^{11} pfu per mouse) with adenoviruses expressing SHP (Adv-SHP,■) or GFP (Adv-GFP,□) and fed a Western diet for 4 weeks. Overnight fasted animals were injected with P407 and plasma lipids were determined at indicated times. There were more time-dependent decreases in plasma triglycerides of *L-Bmal1*$^{-/-}$*Apoe*$^{-/-}$ mice with Adv-SHP than *L-Bmal1*$^{-/-}$*Apoe*$^{-/-}$ mice with Adv-GFP mice (top). The triglyceride production rates were lower in *L-Bmal1*$^{-/-}$*Apoe*$^{-/-}$ mice with Adv-SHP than *L-Bmal1*$^{-/-}$*Apoe*$^{-/-}$ mice with Adv-GFP (bottom). Values are mean ± SD, n=4/group, unpaired Student's *t*-test. ***P*<0.01, and ****P*<0.001. Error bars represent SD.

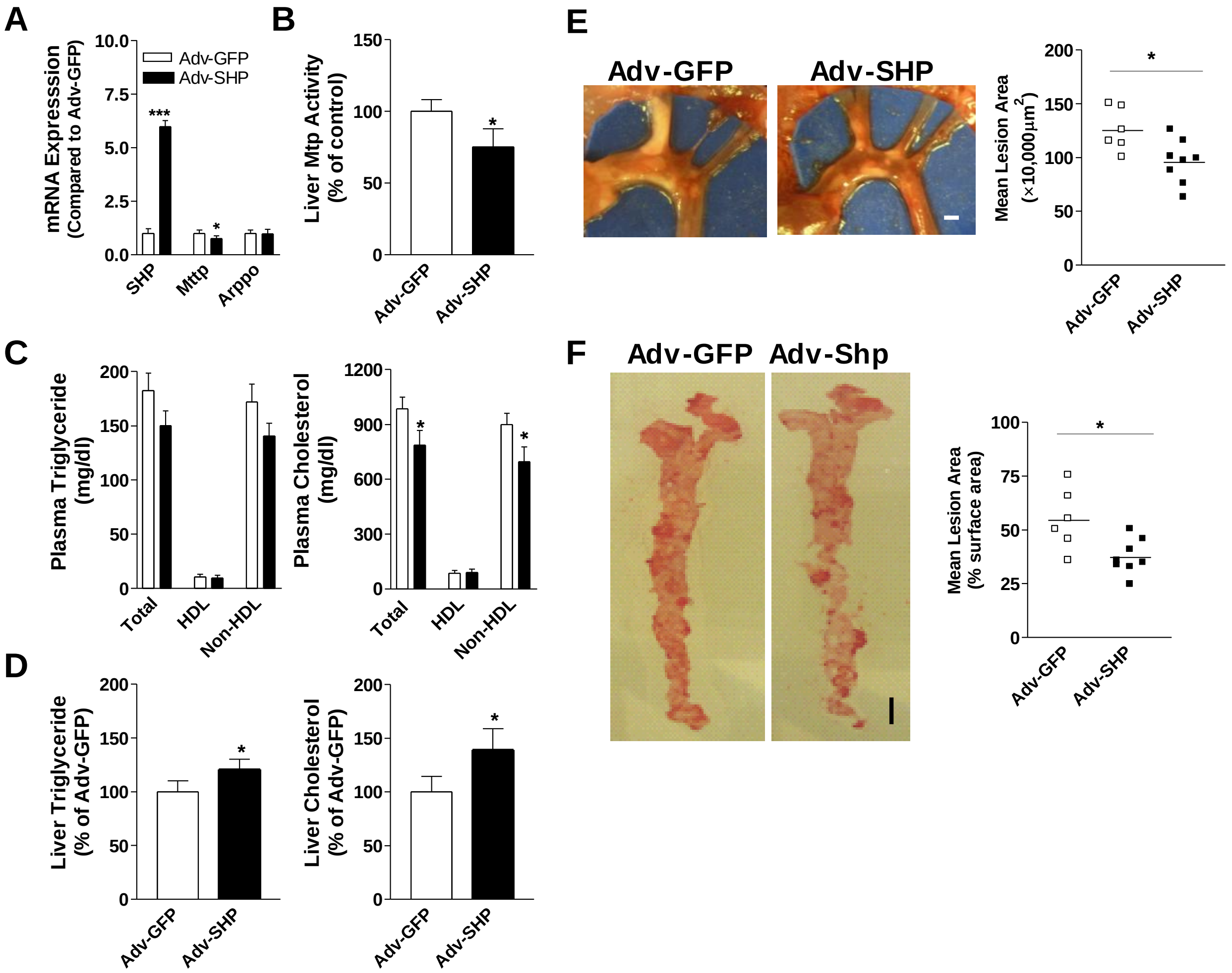

Supplementary Fig 14: Hepatic overexpression of Shp improves atherosclerosis, decreases plasma cholesterol and increases liver lipids in *Bmal*$^{fl/fl}$*Apoe*$^{-/-}$ mice:

Bmal1$^{fl/fl}$*Apoe*$^{-/-}$ mice (male, 4 months,) were injected (1x 10^{11} pfu per mouse) with adenoviruses expressing SHP (Adv-SHP,■) or GFP (Adv-GFP,□) and fed a Western diet for 4 weeks. **(A)** Hepatic mRNA levels of different genes.

(B) Livers from these mice were used to measure MTP activity.

(C) Triglyceride and cholesterol were measured in total plasma and different lipoprotein fractions.

(D) Lipids were measured in the livers.

(E) The aortic arches were exposed, photographed (left) and lesion areas were quantified (right) with Image-Pro. Scale bar, 2 mm.

(F) Aortas were dissected, stained with Oil Red O (left), and quantified (right). Scale bar,5.0 mm.

Values (A-D) are mean ± SD, n=6/group, unpaired Student's *t*-test. *P<0.05 and ***P<0.001. Values (E-F) are mean ± SD, n=6-8/group, one-way ANOVA. *P<0.05. Error bars represent SD.

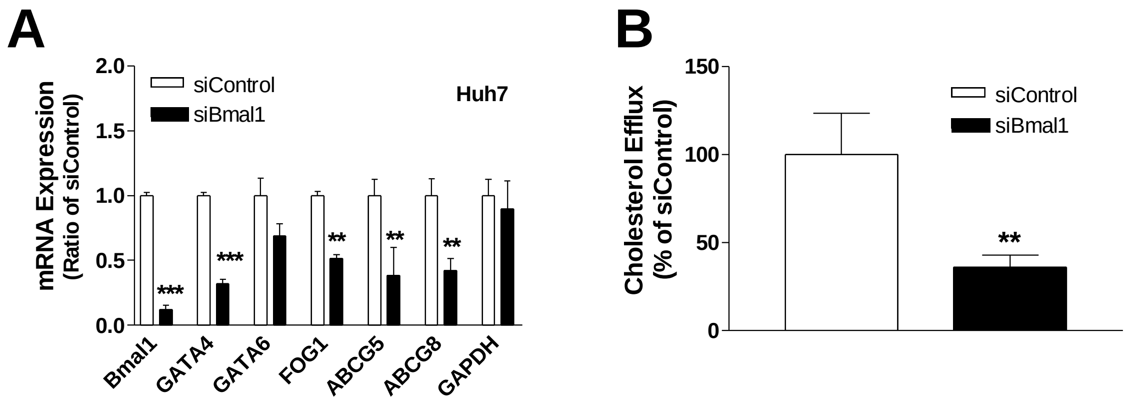

Supplementary Fig 15: Effect of Bmal1 knockdown on gene expression and cholesterol efflux to bile acid acceptors in Huh-7 cells.

(**A**) Huh-7 cells were transfected with siControl or siBmal1. After 48 h, mRNA levels of different genes were quantified.

(**B**) These cells were loaded with cholesterol and then used for cholesterol efflux to bile acid acceptors. Values are mean ± SD, n=4, unpaired Student's *t*-test. ***P*<0.01, and ****P*<0.001 vs siControl group. Error bars represent SD.

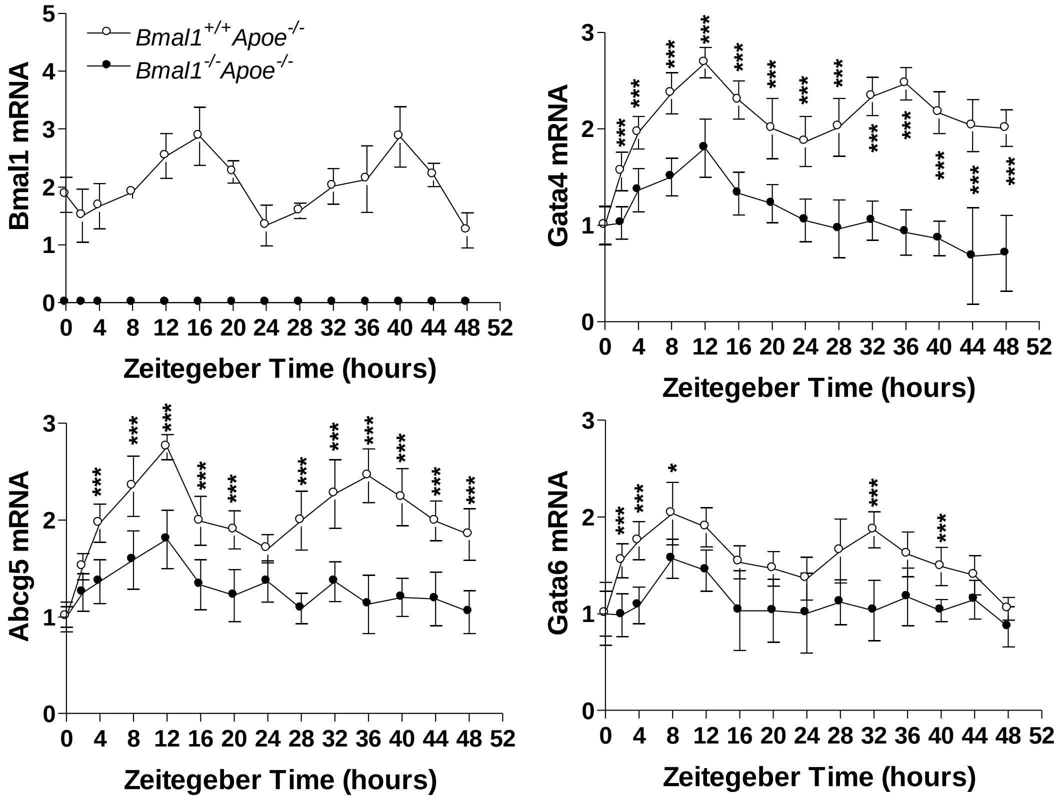

Supplementary Fig 16: Cyclic expression of gene expression in *Bmal1*$^{-/-}$*Apoe*$^{-/-}$ and *Bmal1*$^{+/+}$*Apoe*$^{-/-}$ mice.

Hepatocytes from *Bmal1*$^{-/-}$*Apoe*$^{-/-}$ and *Bmal1*$^{+/+}$*Apoe*$^{-/-}$ mice (3 months, male) were cultured and subjected to serum shock and changes in mRNA levels of indicated genes were quantified at indicated times. Values are mean ± SD, n=3, two-way ANOVA. **P*<0.05, ***P*<0.01, and ****P*<0.001 vs siControl group. Error bars represent SD.

A

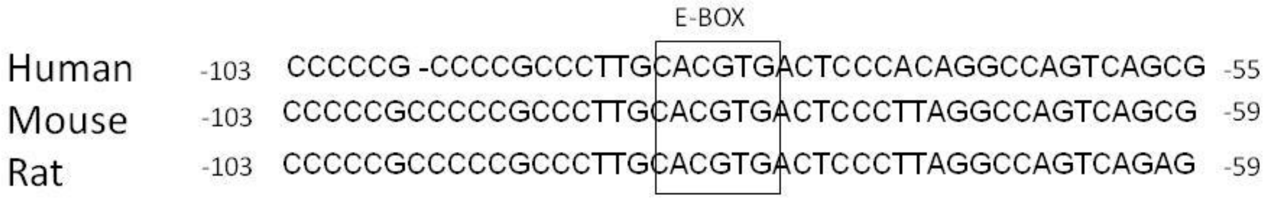

B

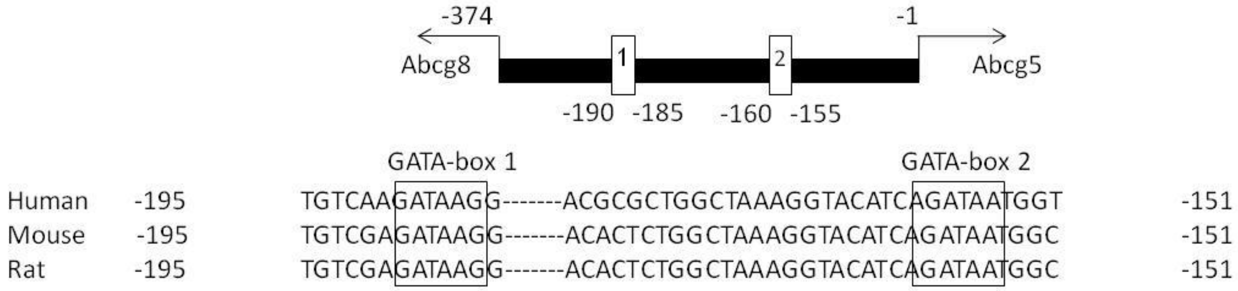

Supplementary Fig 17: E- and GATA-boxes in the promoters of *Gata4* and *Abcg5/Abcg8*.

(A) Nucleotide sequence comparison of the 5´-upstream regions of mammalian Gata4 genes showing the presence of a conserved E-box (CACGTG, from -85 to -80).

(B) Nucleotide sequence of the human, rat and mouse of Abcg5 and Abcg8 promoters. There are two GATA sites conserved in these promoters. For ChIP GATA-Box 2 was amplified.

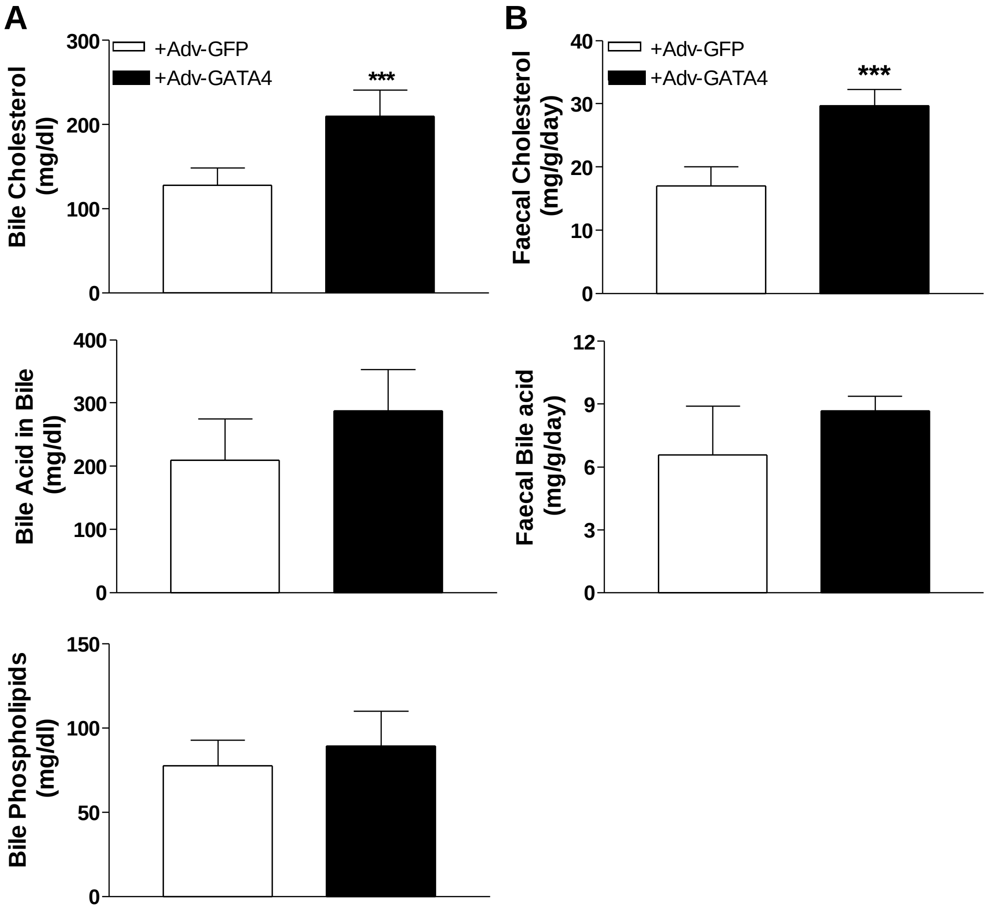

Supplementary Fig 18: Overexpression of GATA4 increases bile and fecal cholesterol.

L-Bmal1$^{-/-}$*Apoe*$^{-/-}$ mice were transduced with (1.5 x 10^{11} pfu) Adv-GFP (□) or Adv-GATA4 (■) and started on a Western diet. After 4 weeks bile was collected from gall bladder. Feces collected over 48 h were used for different analysis. (A) Biliary total cholesterol, phospholipid and bile acids

(B) Total fecal cholesterol and bile acid (mg/g of feces).

Values are mean ± SD, n=8/group, unpaired Student's *t*-test. * p<0.05, ** p<0.01, and *** p<0.001 compared with Adv-GFP groups mice. Error bars represent SD.

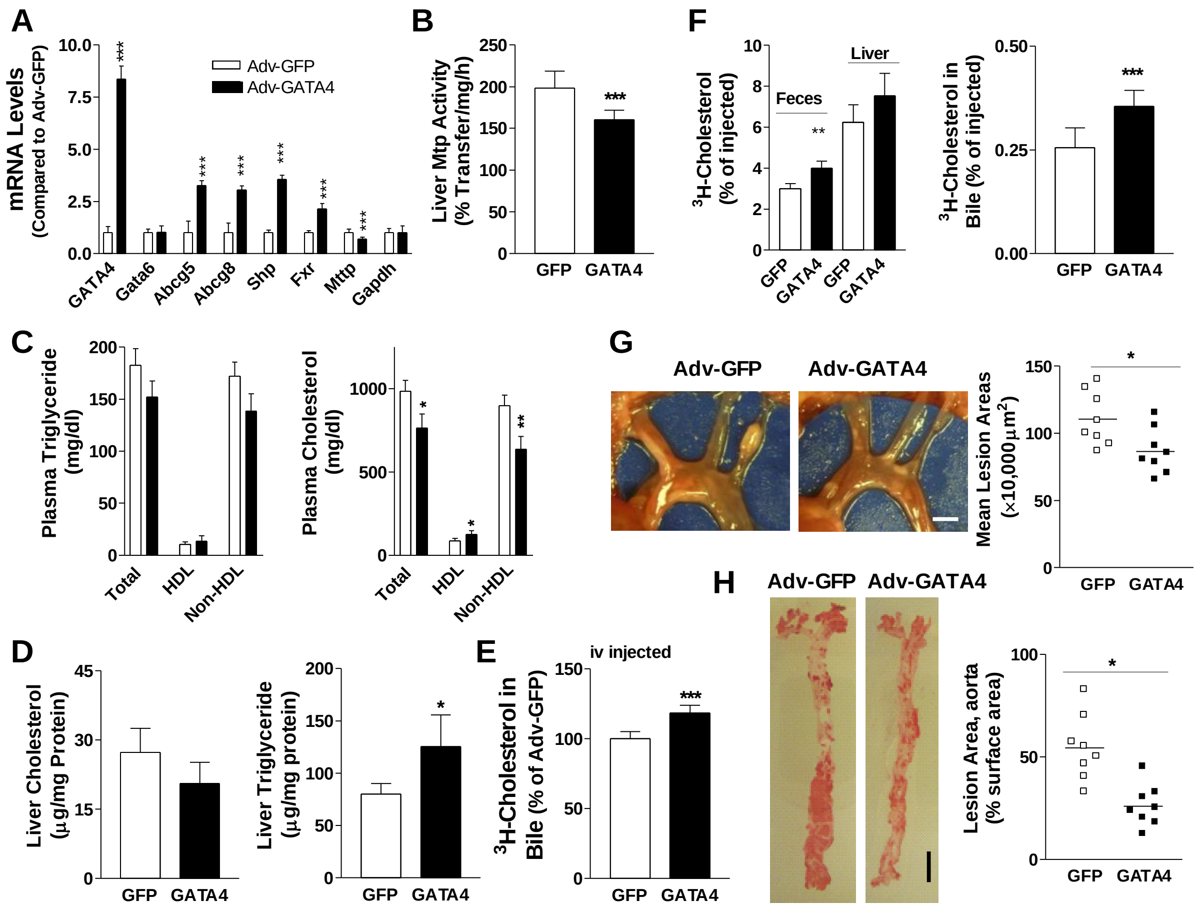

Supplementary Fig 19: Overexpression of GATA4 reduces plasma lipids and atherosclerosis in western diet fed *Bmal1*$^{fl/fl}$*Apoe*$^{-/-}$ mice.

Bmal1$^{fl/fl}$*Apoe*$^{-/-}$ mice (5 months) were transduced with (1.5 x 10^{11} pfu) Adv-GFP (☐) or Adv-GATA4 (■) and started on a Western diet. After 4 weeks plasma and liver were collected for different analysis.

(A) mRNA levels of indicated genes in the liver.

(B) Hepatic MTP activity was measured in mice transduced with different viruses.

(C) Triglyceride and cholesterol in total plasma, HDL and non-HDL.

(D) Hepatic cholesterol and triglyceride were quantified.

(E) Amount of ^{3}H-cholesterol excreted into the bile after intravenous injections (n=6/group).

(F) Amounts of cholesterol found in the bile, feces and liver 48 h after the placement of ^{3}H-cholesterol loaded macrophages in the peritoneum during RCT (n=6/group).

(G) Aortas were stained for lipids and lesion areas were quantified. Scale bar, 2 mm.

(H) Atherosclerotic plaques in the aortic branches were exposed and photographed. Scale bar, 5.0 mm.

Values (A-F) are mean ± SD, n=8/group, unpaired Student's *t*-test. **P*<0.05, ***P*<0.01, and ****P*<0.001. Values (G-H) are mean ± SD, n=8/group, one-way ANOVA. **P*<0.05, ***P*<0.01, and ****P*<0.001. Error bars represent SD.

Fig 2A

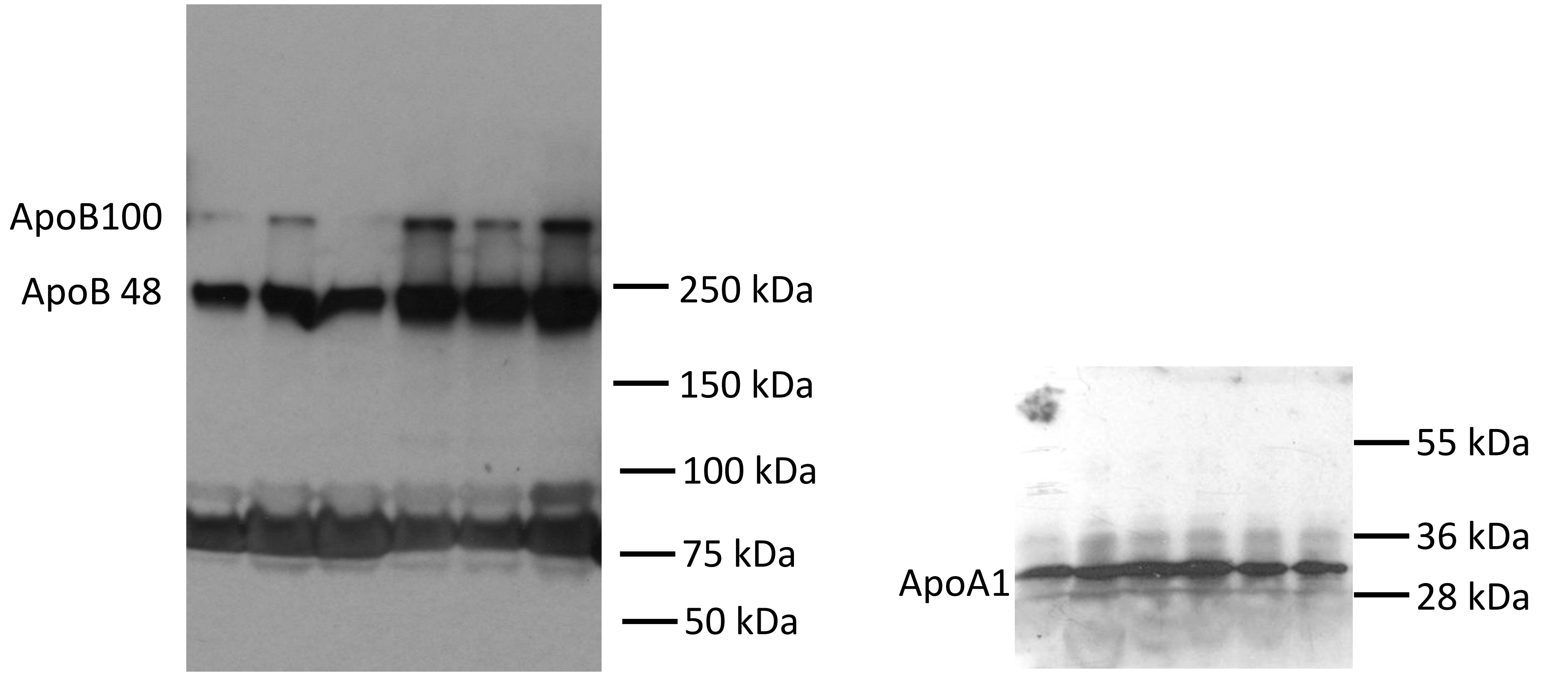
ApoB100
ApoB 48
250 kDa
150 kDa
100 kDa
75 kDa
50 kDa
ApoA1
55 kDa
36 kDa
28 kDa

Fig 2E

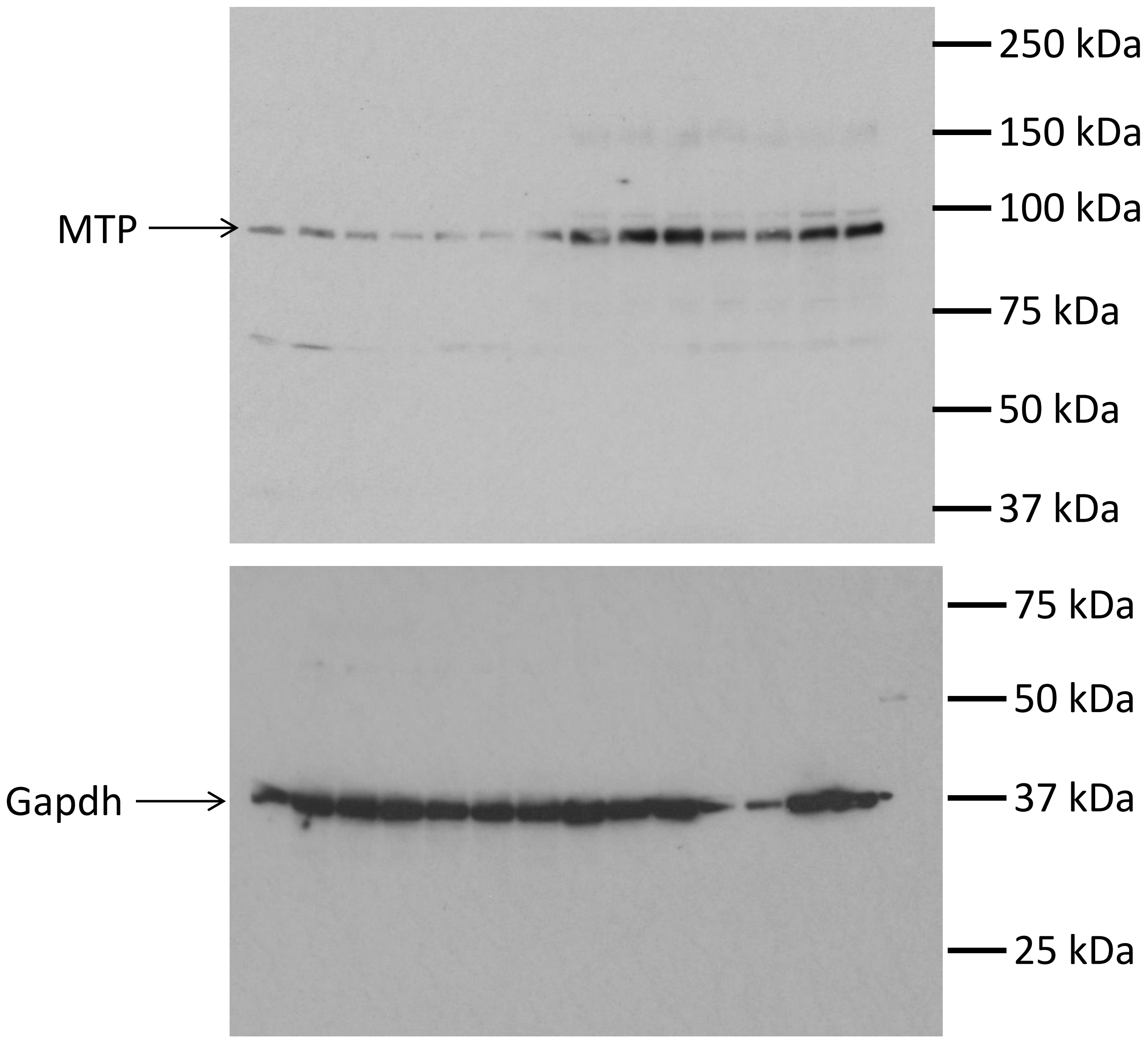
MTP
Gapdh
250 kDa
150 kDa
100 kDa
75 kDa
50 kDa
37 kDa
75 kDa
50 kDa
37 kDa
25 kDa

Fig 2G

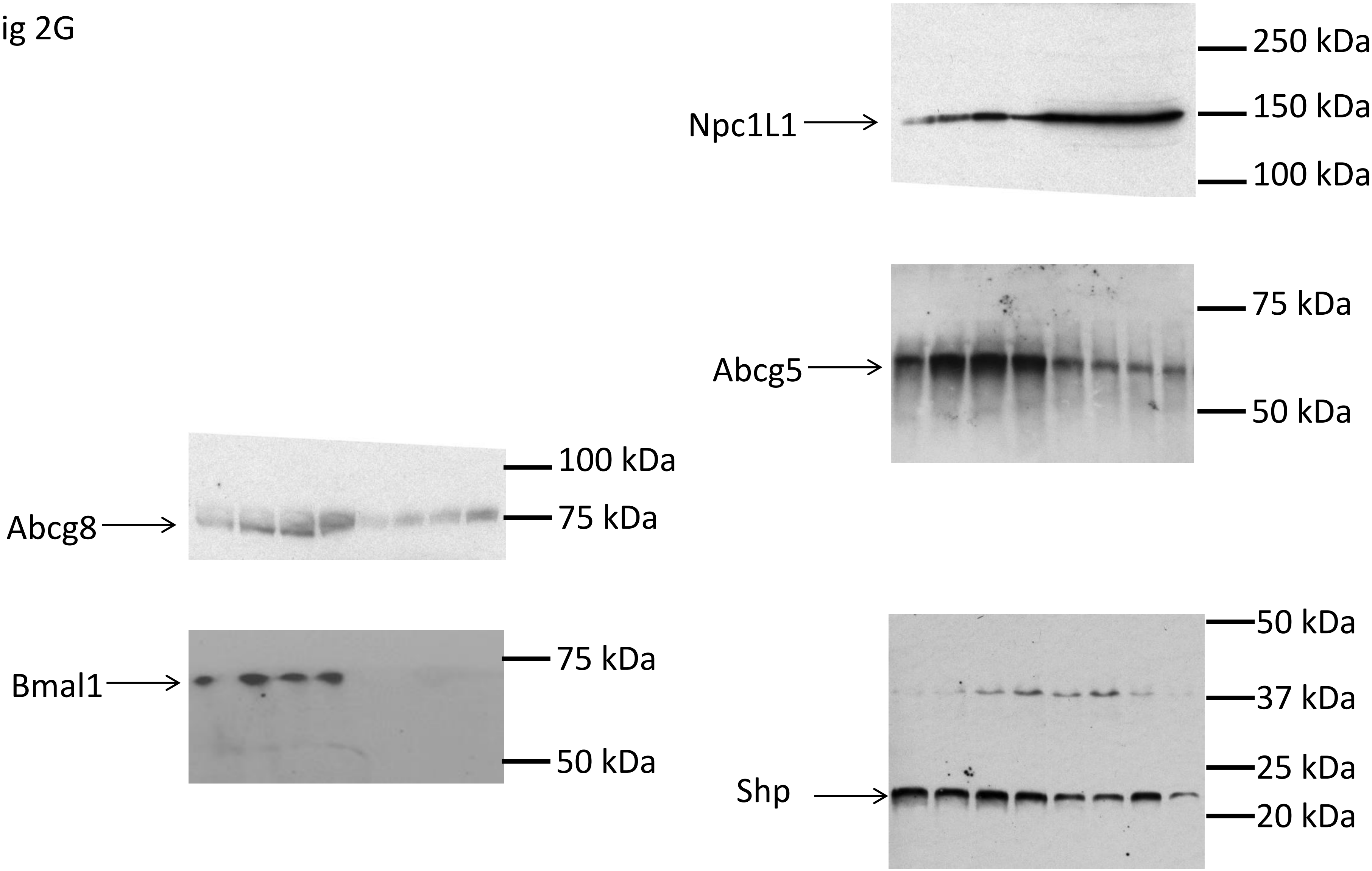
Npc1L1
250 kDa
150 kDa
100 kDa
Abcg5
75 kDa
50 kDa
Abcg8
100 kDa
75 kDa
Bmal1
75 kDa
50 kDa
Shp
50 kDa
37 kDa
25 kDa
20 kDa

Fig 2G continued

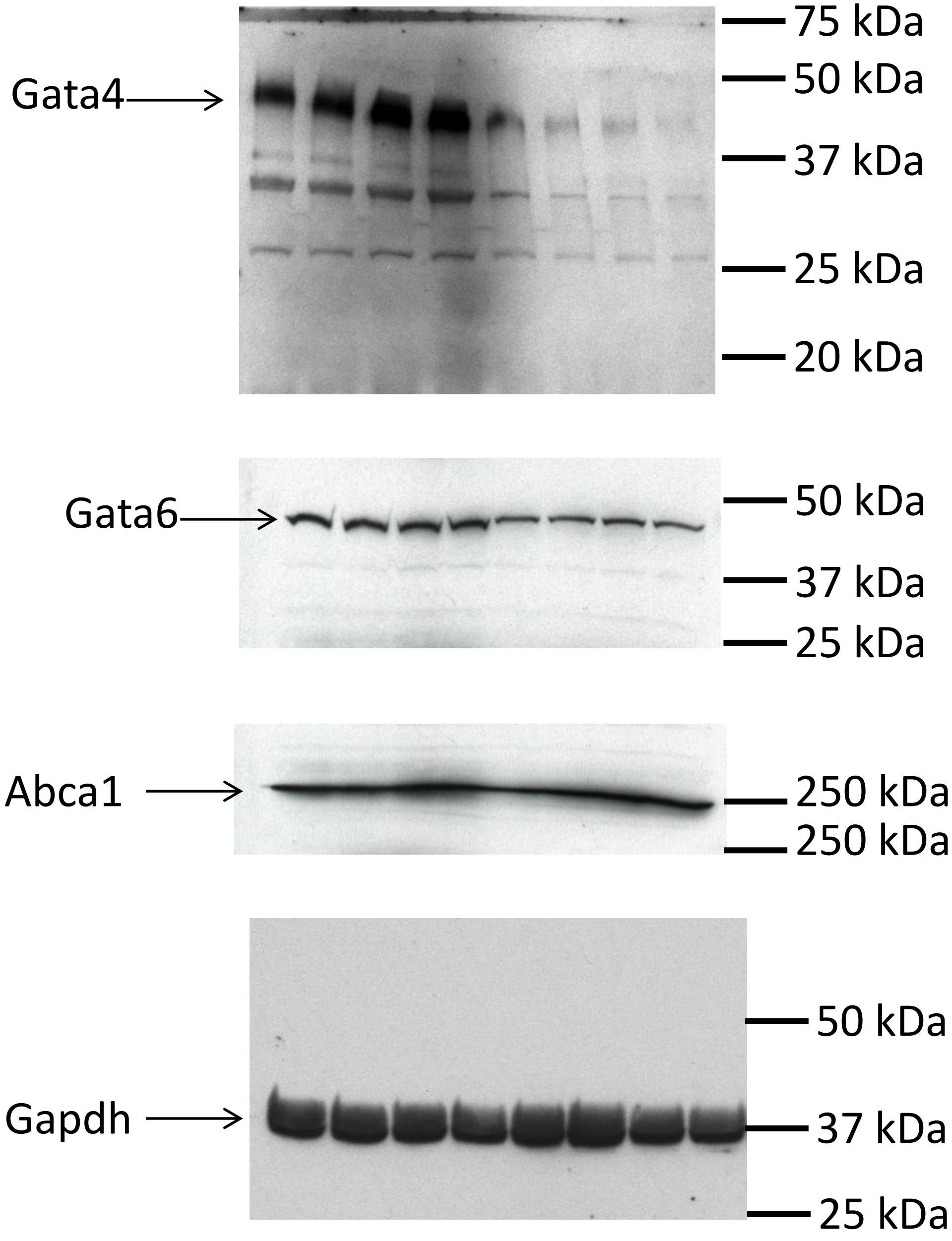

Fig 3D

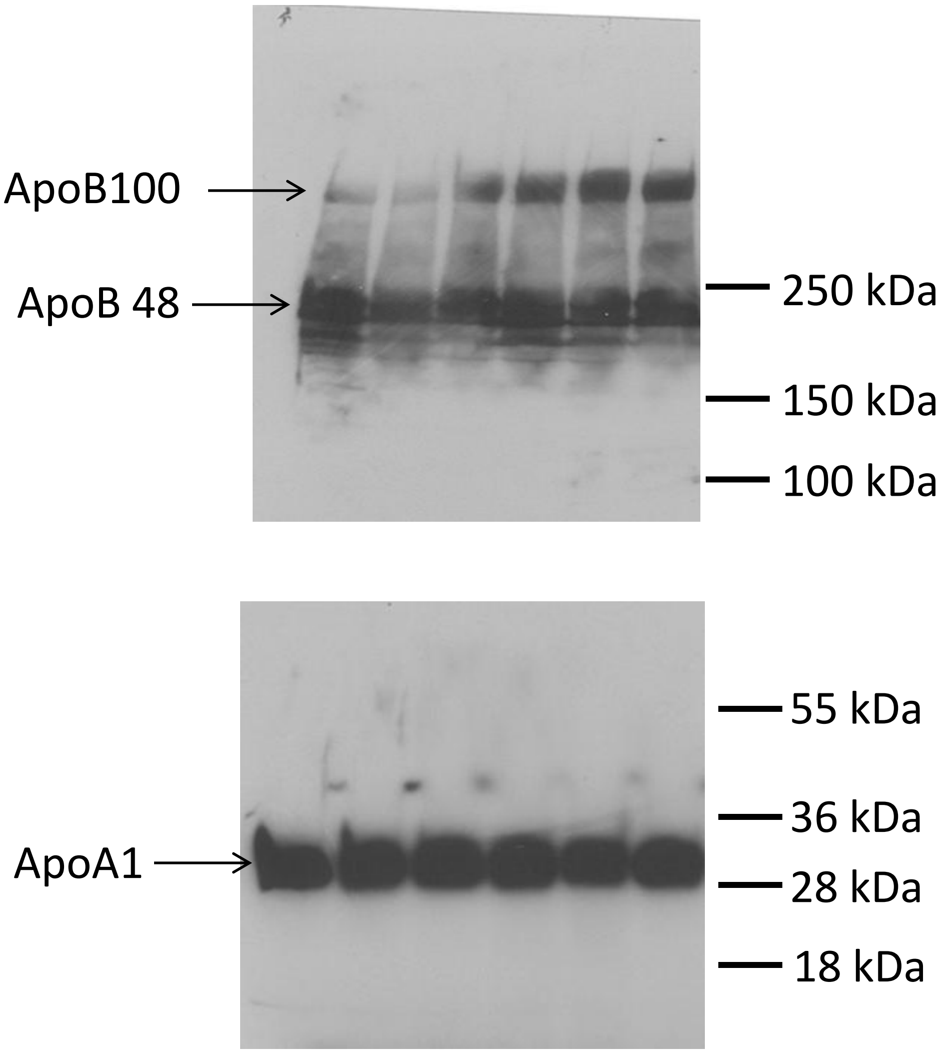

ApoB100
ApoB 48
250 kDa
150 kDa
100 kDa
55 kDa
36 kDa
ApoA1
28 kDa
18 kDa

Fig 3F

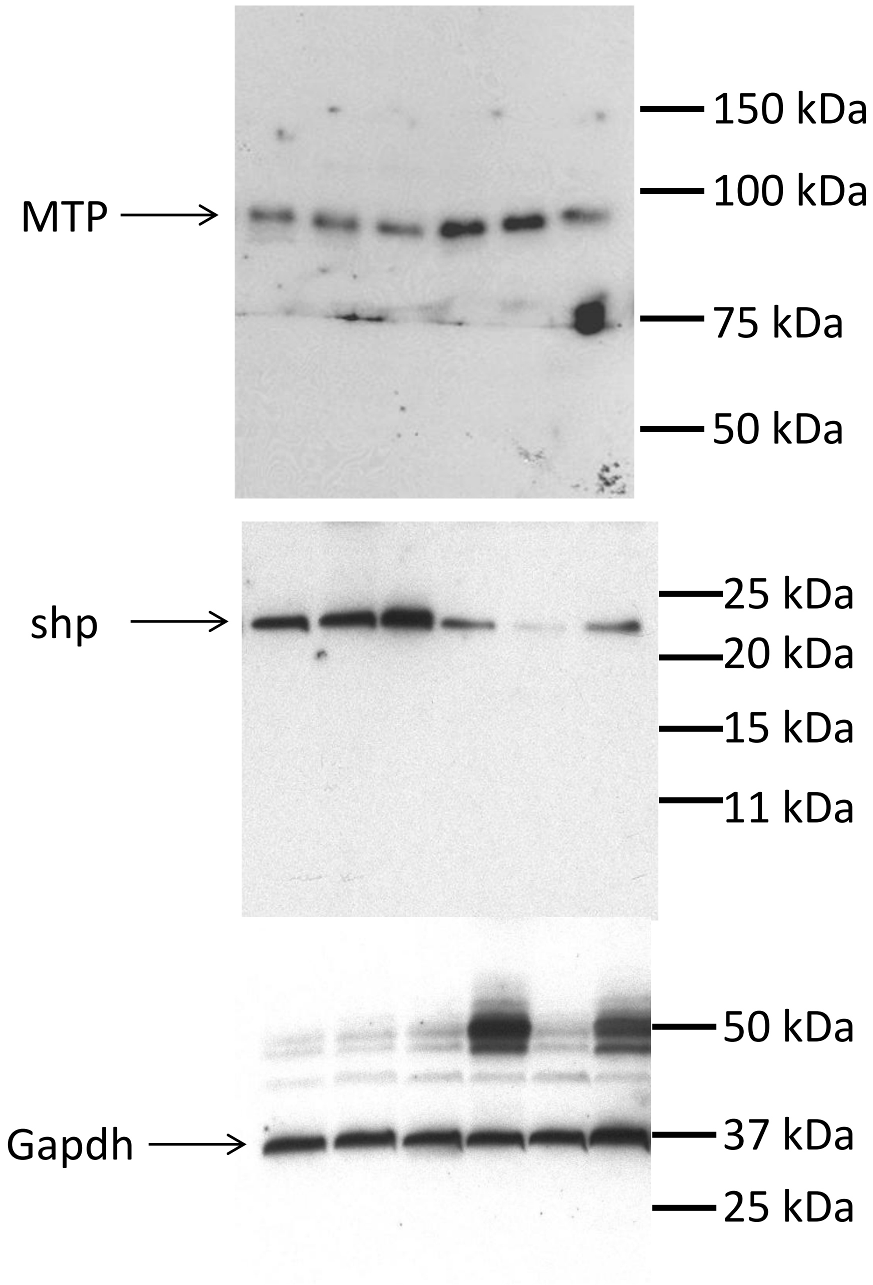
MTP
150 kDa
100 kDa
75 kDa
50 kDa
shp
25 kDa
20 kDa
15 kDa
11 kDa
Gapdh
50 kDa
37 kDa
25 kDa

Fig 3J

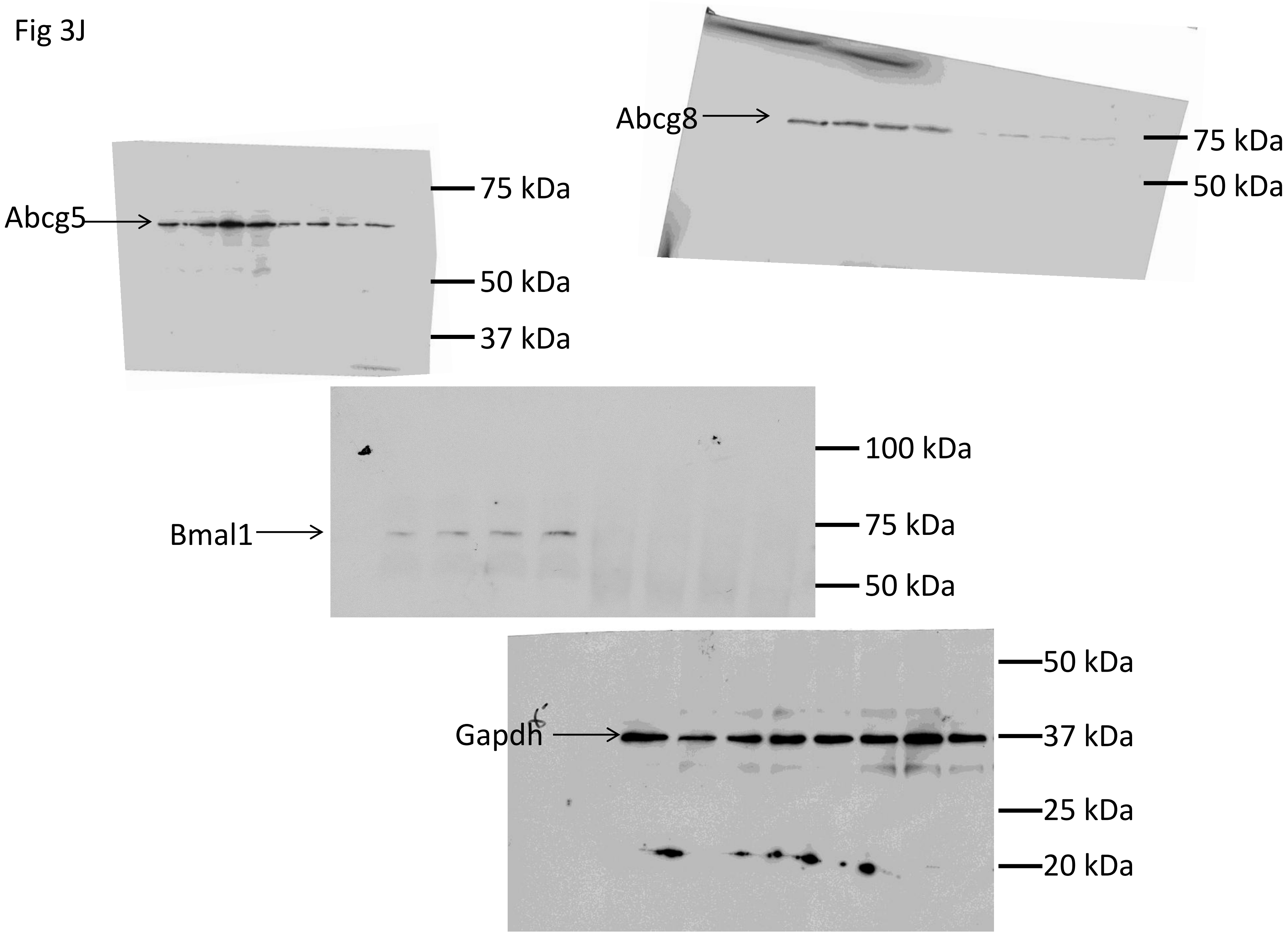
Abcg5
75 kDa
50 kDa
37 kDa
Abcg8
75 kDa
50 kDa
Bmal1
100 kDa
75 kDa
50 kDa
Gapdh
50 kDa
37 kDa
25 kDa
20 kDa

Fig 4A

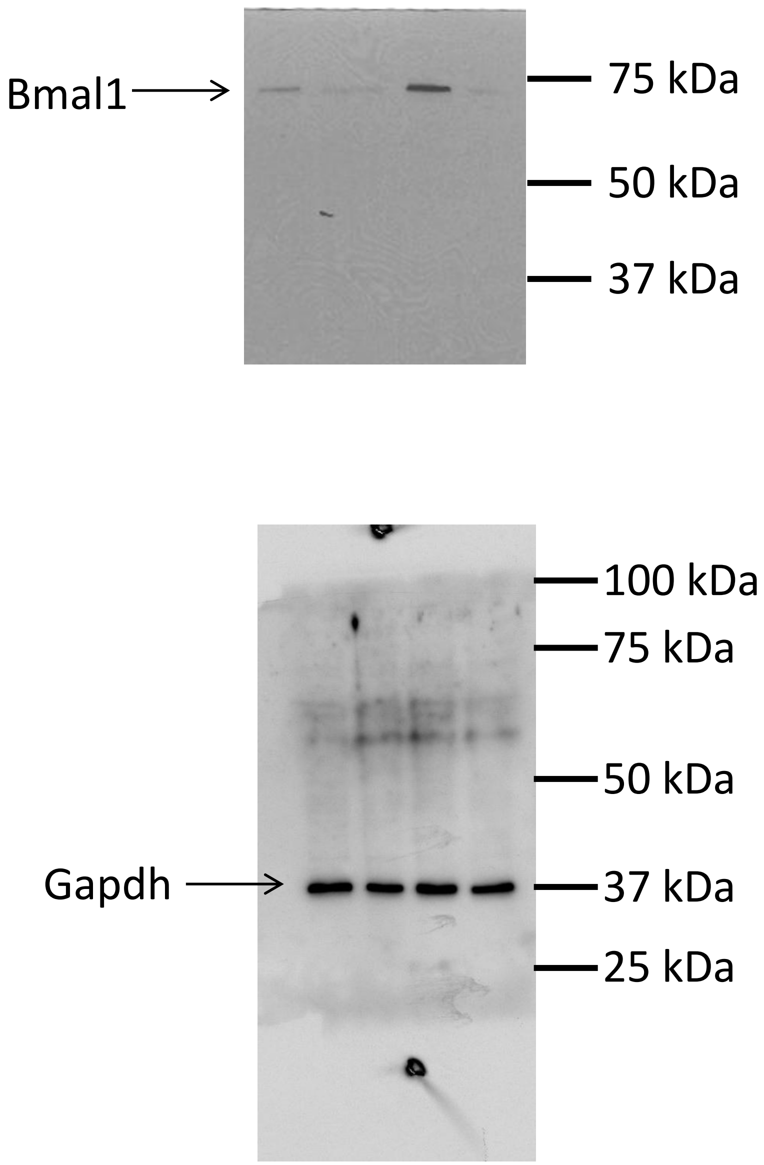
Bmal1
75 kDa
50 kDa
37 kDa
100 kDa
75 kDa
50 kDa
Gapdh
37 kDa
25 kDa

Fig 4B

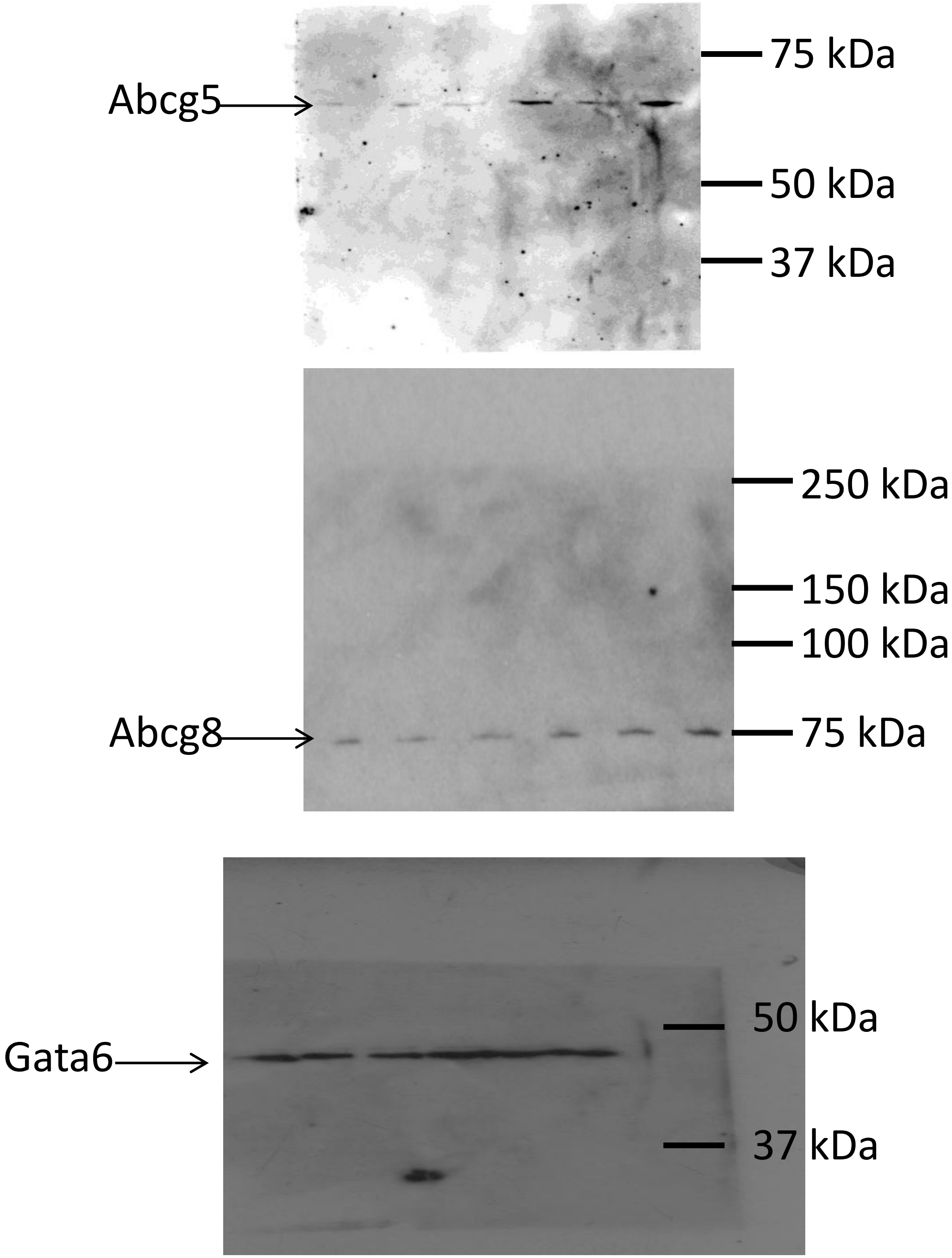
Abcg5
75 kDa
50 kDa
37 kDa
Abcg8
250 kDa
150 kDa
100 kDa
75 kDa
Gata6
50 kDa
37 kDa

Fig 4B continued

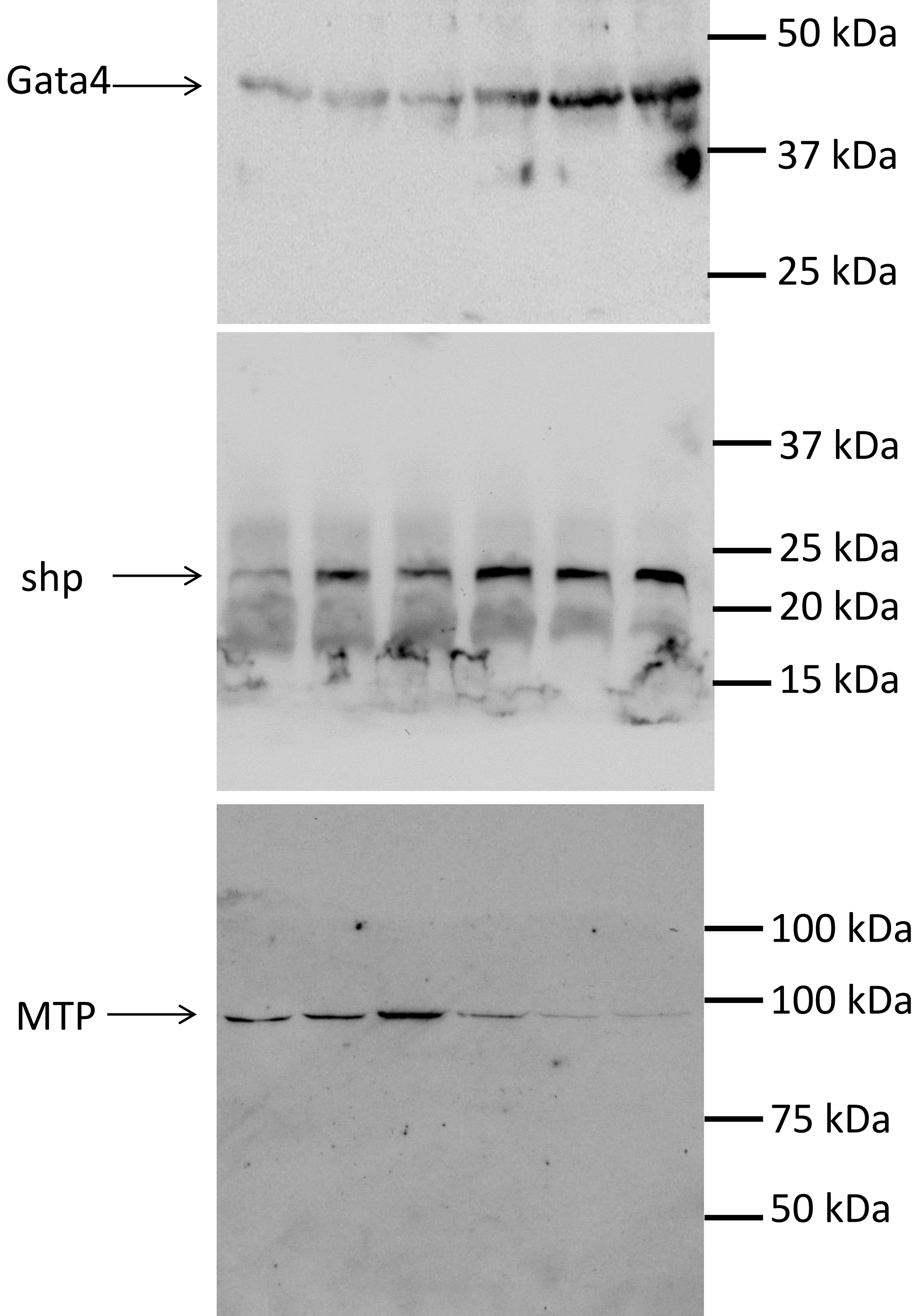

Fig 5C

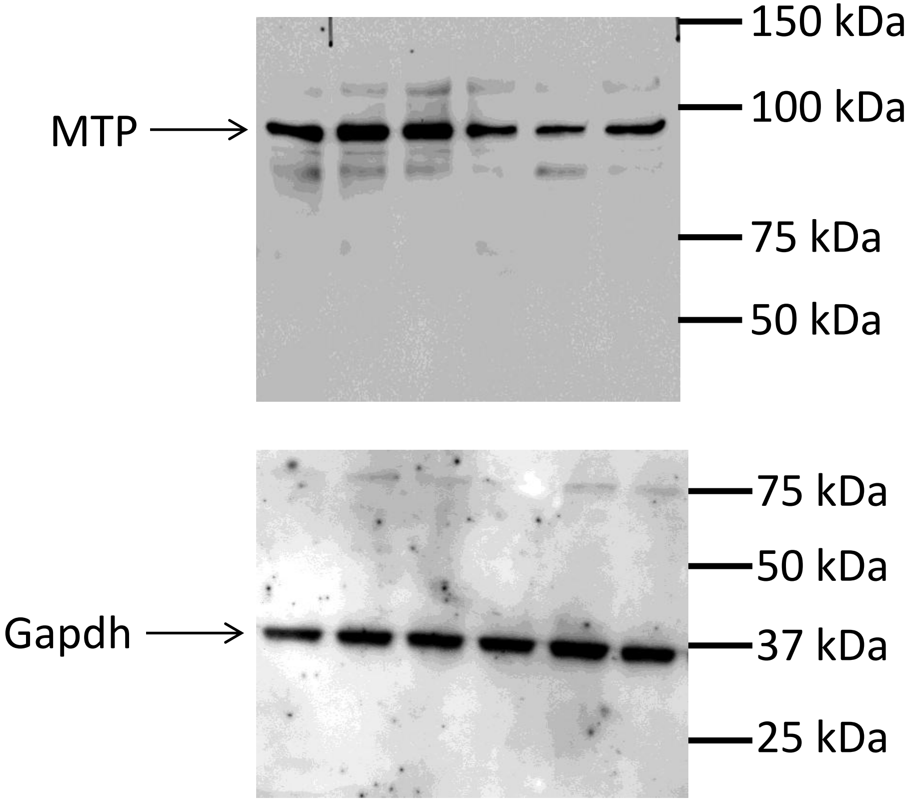
MTP
150 kDa
100 kDa
75 kDa
50 kDa
Gapdh
75 kDa
50 kDa
37 kDa
25 kDa

Fig 6A

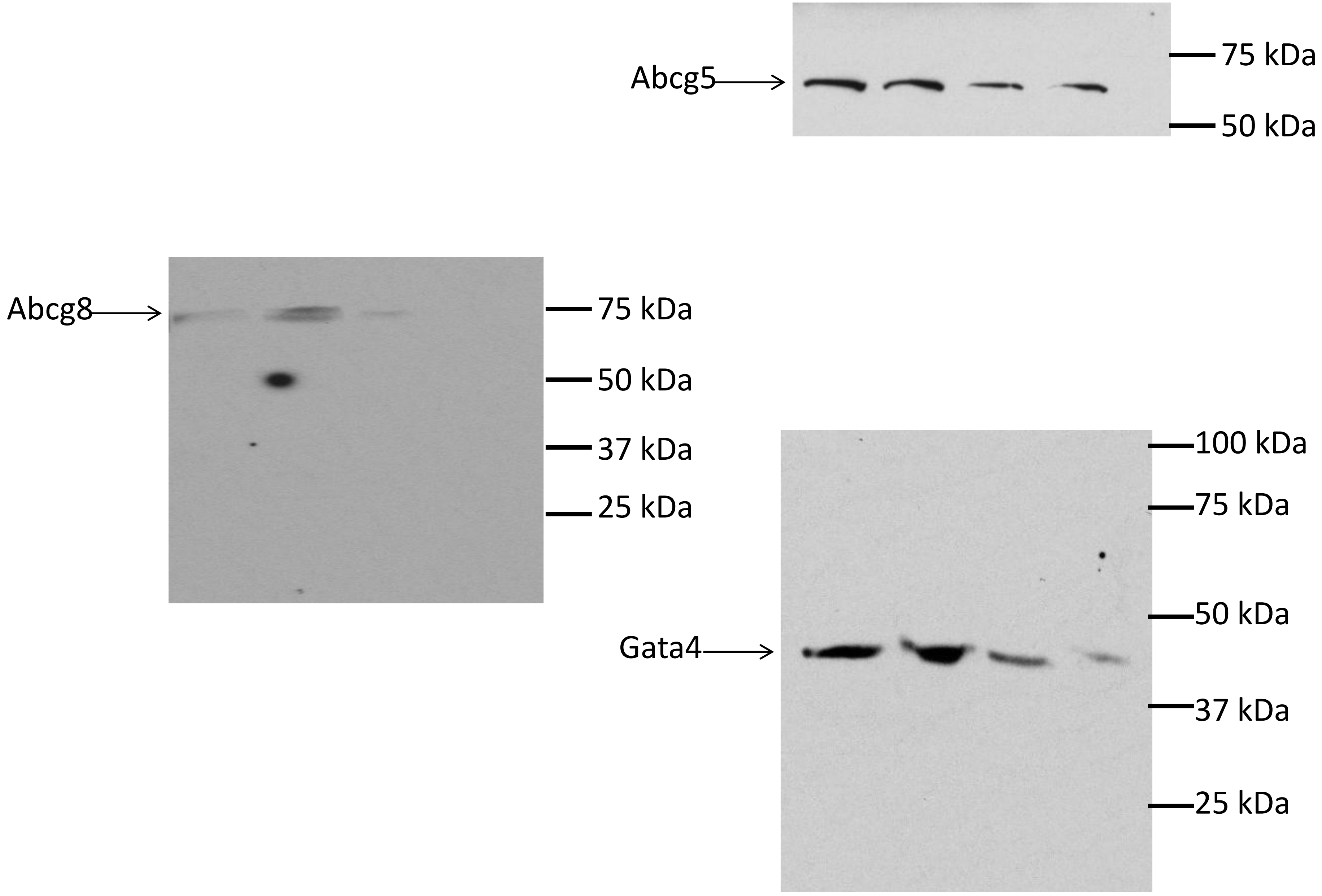
Abcg5
75 kDa
50 kDa
Abcg8
75 kDa
50 kDa
37 kDa
25 kDa
Gata4
100 kDa
75 kDa
50 kDa
37 kDa
25 kDa

Fig 6A continued

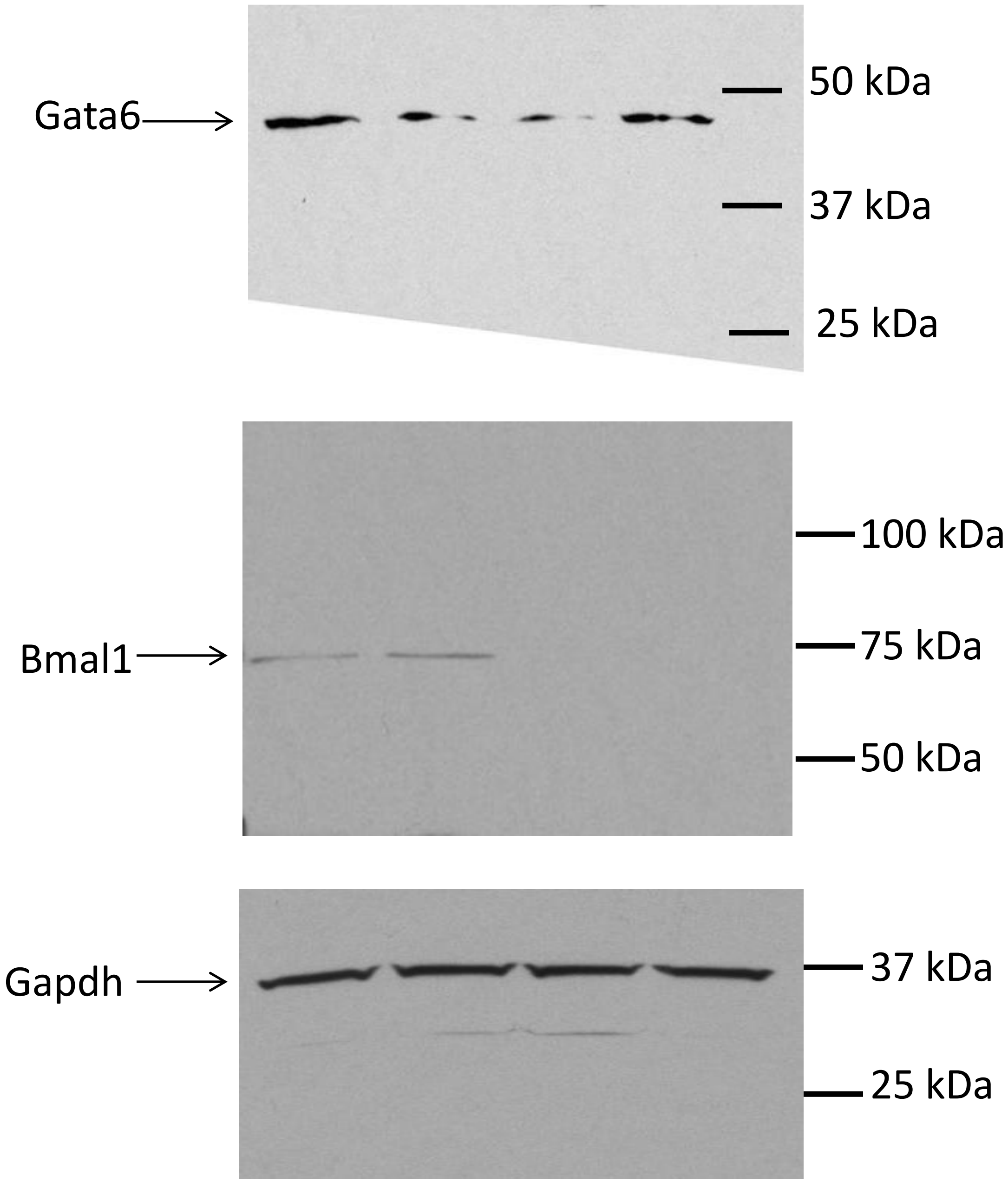

Fig 6C

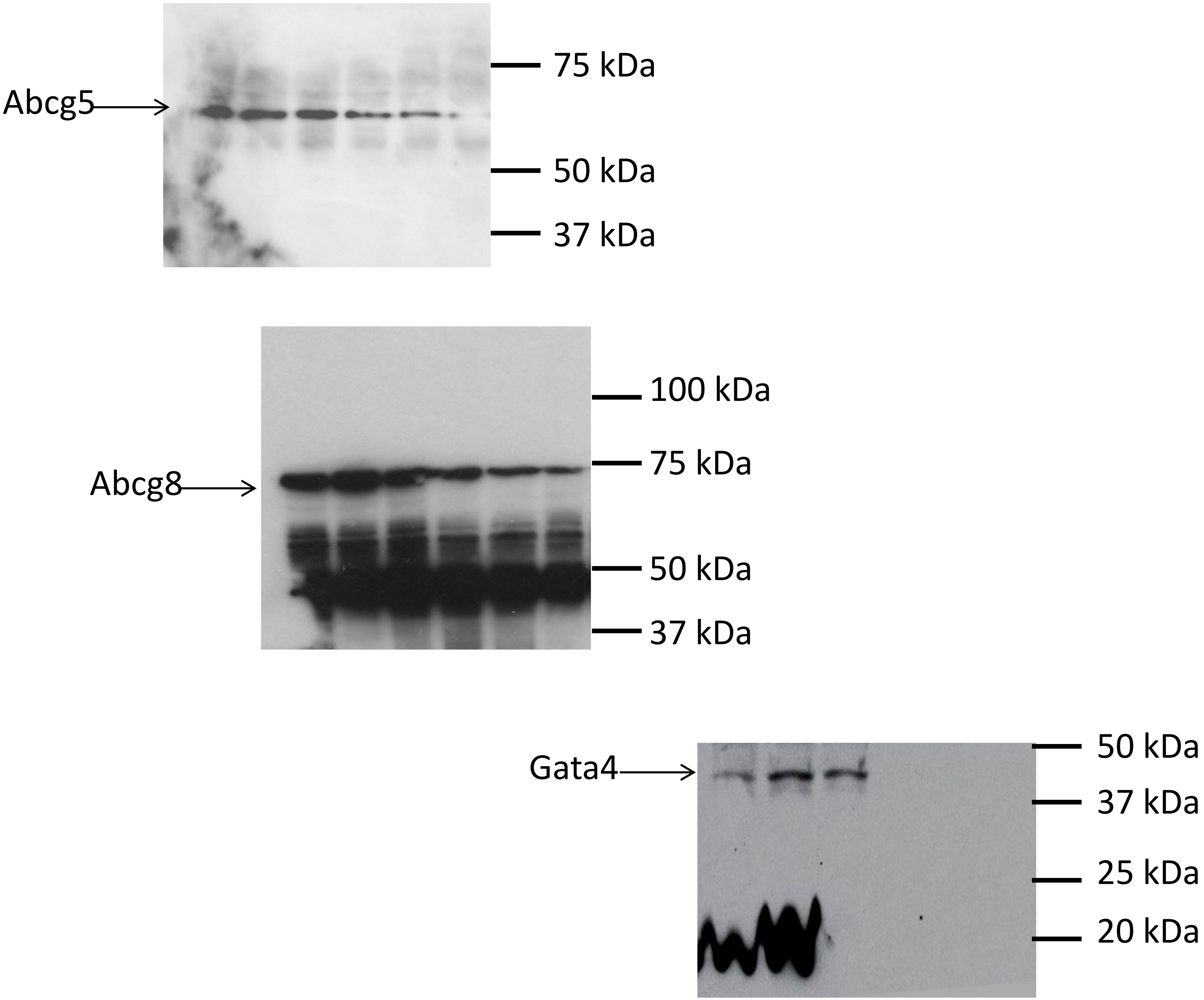

Abcg5
75 kDa
50 kDa
37 kDa
Abcg8
100 kDa
75 kDa
50 kDa
37 kDa
Gata4
50 kDa
37 kDa
25 kDa
20 kDa

Fig 6C continued

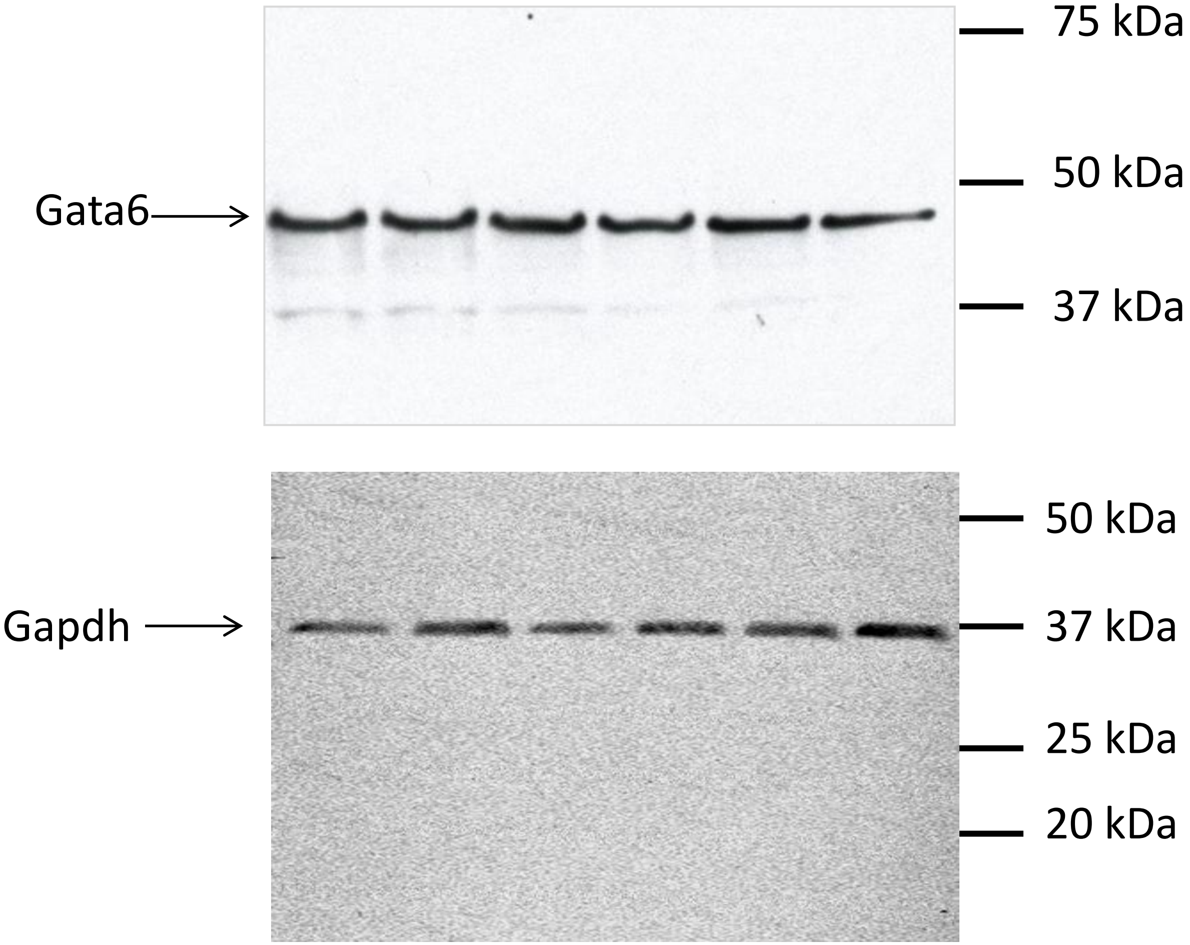

Fig 6D

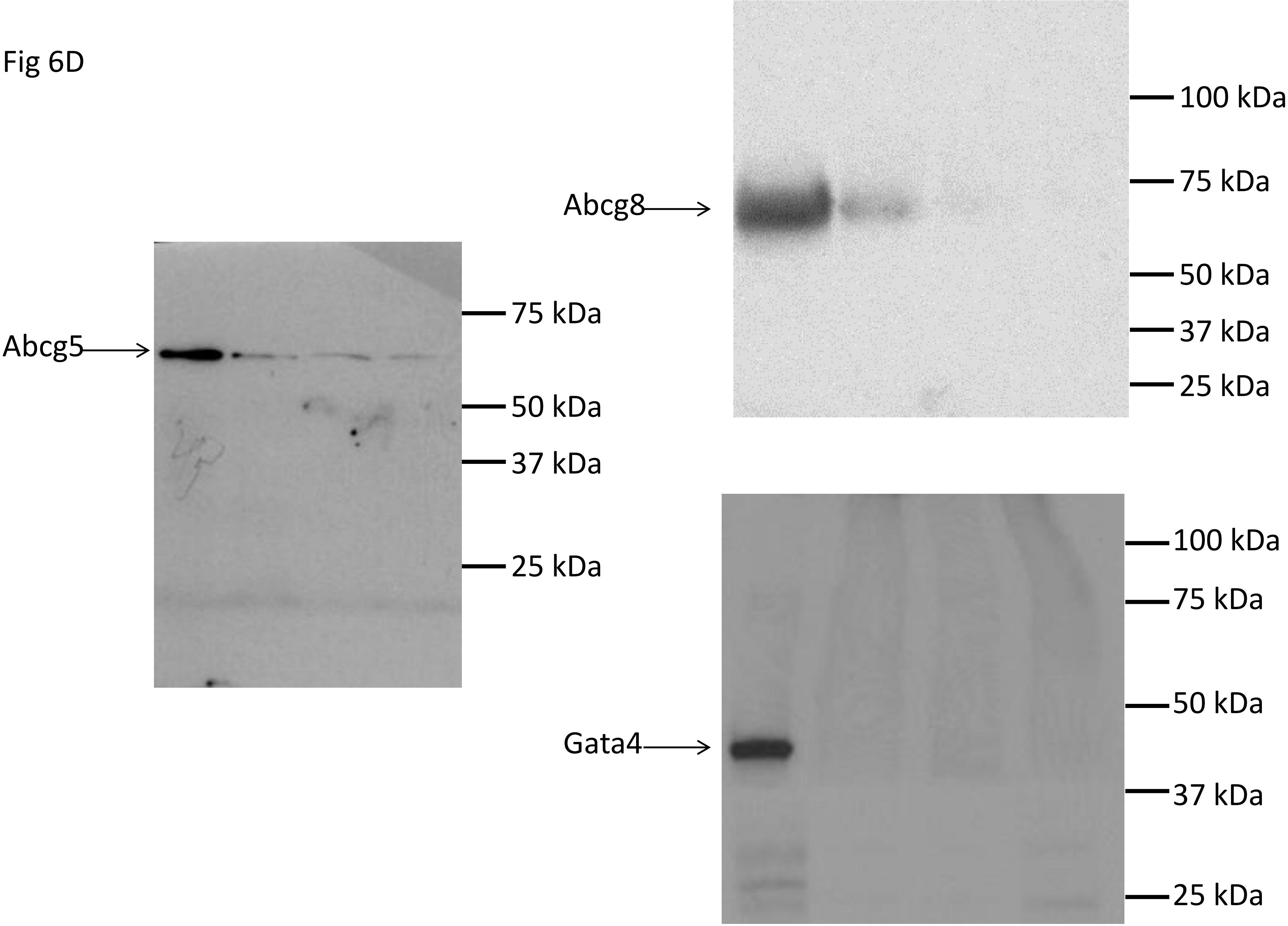
Abcg5
75 kDa
50 kDa
37 kDa
25 kDa
Abcg8
100 kDa
75 kDa
50 kDa
37 kDa
25 kDa
Gata4
100 kDa
75 kDa
50 kDa
37 kDa
25 kDa

Fig 6D continued

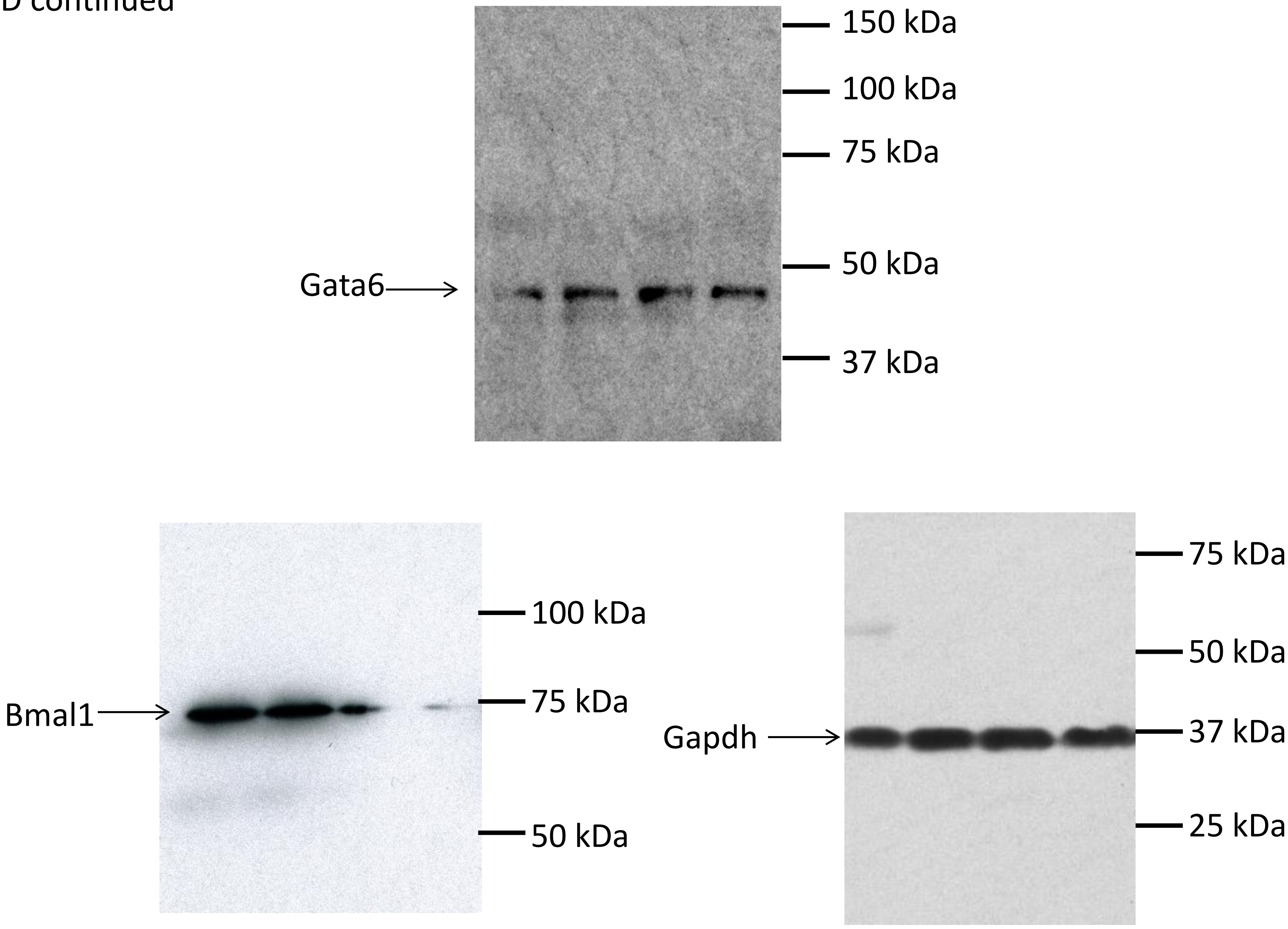

Fig 7B

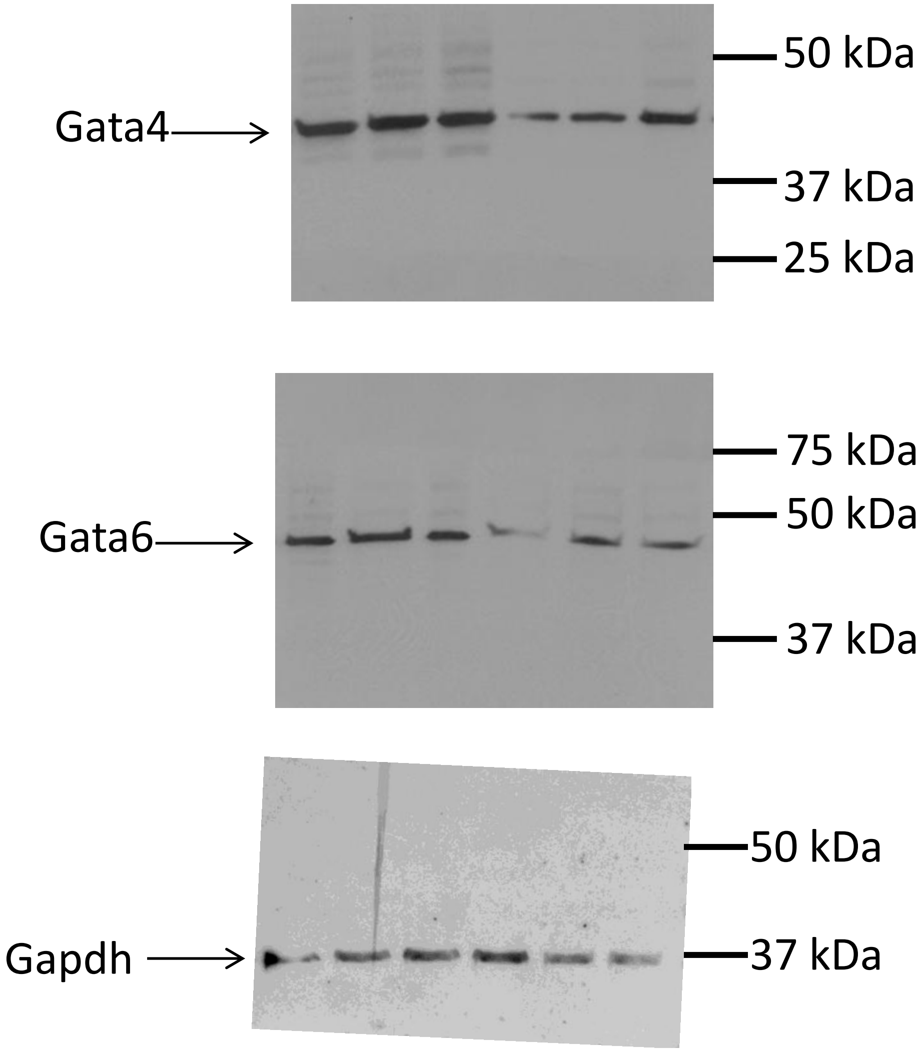
Gata4
50 kDa
37 kDa
25 kDa
Gata6
75 kDa
50 kDa
37 kDa
Gapdh
50 kDa
37 kDa

Fig 7C

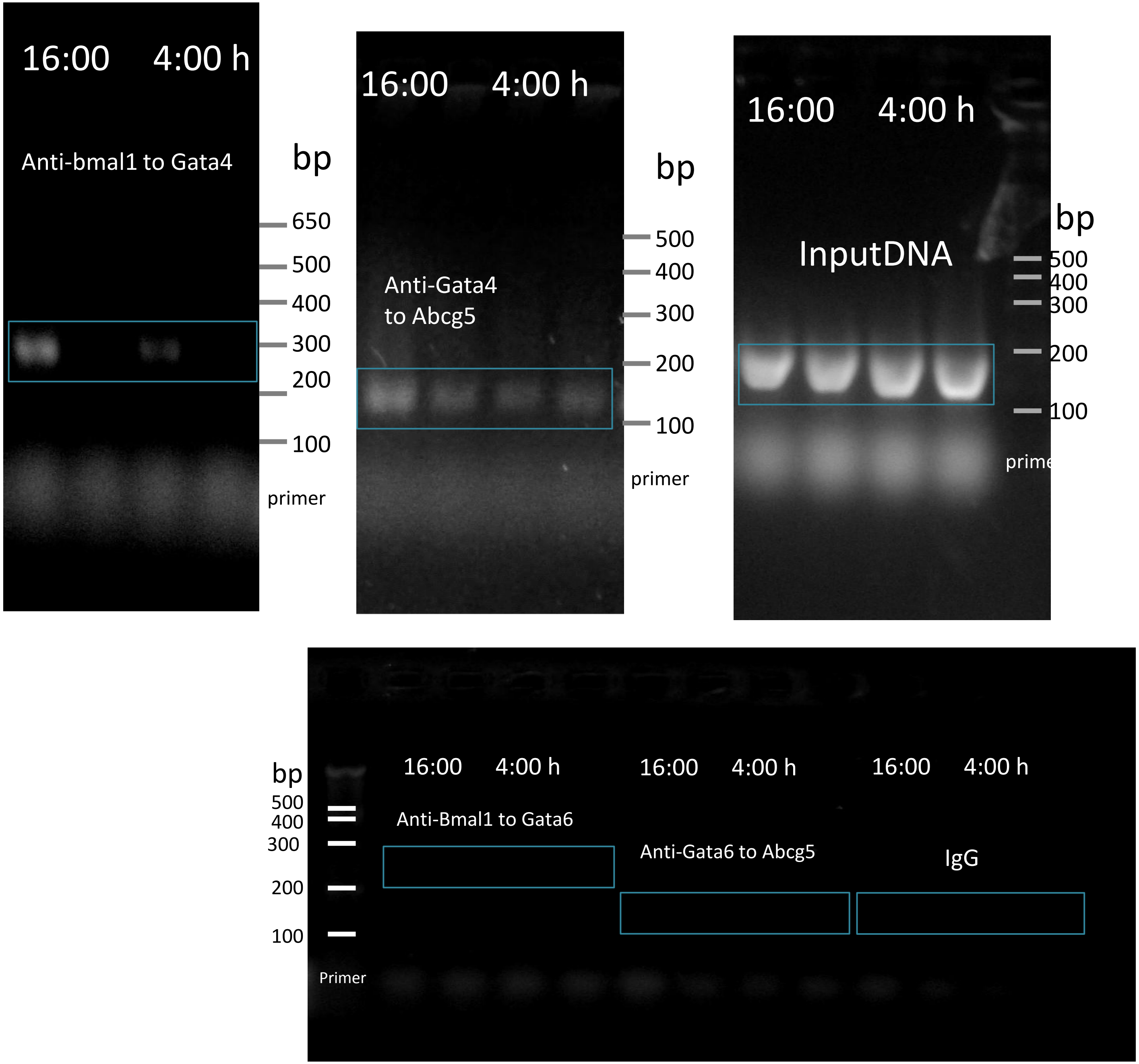
16:00 4:00 h
Anti-bmal1 to Gata4
bp
650
500
400
300
200
100
primer
16:00 4:00 h
Anti-Gata4 to Abcg5
bp
500
400
300
200
100
primer
16:00 4:00 h
InputDNA
bp
500
400
300
200
100
prime
bp
500
400
300
200
100
Primer
16:00 4:00 h
Anti-Bmal1 to Gata6
16:00 4:00 h
Anti-Gata6 to Abcg5
16:00 4:00 h
IgG

Fig 7D

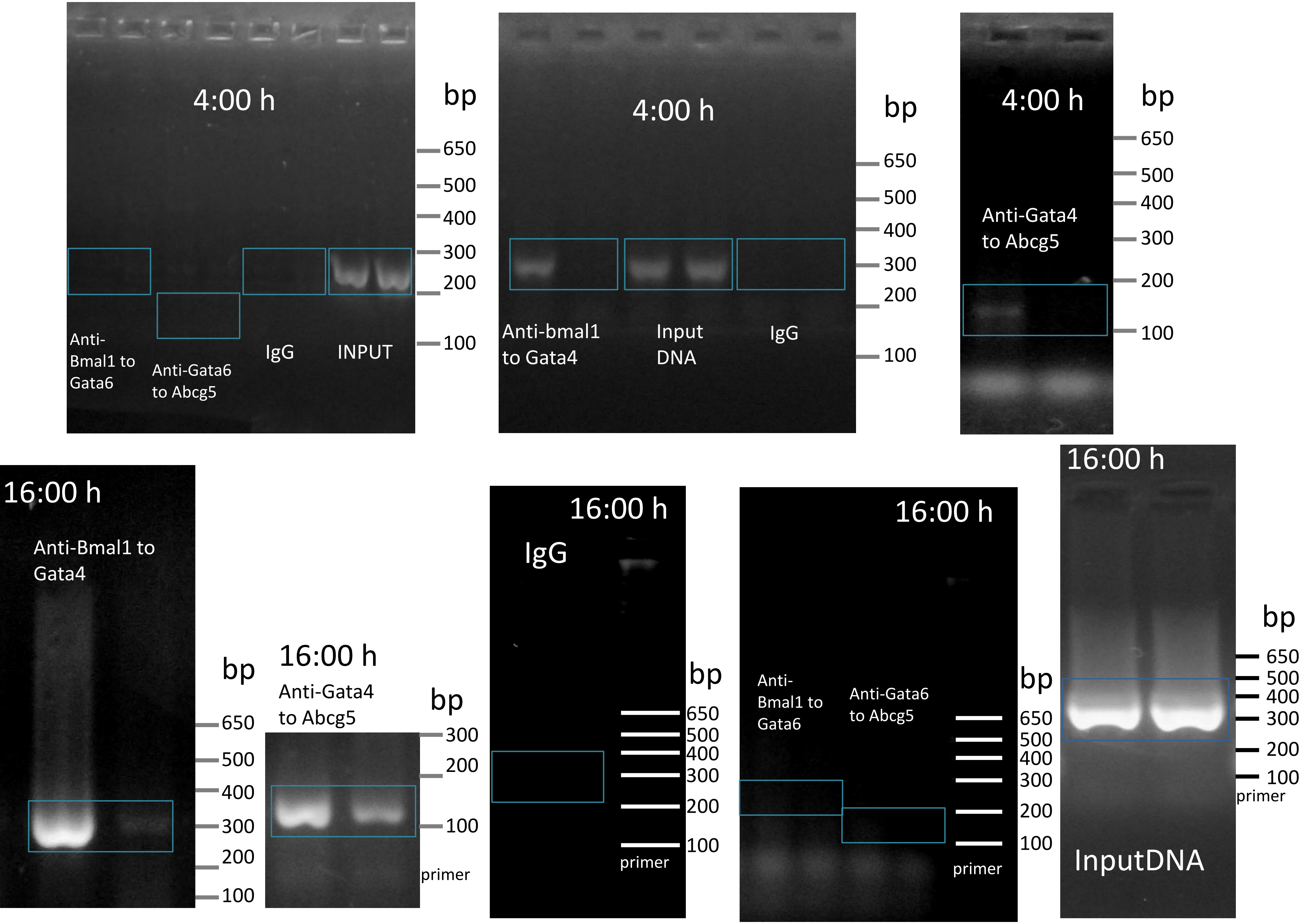
4:00 h
bp
650
500
400
300
200
100
Anti-Bmal1 to Gata6
Anti-Gata6 to Abcg5
IgG
INPUT
4:00 h
bp
650
500
400
300
200
100
Anti-bmal1 to Gata4
Input DNA
IgG
4:00 h
bp
650
500
400
300
200
100
Anti-Gata4 to Abcg5
16:00 h
Anti-Bmal1 to Gata4
bp
650
500
400
300
200
100
16:00 h
Anti-Gata4 to Abcg5
bp
300
200
100
primer
16:00 h
IgG
bp
650
500
400
300
200
100
primer
16:00 h
Anti-Bmal1 to Gata6
Anti-Gata6 to Abcg5
bp
650
500
400
300
200
100
primer
16:00 h
bp
650
500
400
300
200
100
primer
InputDNA

Fig 8A

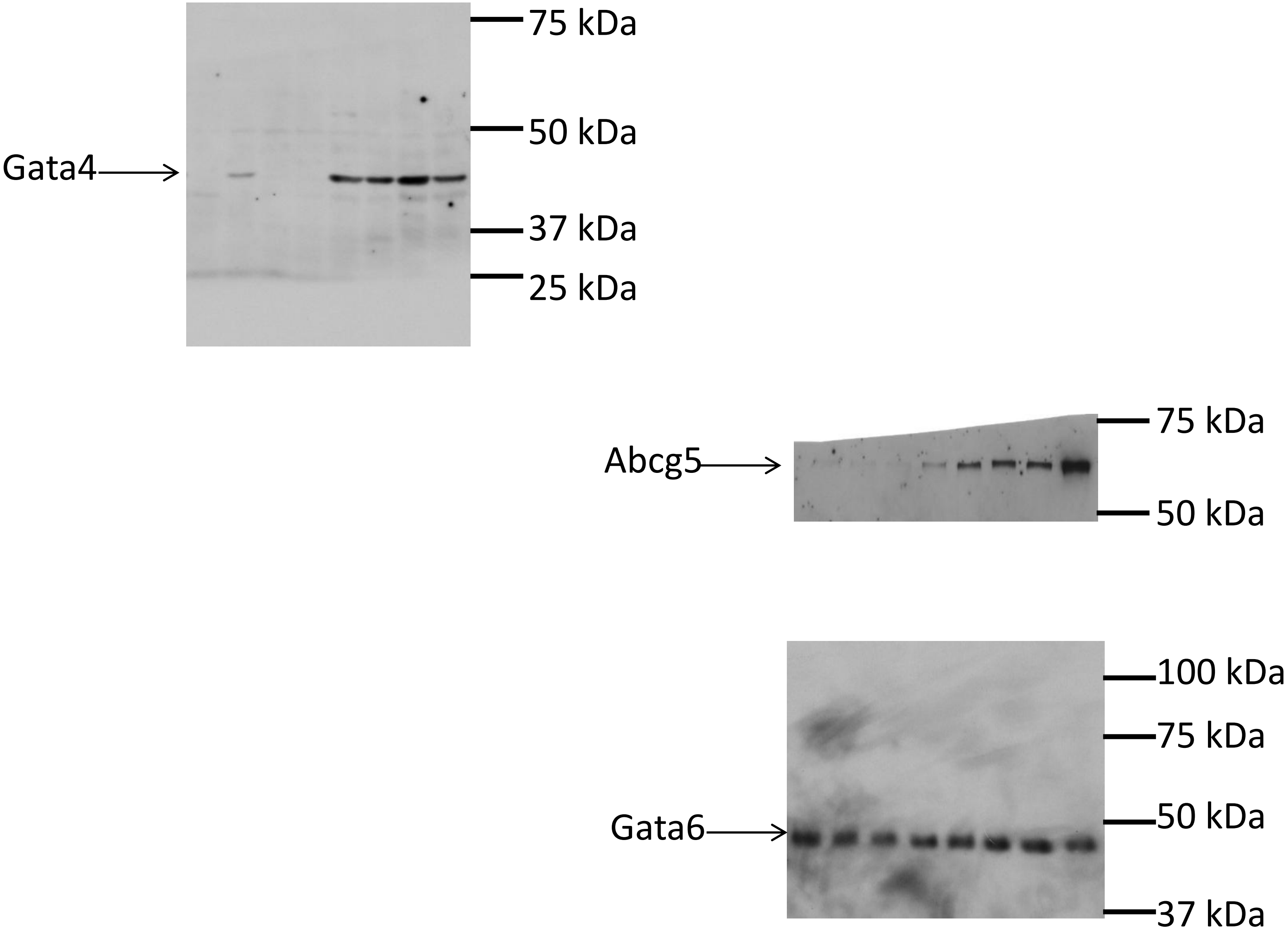
Gata4
75 kDa
50 kDa
37 kDa
25 kDa
Abcg5
75 kDa
50 kDa
Gata6
100 kDa
75 kDa
50 kDa
37 kDa

Fig 8A continued

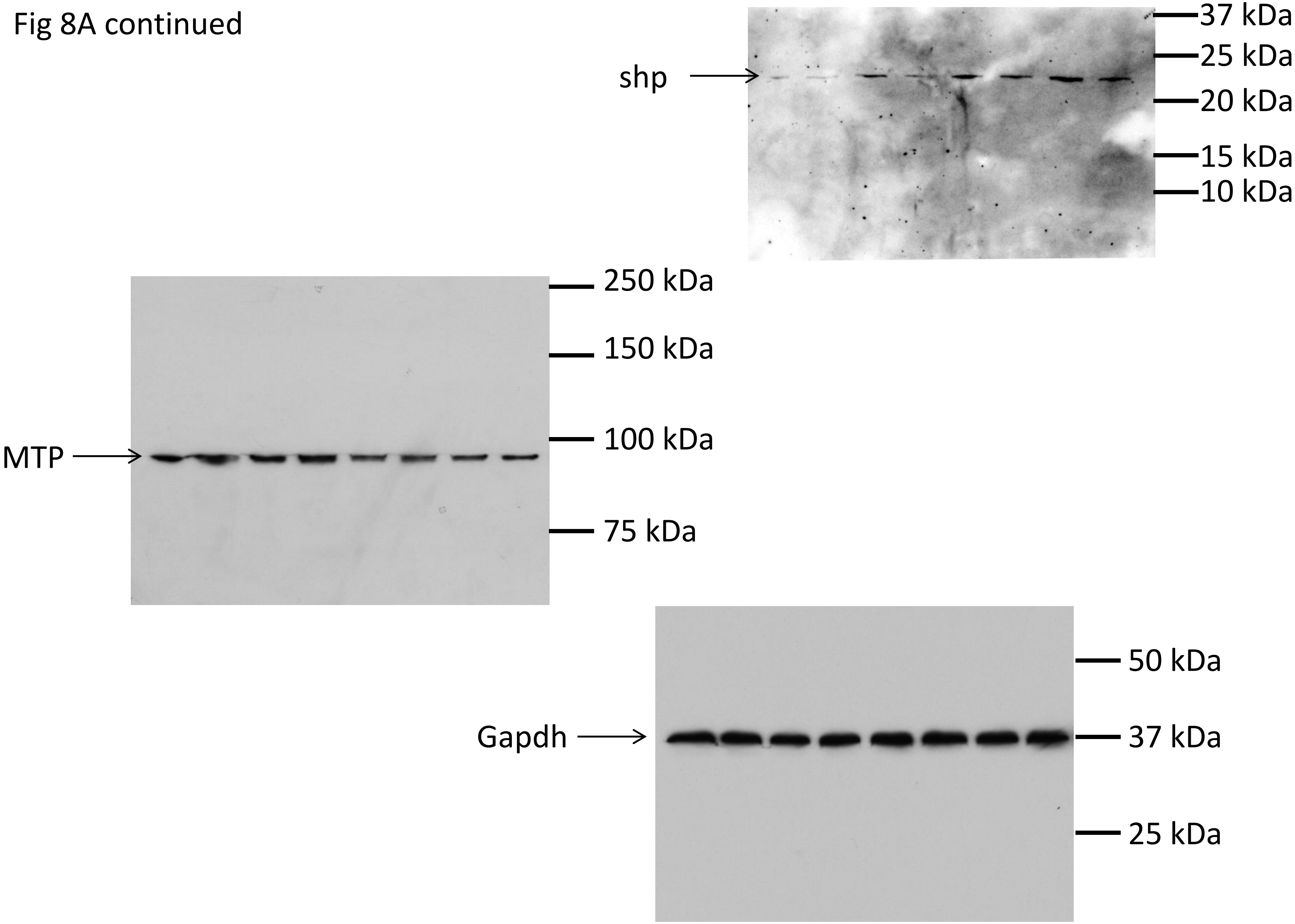

Fig 9H

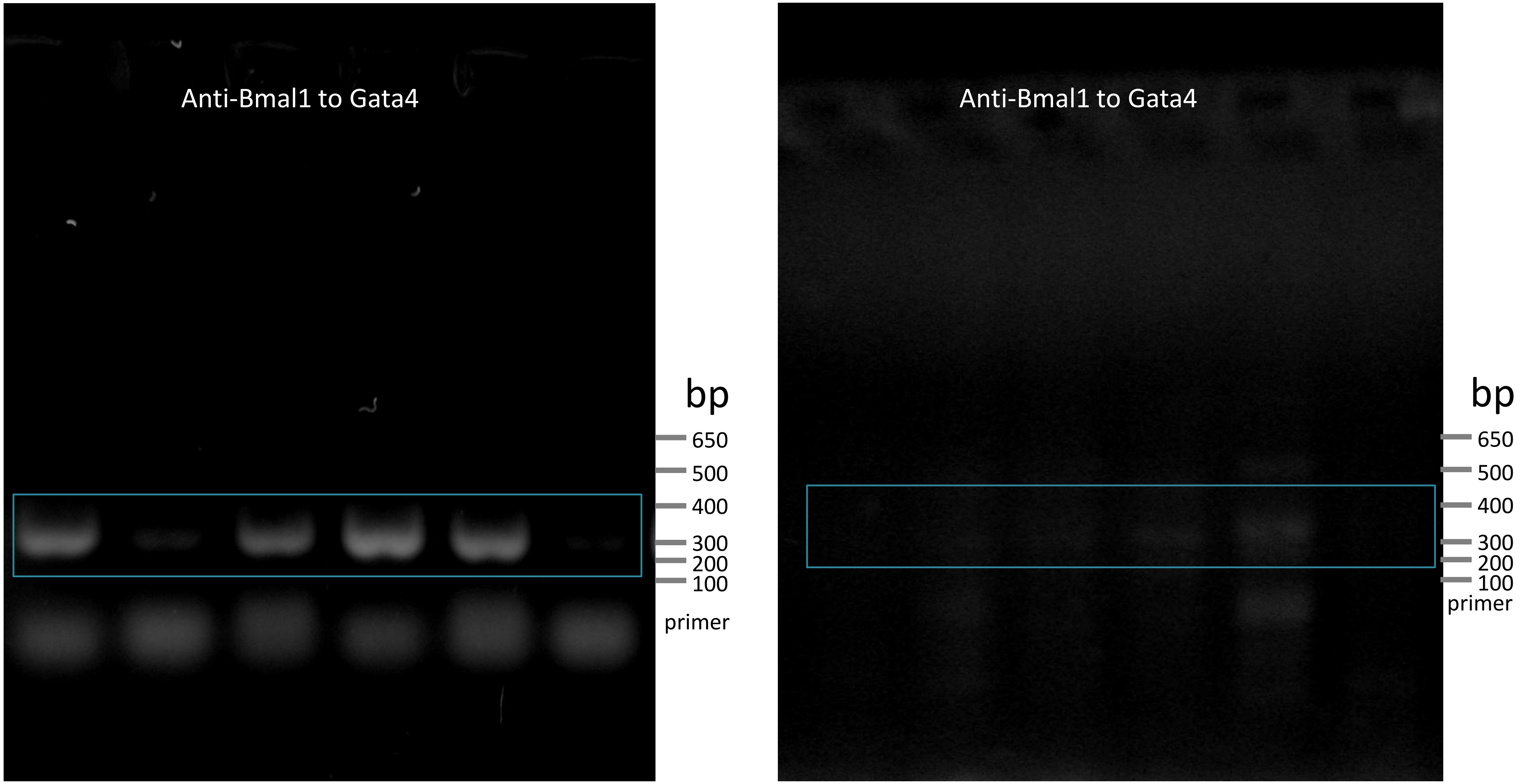
Anti-Bmal1 to Gata4
bp
650
500
400
300
200
100
primer
Anti-Bmal1 to Gata4
bp
650
500
400
300
200
100
primer

Fig 9H continued

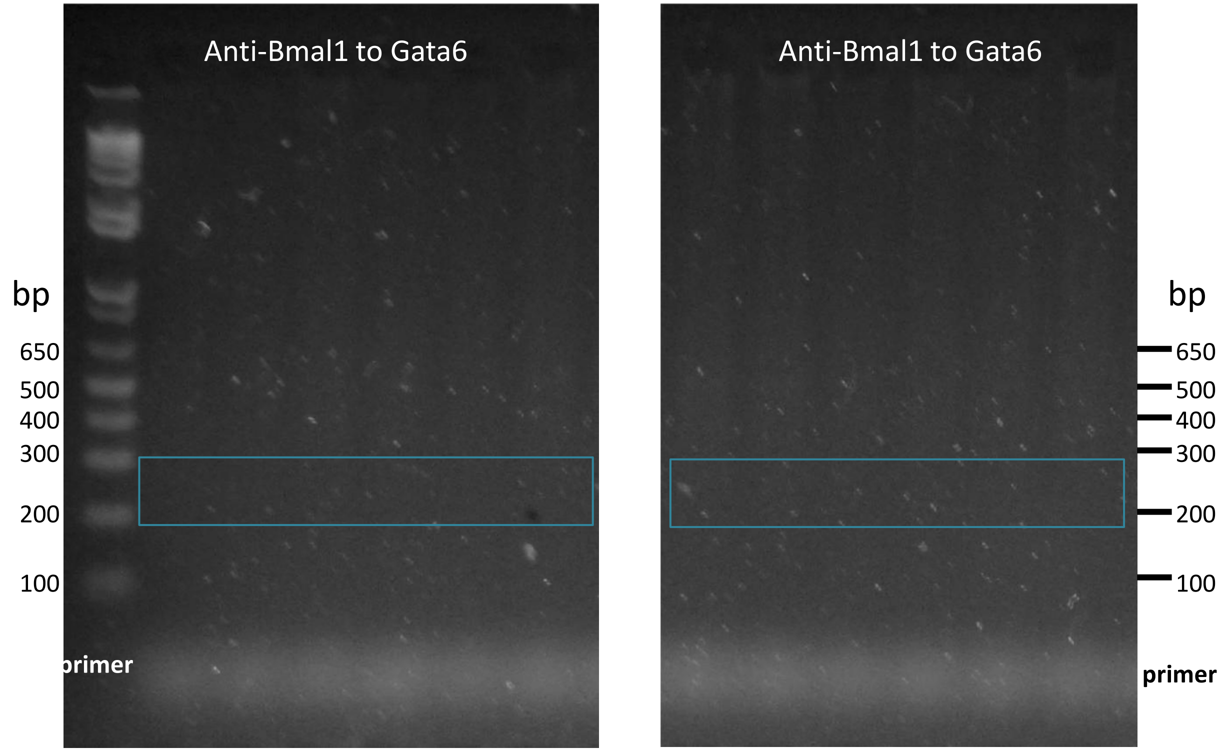

Fig 9H continued

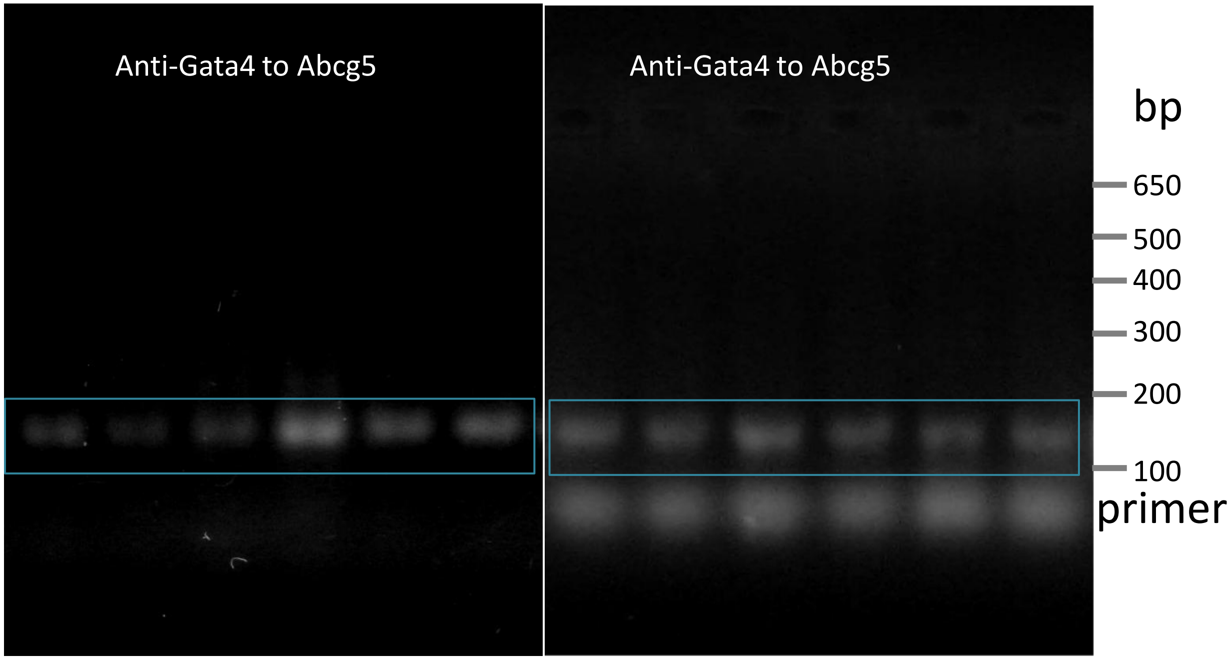

Fig 9H continued

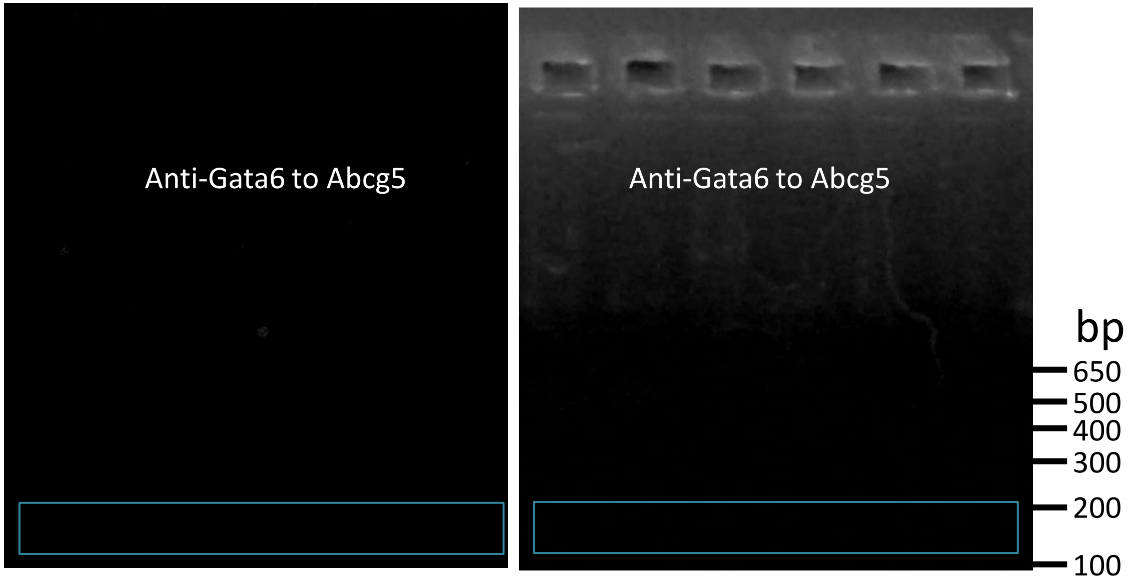

Fig 9H continued

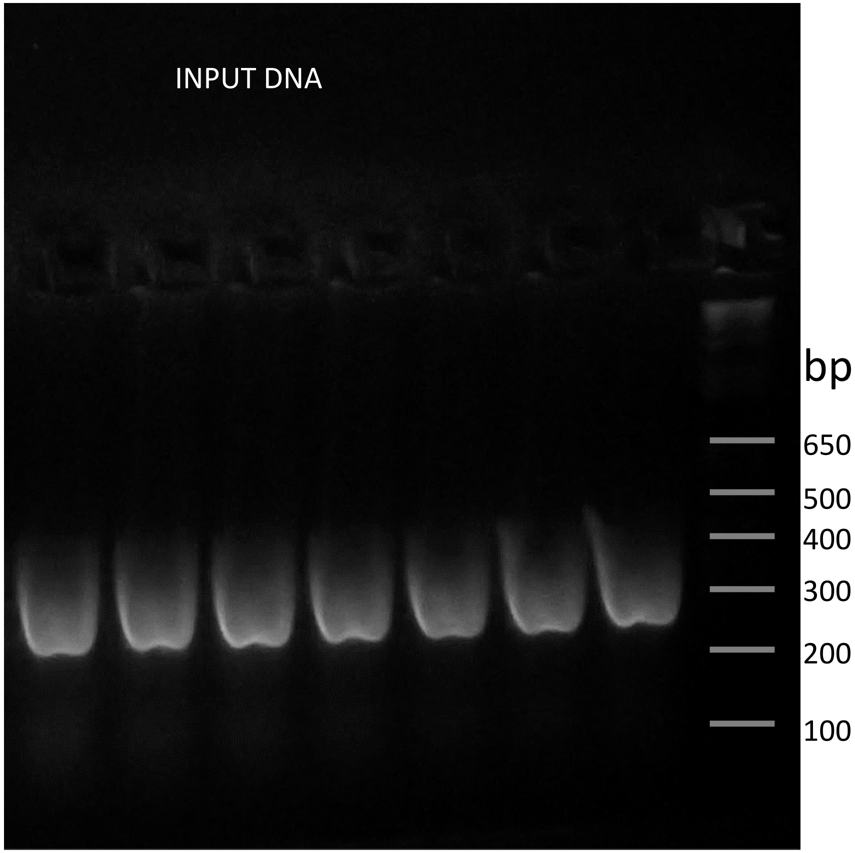

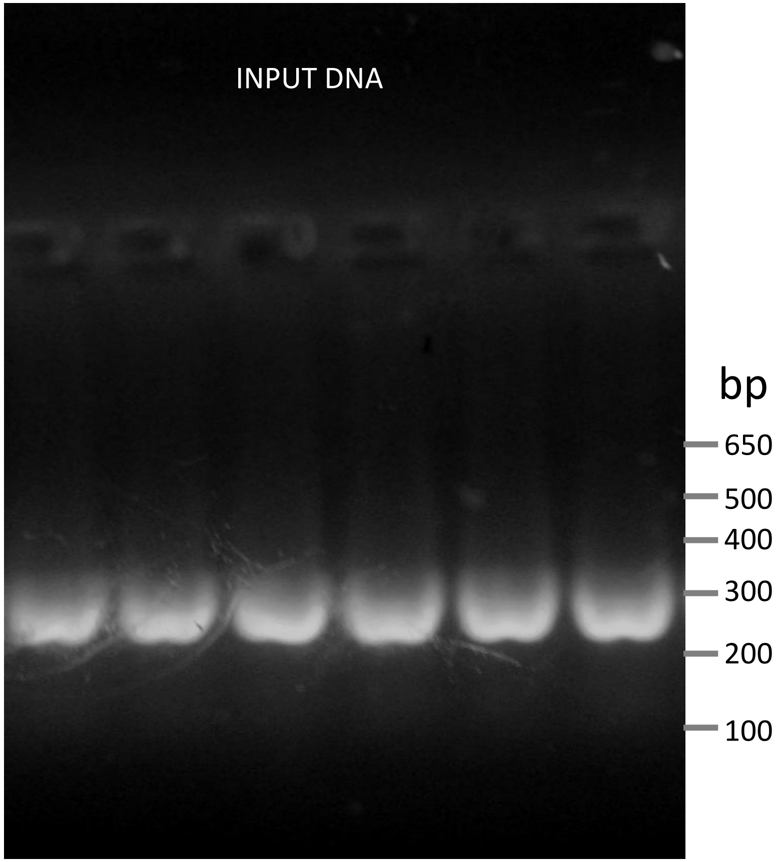

Supplementary Figure 20. Full scans of Western blots and gels in the main text.

Supplementary Table 1: **A**ntibodies and siRNAs used in the study.

**Antigens**	**Cat#**	**Company**
ApoB	23300R	Biodesign
ApoA1	178463	Calbiochem
Mtp	612022	BD Transduction Lab
Shp	Sc-30169	Santa Cruz Biotechnology
Gapah	Ab9485	Abcam
Abcg5	Ab69713	Abcam
Abcg8	Sc-30111	Santa Cruz Biotechnology
Bmal1	Ab93806	abcam
Gata4	Ab84593	Abcam
Gata6	Ab22600	Abcam
**siRNA**	**Cat#**	**Company**
siGata4	Smart pool14463	Dharmacon
siGata6	Smart pool 27409	Dharmacon
siBmal1	Smart pool 11865	Dharmacon

Supplementary Table 2: Oligonucleotide sequences of primers used.

**RT-PCR primers**	Forward (5′-3′)	Reverse (5′-3′)
mGata4	CCTCTCCCAGGAACATCAAA	TGAGGTGCAGATGAGCCATA
mGata6	GAGCTGGTGCTACCAAGAGG	CTGCAAAAGCCCATCTCTTC
mFog1	GCTATATGTGCGCCTTGTCA	TACCAGATCCCGCAGTCTTT
hGATA4	TCCCTCTTCCCTCCTCAAAT	GTCCCATCAGCGTGTAAAGG
hGATA6	GCCTTGCCTGCTATGGAATA	ACCTCATGAACCGACTCAGC
hFOG1	GTTCCTTCCGCAGTACGTGT	GTTGACGTTGCTGAAGGTGA
**Chip assay primers**	Forward (5′-3′)	Reverse (5′-3′)
mAbcg5	GGCAGTCATGACAGAGCTGA	TGGGGTCCTGAAGGAGTTG
mGata4	GCTGGGCCTGTCCTACCT	GCTCCAGCTTGGCTCCAG
mGata6	TGCTGGAAATTGCAACAAAC	AACTCTGCTGAAATAGGTTTCCA
